# Supplementary material for: Non-genetically reprogrammed meta-neutrophils potentiate chemo-immunotherapy against lung metastatic triple-negative breast cancer
Source: Cell Rep Med. 2026 Jun 12;7(7):102868. doi: 10.1016/j.xcrm.2026.102868 (PMC13400185; doi:10.1016/j.xcrm.2026.102868)
Supplement: Document S1. Figures S1–S30 and Tables S1 and S2 [file mmc1.pdf]

Cell Reports Medicine, Volume *7*

Supplemental information

Non-genetically reprogrammed meta-neutrophils potentiate chemo-immunotherapy against lung metastatic triple-negative breast cancer

Meixi Hao, Qifan Hu, Xiuqi Li, Yijun Chen, Siyuan Hou, Chunjing Han, Sijia Chen, Yanyi Li, Kaiming Li, Lingjing Xue, Lulu Zhu, Shanshan Chen, Caoyun Ju, and Can Zhang

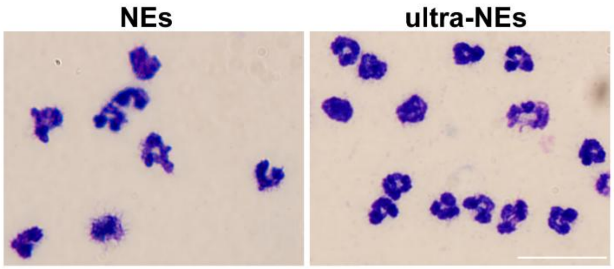

Figure S1. Assessment of NEs Nuclear Morphology Following IFNγ Treatment by Wright-Giemsa Staining.
Related to Figure 1. Sclar Bar: 25 µm.

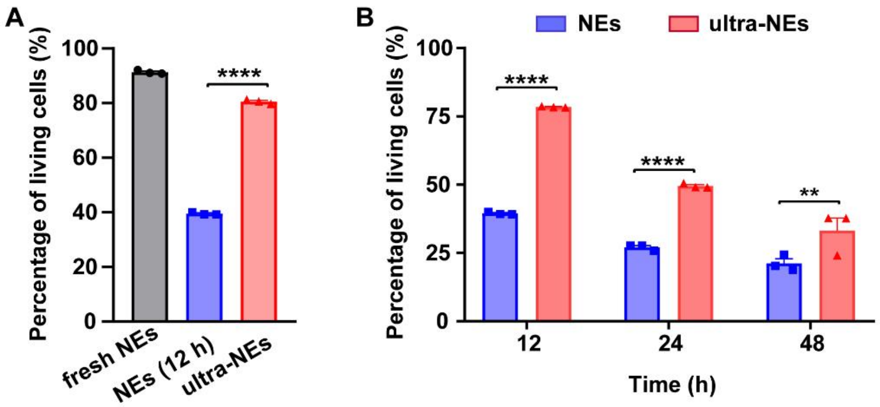

Figure S2. Cell viability of NEs from healthy donors after trained with IFNγ. (A) Cell viability of NEs after IFNγ treated for 12 h. (B) Cell viability of NEs after IFNγ treated for different time. Related to Figure 1. Data were shown as mean ± SEM. n = 3 independent samples, *****P*<0.0001, ***P*<0.01.

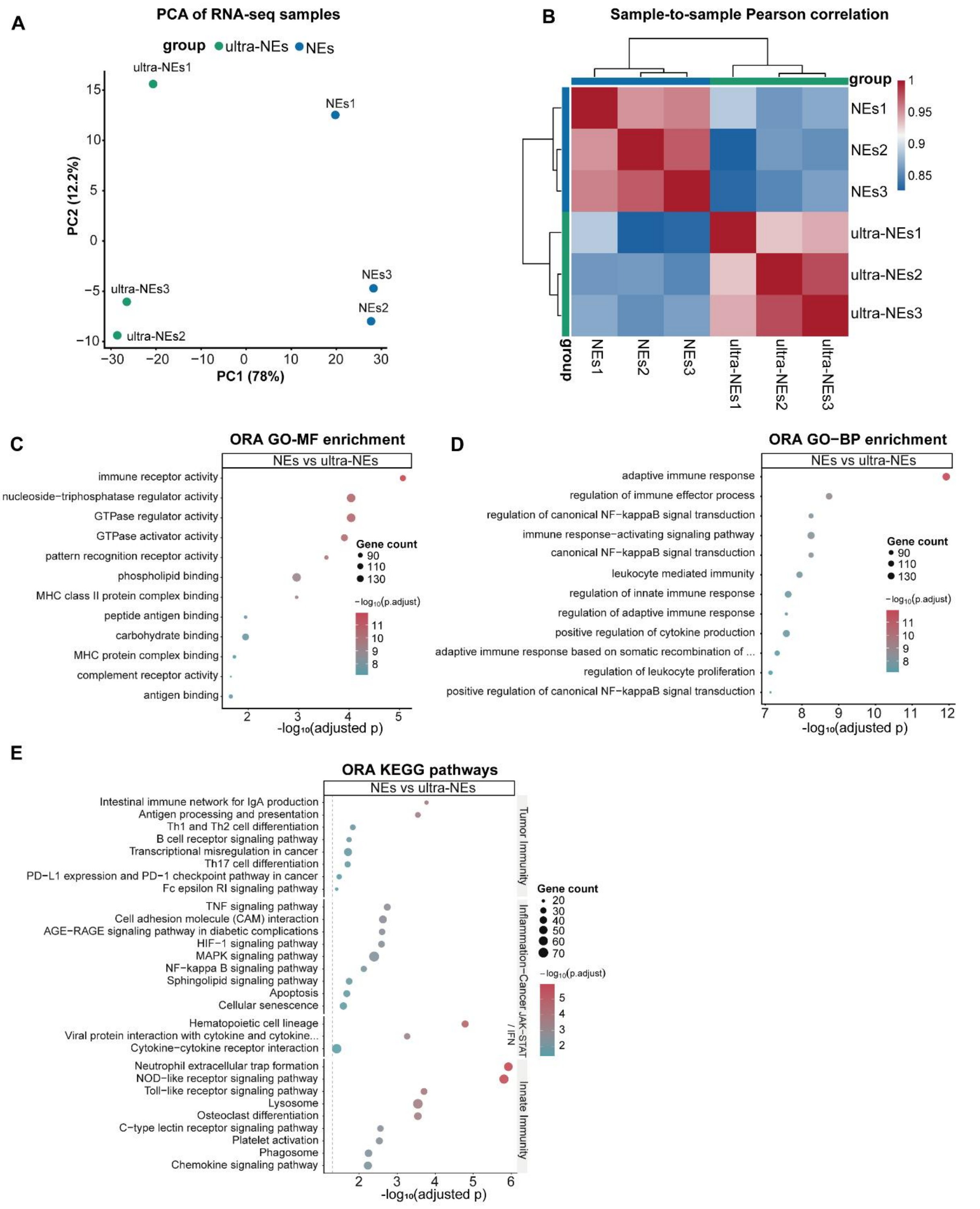

Figure S3. Enhanced RNA-seq analysis overview. (A) Principal component analysis (PCA). (B) Hierarchical clustering analysis. (C) GO-MF enrichment analysis. (D) GO-BP enrichment analysis. (E) KEGG pathway analysis. Related to Figure 1.

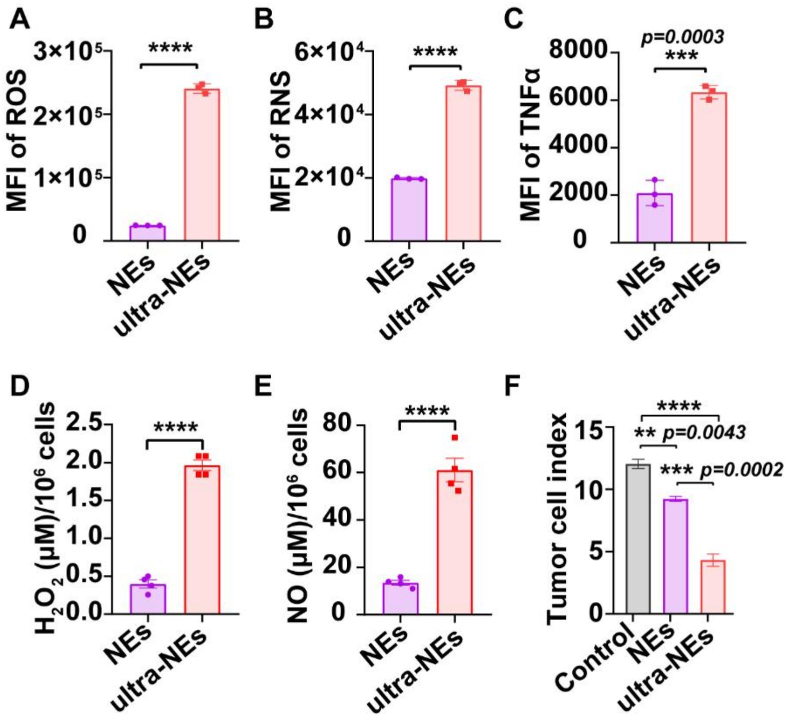

Figure S4. Toxic effector molecules expression and tumor cell index of ultra-NEs. Quantification of ROS (A), RNS (B), TNFα (C), hydrogen peroxide (H2O2, D), and nitric oxide (NO, E) secreted by ultra-NEs. H2O2 is one of the important effector molecules of ROS. NO is the main source of RNS. (F) Quantification of tumor cell index. Related to Figure 1. Data were analyzed by two-tailed Student's t test (A-E), or one-way ANOVA test with Tukey's correction (F). All data were shown as mean ± SEM (n = 4 samples per group), ****P<0.0001.

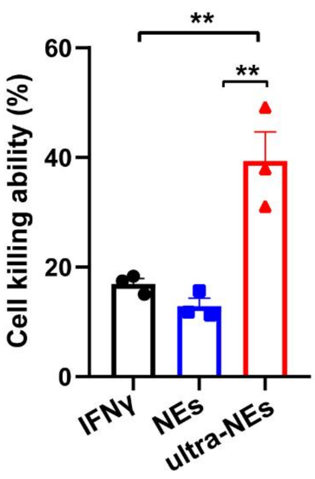

Figure S5. The tumor-killing ability of IFNγ and ultra-NEs. Related to Figure 1. Data were analyzed by one-way ANOVA test with Tukey's correction and shown as mean ± SEM (n = 3 samples per group). ***P*<0.005.

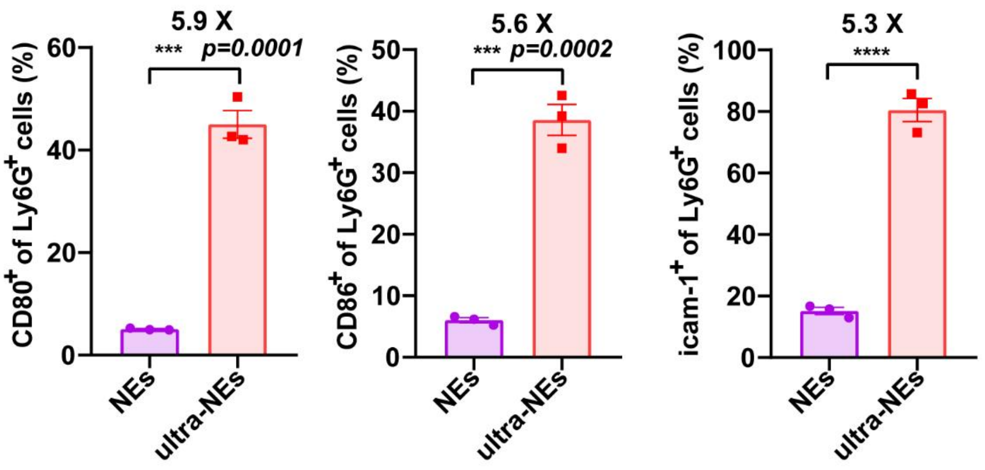

Figure S6. Quantification of the expressions of CD80, CD86 or icam-1 in ultra-NEs. Related to Figure 1. Data were analyzed by two-tailed Student's t test and shown as mean ± SEM (n = 4 samples per group). ****P<0.0001.

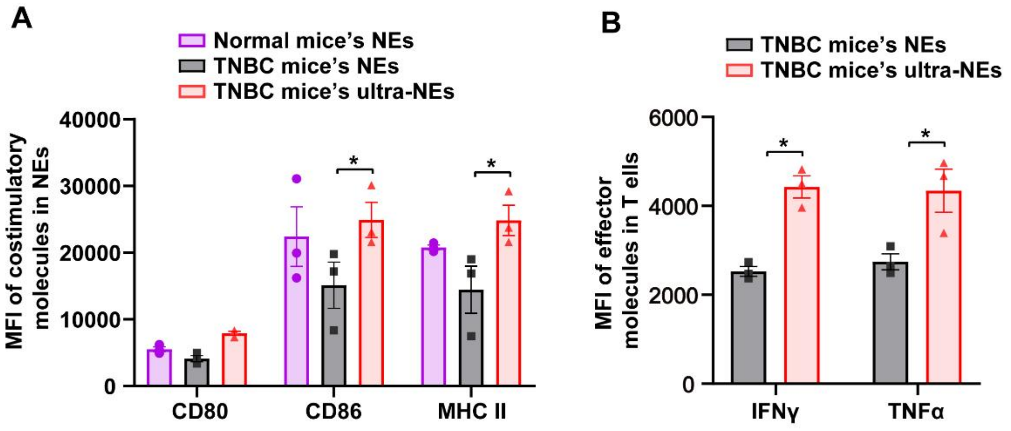

Figure S7. The antigen-presenting and T cell activation ability of NEs derived from tumor-bearing mice after IFNγ training. (A) Quantification of the expressions of CD80, CD86 or MHC II in ultra-NEs derived from tumor-bearing mice. (B) Quantification of TNFα and IFNγ expression of tumor-infiltrating $CD8^+$T cells co-cultured with ultra-NEs derived from tumor-bearing mice. Related to Figure 2. Data were analyzed by one-way ANOVA test with Tukey's correction and shown as mean ± SEM (n = 3 samples per group). *$P<0.01$.

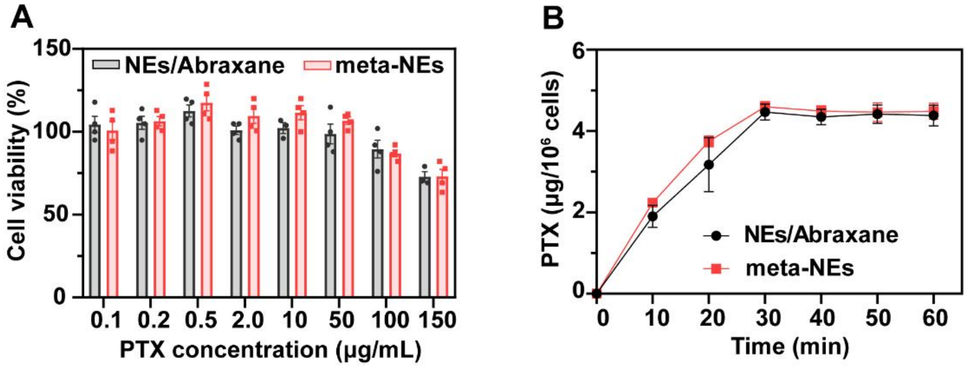

Figure S8. Screening of the concentration and incubation time of Abraxane. (A) In vitro cytotoxicity of different concentration of Abraxane on ultra-NEs or NEs for 12 hours (n = 4 independent samples). (B) Loading efficiency at different time of NEs/Abraxane and meta-NEs. n= 3 samples per group. Related to Figure 3. Data were shown as mean ± SEM.

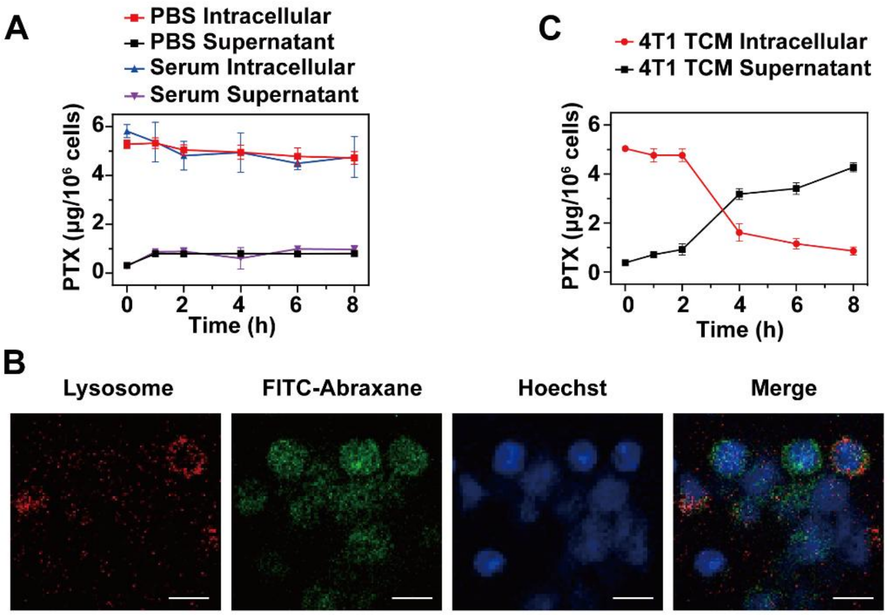

Figure S9. Stability and drug release of meta-NEs. (A) In vitro stability of meta-NEs after incubation with PBS or 50% serum over time (n = 3 samples per group). (B) The confocal images of the uptake pathway of meta-NEs. Abraxane were labeled with FITC (Green), lysosomes were labeled with LysoTracker Red DND-99 (Red), and cell nuclei were labeled with Hoechst 33342 (Blue). Scale bar: 20 μm. (C) Release of PTX from meta-NEs after incubation with 4T1 TCM over time (n = 3 samples per group). Related to Figure 3.

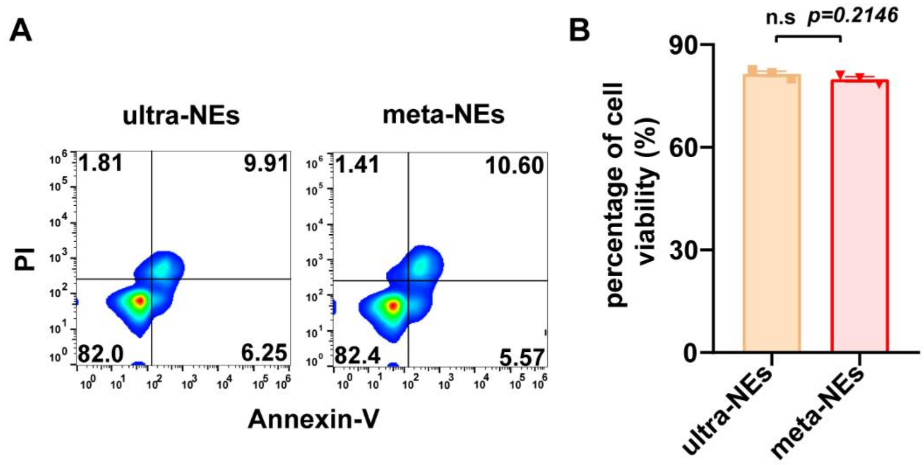

Figure S10. Cell viability of meta-NEs. (A) Representative image of apoptosis of ultra-NEs before and after drug loading determined by flow cytometry. (B) Quantification of cell viability (n = 3 samples per group). Related to Figure 3. Data were analyzed by two-tailed Student's t test and shown as mean ± SEM. n.s denotes no significant difference.

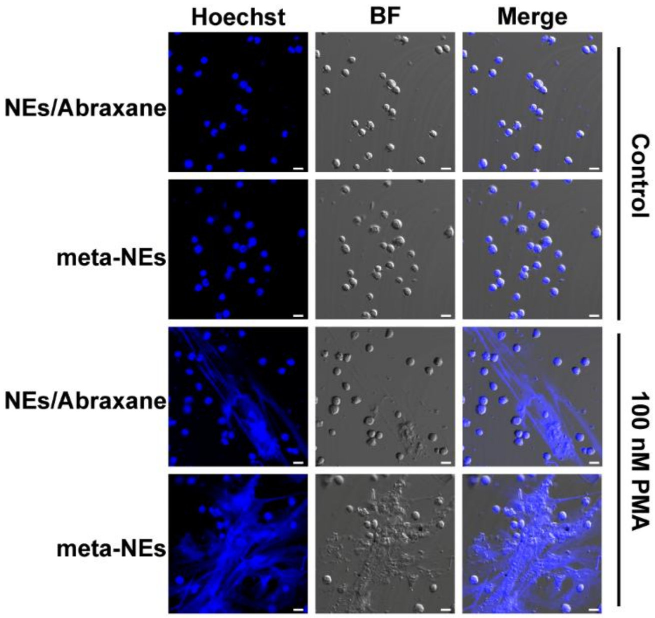

Figure S11. Representative image of the release of NETs from meta-NEs after treatment with 100 nM PMA for 4 hours in vitro. Scale bars, 10 μm. Related to Figure 3.

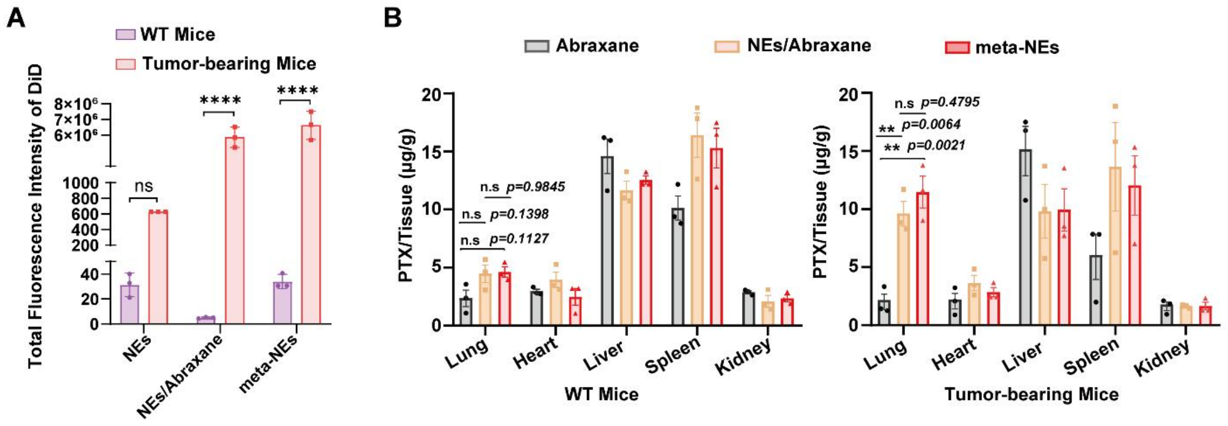

Figure S12. Distribution of NEs and PTX in WT mice and tumor-bearing mice. (A) Quantitative analysis of DiD fluorescence in Figure 3I (n = 3 mice per group). (B) Amounts of PTX amounts in different tissues harvested from WT mice and 4T1-Luci-bearing mice after intravenous injection of different Abraxane formulations for 12 hours (n = 3 mice per group). Related to Figure 3.

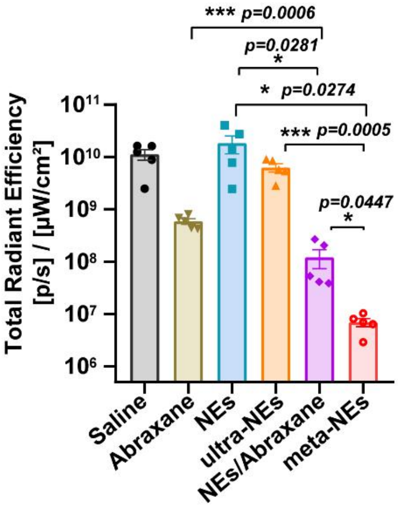

Figure S13. Total radiant efficiency of lung bioluminescence from the tumor-bearing mice after treatment (N= 5 mice per group). Related to Figure 4. Data were shown as the mean ± SEM and analyzed by one-way ANOVA test with Tukey's correction.

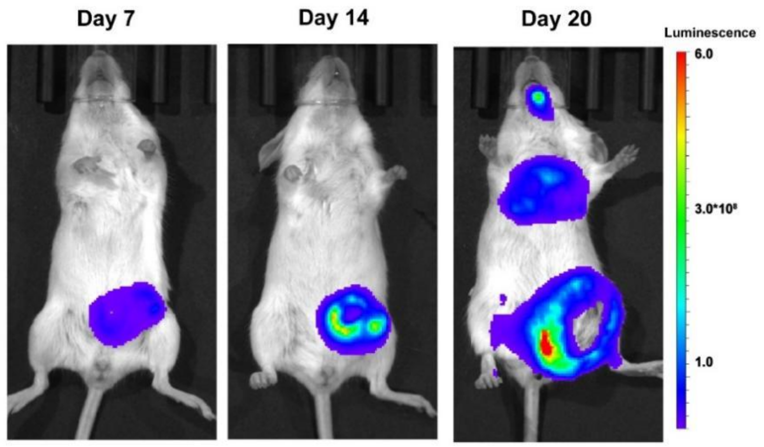

Figure S14. The bioluminescence images of spontaneous TNBC lung metastasis mouse model. Related to Figure 4.

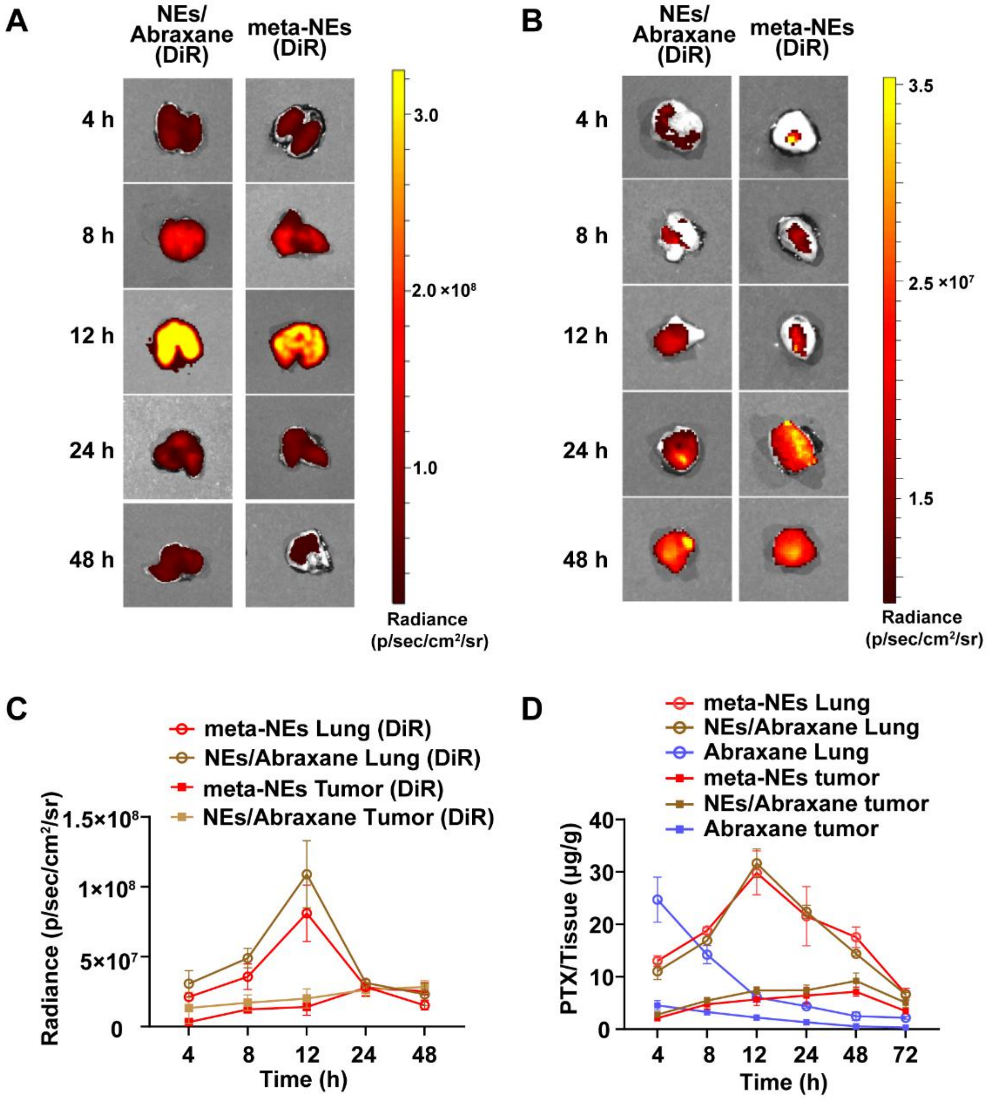

Figure S15. Distribution of meta-NEs in primary tumor and lung metastasis foci. Ex vivo images of the lung metastasis loci (A) and primary tumor (B) collected from mice receiving DiR-labeled meta-NEs and NEs/Abraxane at 4, 8, 12, 24 and 48-hours post-injection. (C) The fluorescent quantification of A) and B). (D) Quantification of PTX in primary tumor and lung metastatic foci after intravenous injection of different Abraxane formulations with the dosage of PTX at 2.5 mg/kg. n = 3 mice per group. Error bars denote SEM. Related to Figure 4.

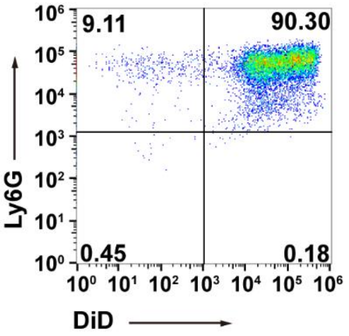

Figure S16. Purity of meta-NEs sorted from lungs of 4T1 metastasis bearing mice. Meta-NEs were labeled with DiD before intravenous injection. Related to Figure 5.

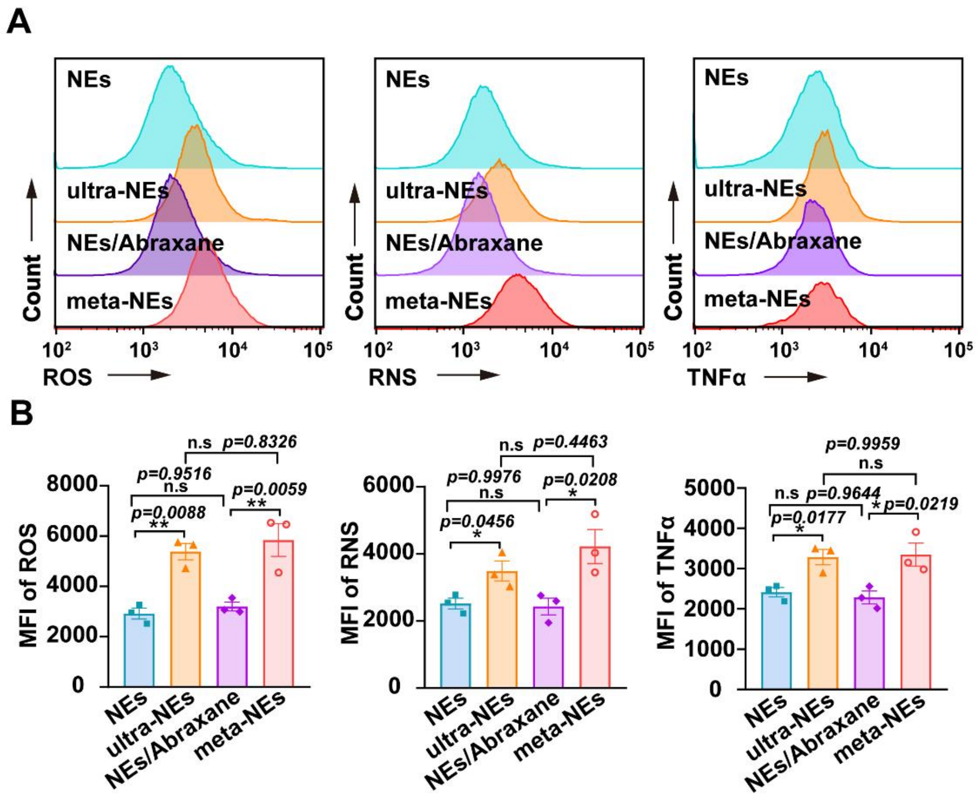

Figure 17. Flow cytometry analysis of ROS, RNS, and TNFα expression of reinfused NEs in lung metastatic foci from spontaneous TNBC lung metastasis mouse model. (A) Representative flow cytometry analysis of ROS, RNS, and TNFα expression. (B) Quantification of ROS, RNS, and TNFα expression. n = 3 mice per group. Related to Figure 5. Data were analyzed by one-way ANOVA test with Tukey's correction and shown as the mean ± SEM. n.s denotes no significant difference.

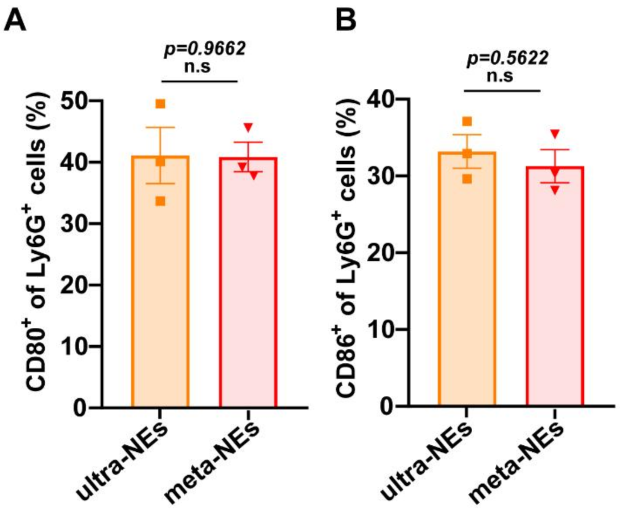

Figure S18. Quantification of co-stimulation molecules of meta-NEs. (A) Quantification of CD80 expression of meta-NEs. (B) Quantification of CD86 expression of meta-NEs. Ultra-NEs were used as a control. Related to Figure 6. Data were analyzed by two-tailed Student's t test and shown as mean ± SEM (n = 3 samples per group). n.s denotes no significant difference.

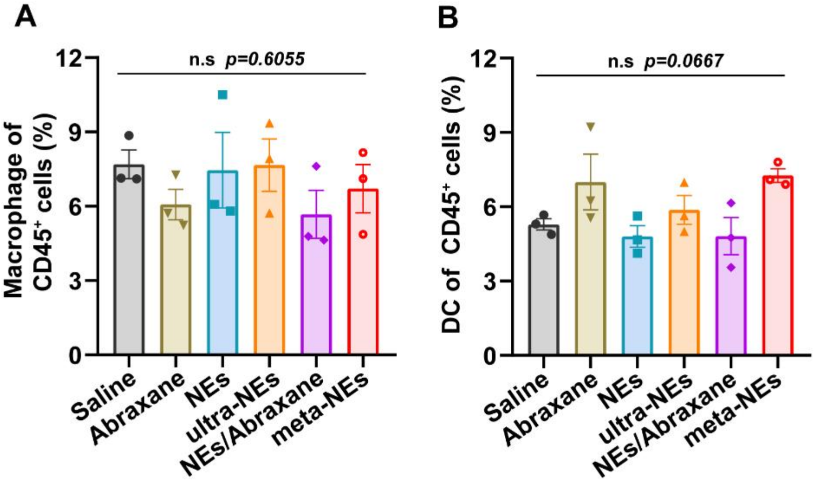

Figure S19. The proportion of macrophage and DCs in lung metastatic foci. Related to Figure 6. Data were analyzed by one-way ANOVA test with Tukey's correction and shown as mean ± SEM (n = 3 mice per group). n.s denotes no significant difference.

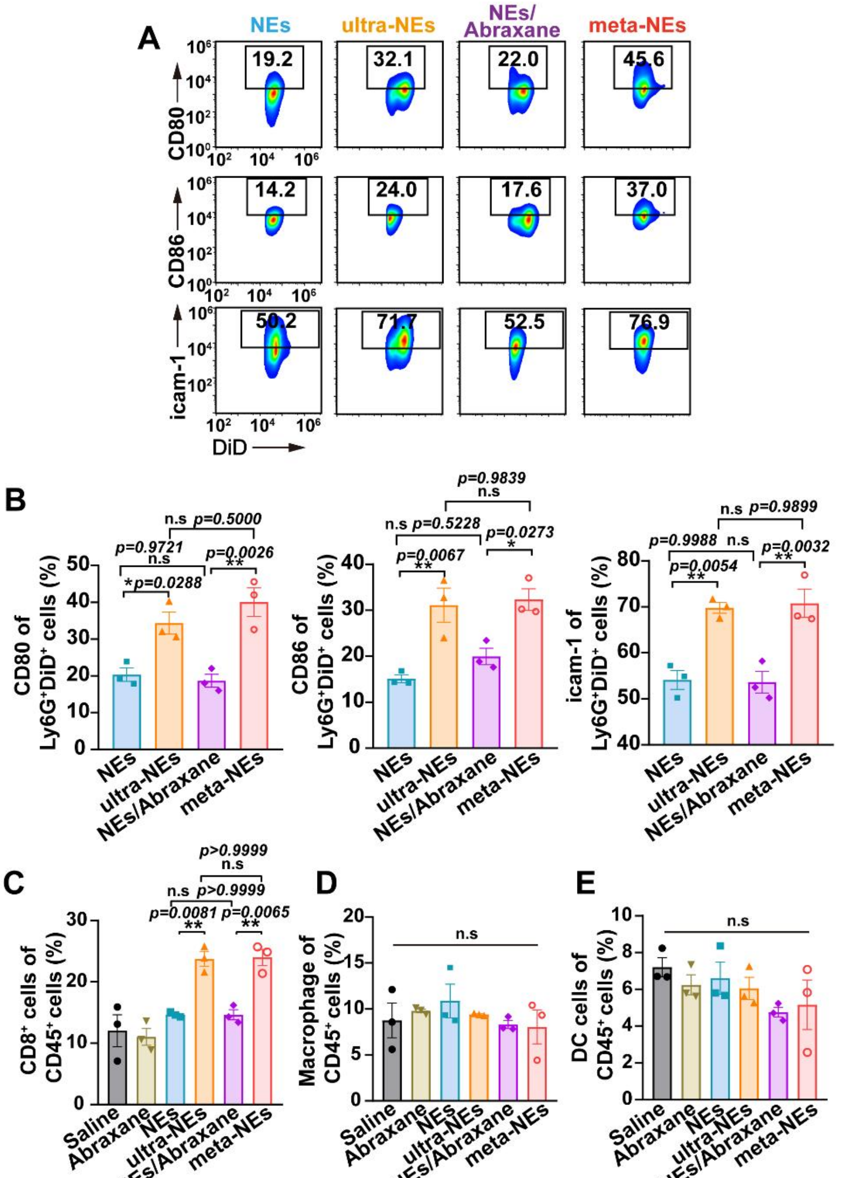

Figure S20. The expressions of CD80, CD86, and icam-1 of reinfused NEs and the frequency of infiltrated immune cells in lungs metastatic foci from spontaneous TNBC lung metastasis bearing mice. (A) Representative flow cytometry analysis of CD80, CD86, and icam-1 expression. (B) Quantification of CD80, CD86, and icam-1 expression. (C-E) The frequency of infiltrated $CD8^{+}$T cells (C), macrophages (D), and DCs (E) in spontaneous lung metastatic foci. n = 3 mice per group. Related to Figure 6. Data were analyzed by one-way ANOVA test with Tukey's correction and shown as the mean ± SEM. n.s denotes no significant difference.

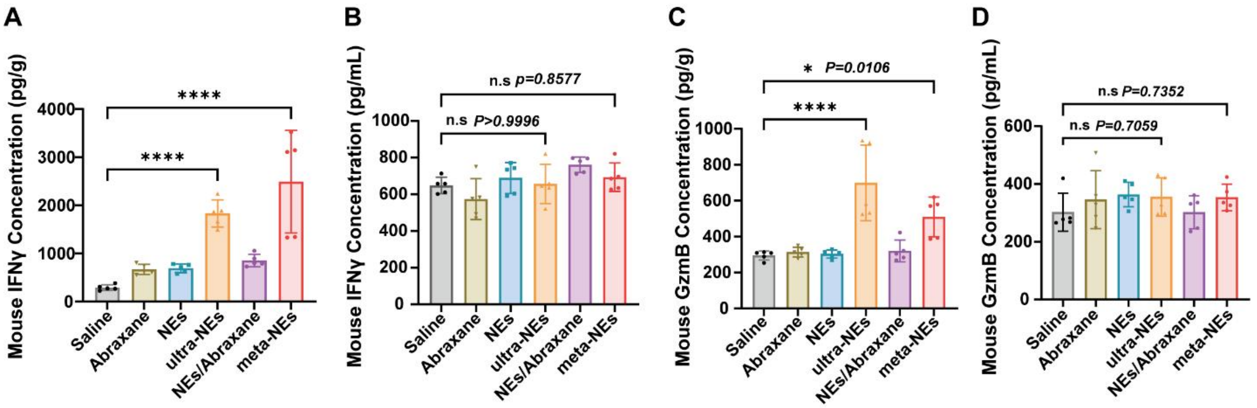

Figure S21. ELISA analysis of secreted cytokine levels in peripheral blood and tumor tissues of mice (n = 5 mice per group). (A) IFNγ levels in tumor tissues. (B) IFNγ levels in serum. (C) GZMB levels in tumor tissues. (D) GZMB levels in serum. Related to Figure 6. Data were analyzed by one-way ANOVA test with Tukey's correction and shown as the mean ± SEM. ****P < 0.0001, n.s denotes no significant difference.

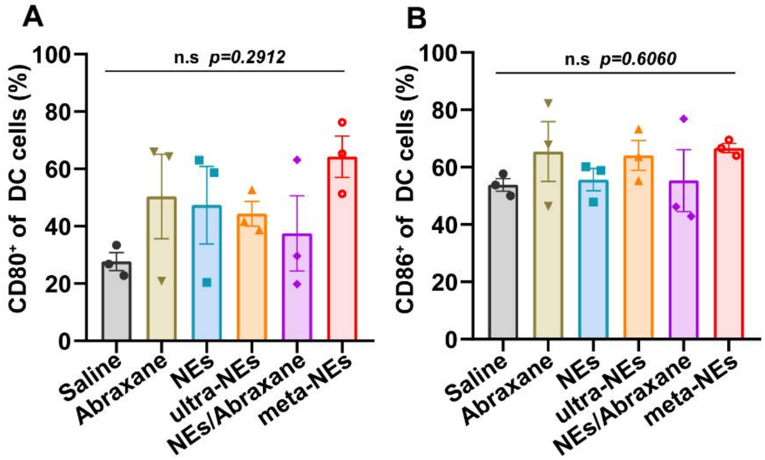

Figure S22. Expressions of CD80 and CD86 of DCs in lung metastatic foci. Related to Figure 6. Data were analyzed by one-way ANOVA test with Tukey's correction and shown as mean ± SEM (n = 3 mice per group). n.s denotes no significant difference.

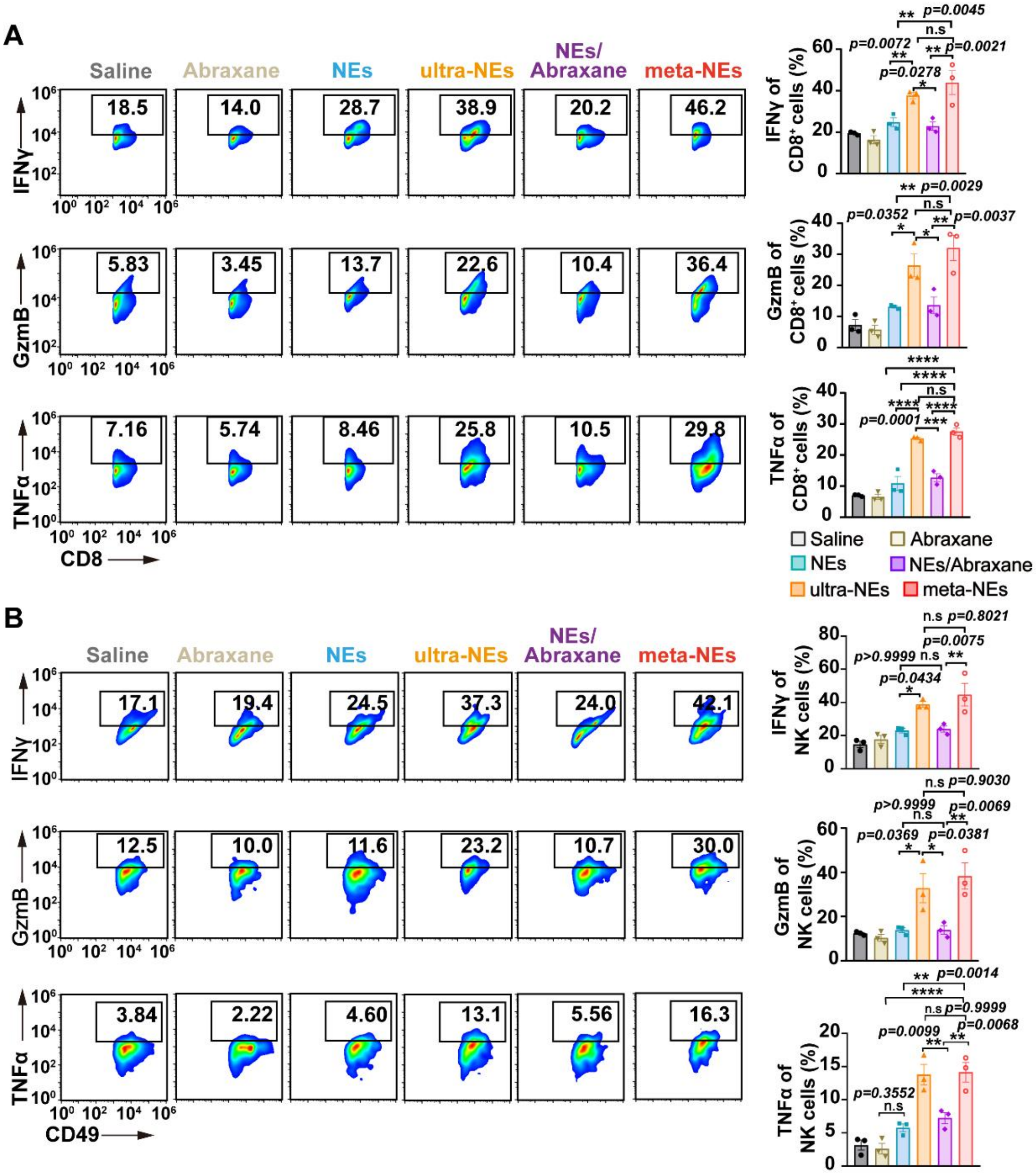

Figure S23. Flow cytometry analysis of IFNγ, GzmB and TNFα levels of tumor-infiltrated CD8^{+}T cell (A) and NK cells (B) in lung metastatic foci from spontaneous TNBC lung metastasis bearing mice. n = 3 mice per group. Related to Figure 6. Data were analyzed by one-way ANOVA test with Tukey's correction and as the mean ± SEM. ****P<0.0001, n.s denotes no significant difference.

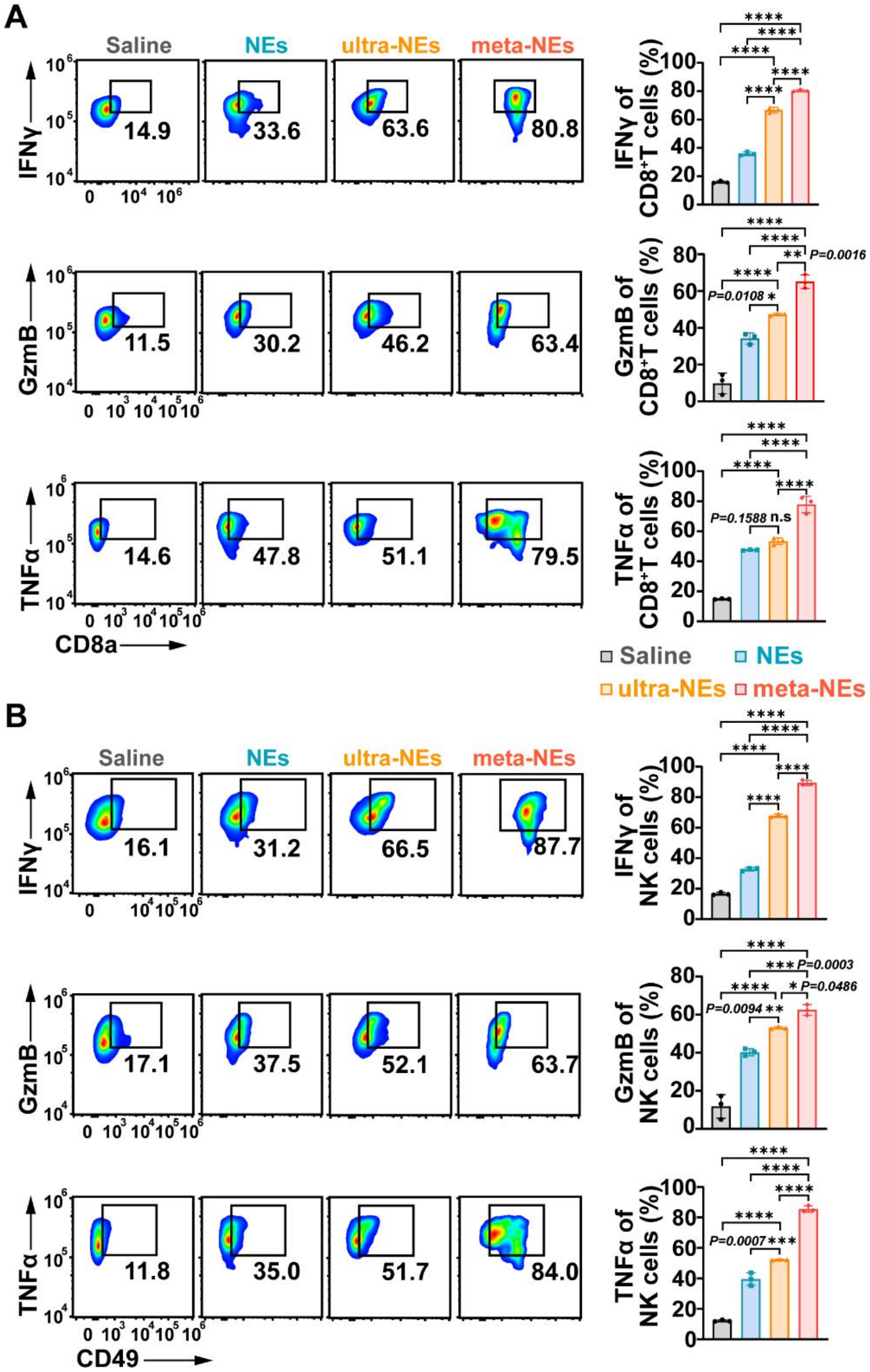

Figure S24. Flow cytometry analysis of IFNγ, GzmB and TNFα levels of tumor-infiltrated CD8$^+$T cell (A) and NK cells (B) in primary tumors from spontaneous TNBC lung metastasis bearing mice. n = 3 mice per group. Related to Figures 6. Data were analyzed by one-way ANOVA test with Tukey's correction and as the mean ± SEM. ****P<0.0001, n.s denotes no significant difference.

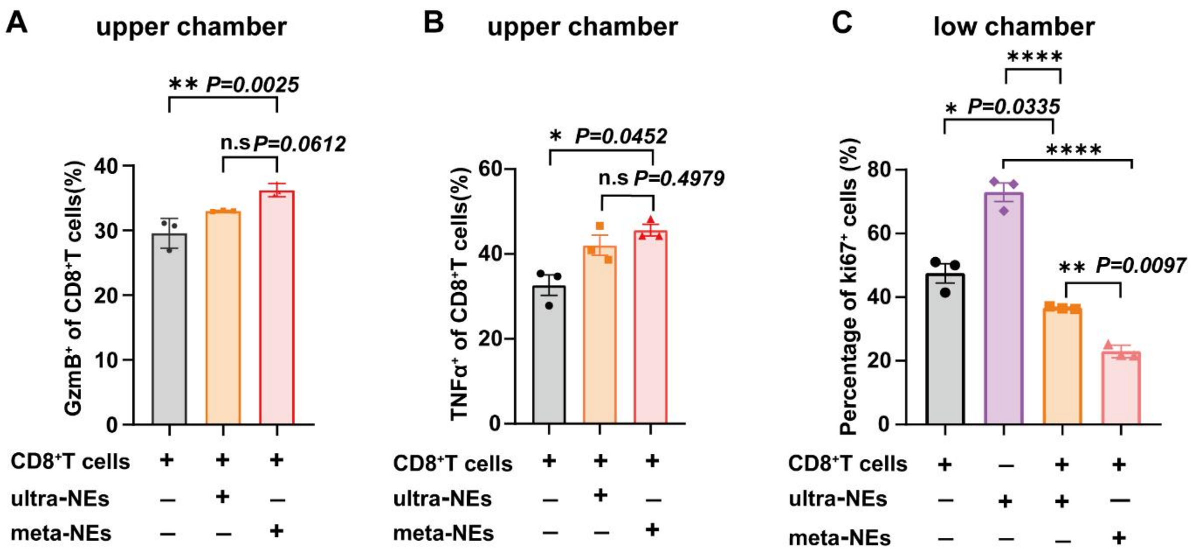

Figure S25. Transwell assay evaluating synergistic effects between NEs-mediated immune activation and Abraxane-mediated cytotoxicity. Expression of GzmB (A) and TNFα (B) in $CD8^{+}T$ cells from the upper chamber. (C) Expression of ki67 in tumor cells from the lower chamber. n = 3 samples per group. Related to Figure 6. Data were analyzed by one-way ANOVA test with Tukey's correction. Error bars denote SEM. $^{****}P < 0.0001$, n.s denotes no significant difference.

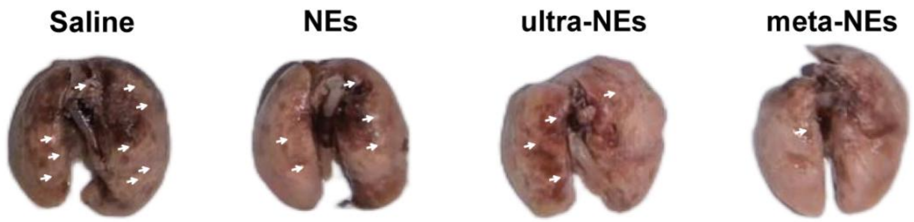

Figure S26. Representative lung metastasis images from a humanized TNBC mouse model. Related to Figure 7.

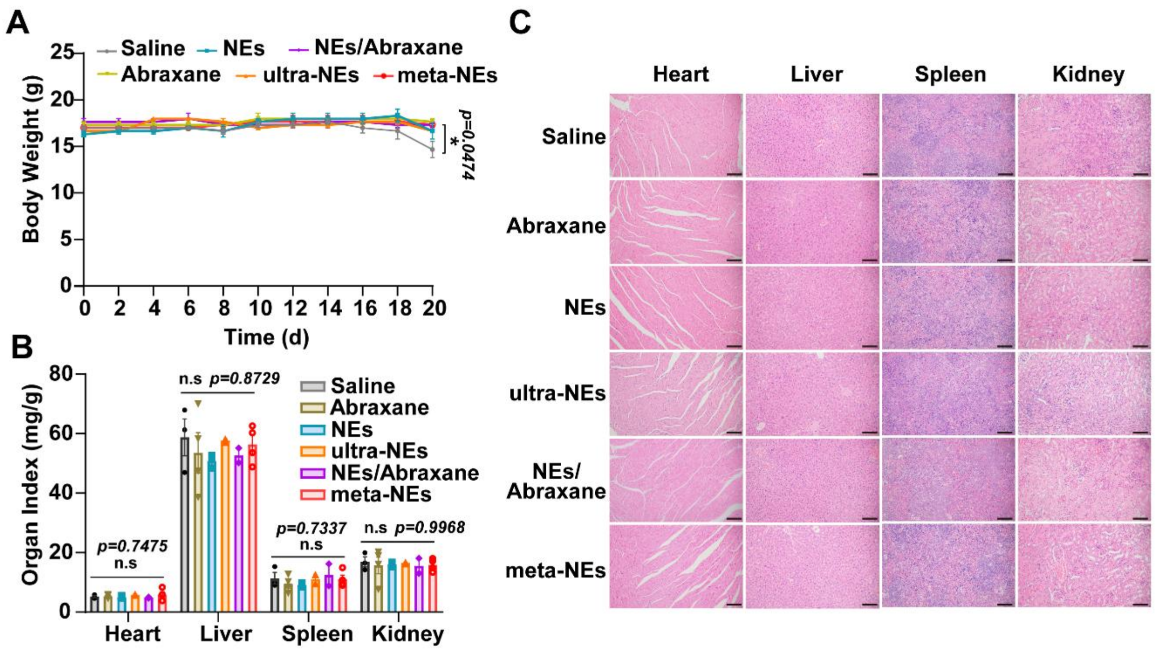

Figure S27. Evaluation of body weight, organ indexes, and H&E changes in established lung metastasis model after administration of meta-NEs. (A) Variations in body weights during the process of treatment (n = 3 mice per group). (B) Organ weight normalized to body weight (n = 4 mice per group). (C) H&E staining of tissue sections of heart, liver, spleen and kidney. Scale bar, 100 μm. Related to Figure 7. Data were analyzed by or two-tailed Student's *t* test (A) or one-way ANOVA test with Tukey's correction (B) and shown as the mean ± SEM. n.s denotes no significant difference.

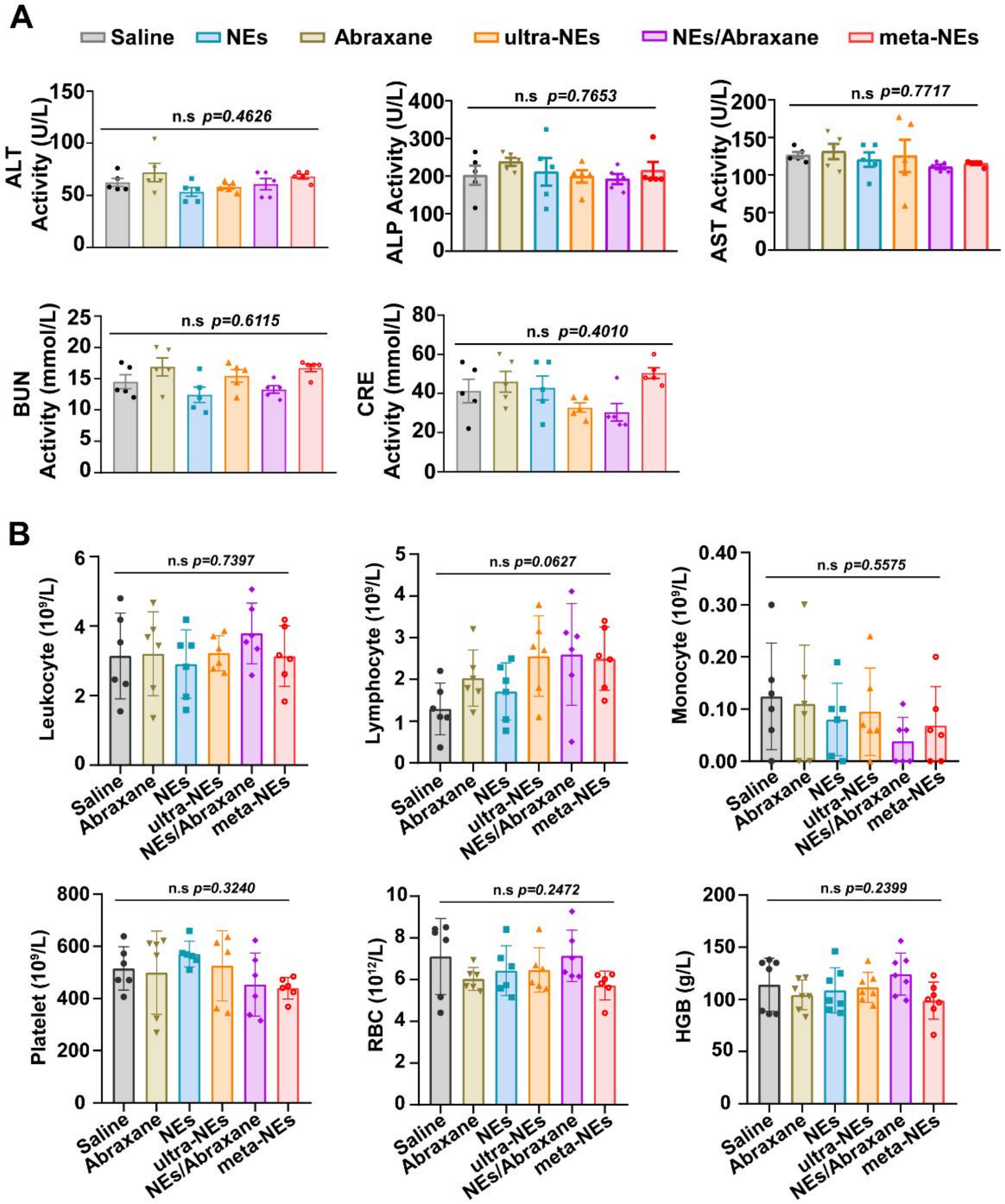

Figure S28. Blood biochemical and hematological analyses in established lung metastasis model after administration of meta-NEs. (A) Expressions of markers of liver function ALT, ALP and AST, as well as kidney function BUN and CRE (n = 5 mice per group). (B) Determination of the numbers of leukocytes, lymphocytes, monocytes, platelets and RBC, as well as the amount of HGB in blood (n = 6 mice per group). Related to Figure 7. Data were analyzed by one-way ANOVA test with Tukey's correction. Error bars denote SEM. ****$P < 0.0001$, n.s denotes no significant difference.

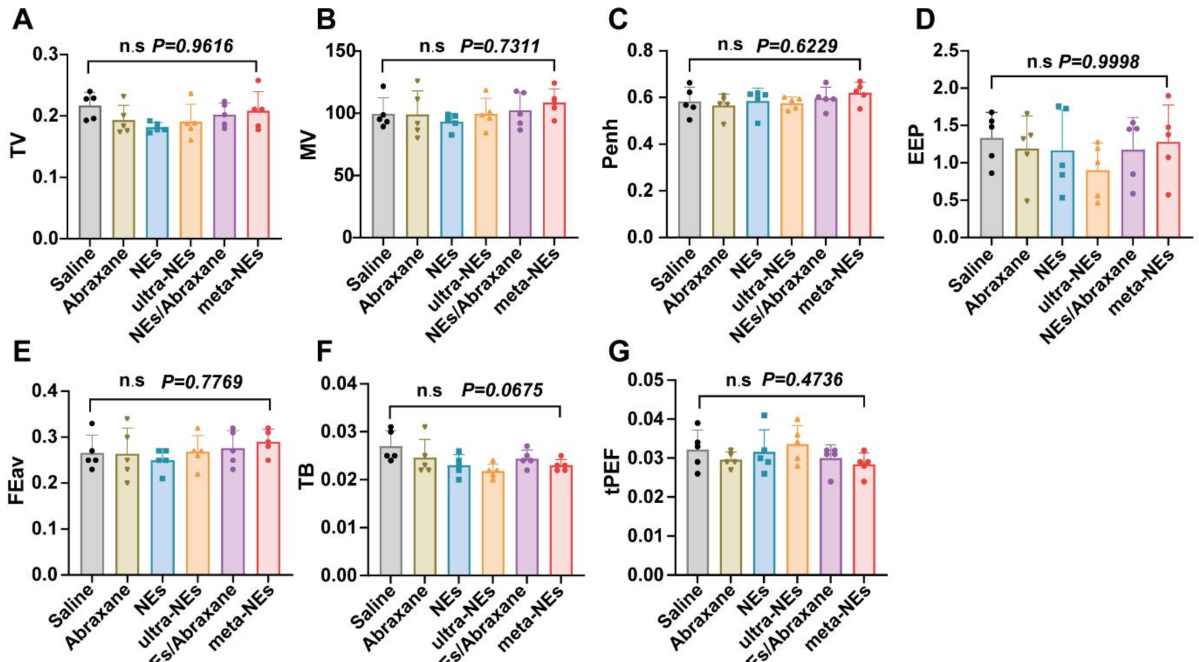

Figure S29. Pulmonary respiratory function assay via whole-body plethysmography. (A) MV-Minute Ventilation. (B) TV-Tidal Volume. (C) Penh-Airway Constriction Index. (D) EEP-End-Expiratory Pause. (E) FEav-Average Expiratory Flow. (F) TB-Airway Inflammation Index. (G) tPEF-Time to Peak Expiratory Flow. n = 5 mice per group. Related to Figure 7. Data were analyzed by one-way ANOVA test with Tukey's correction. Error bars denote SEM.*****P* < 0.0001, n.s denotes no significant difference.

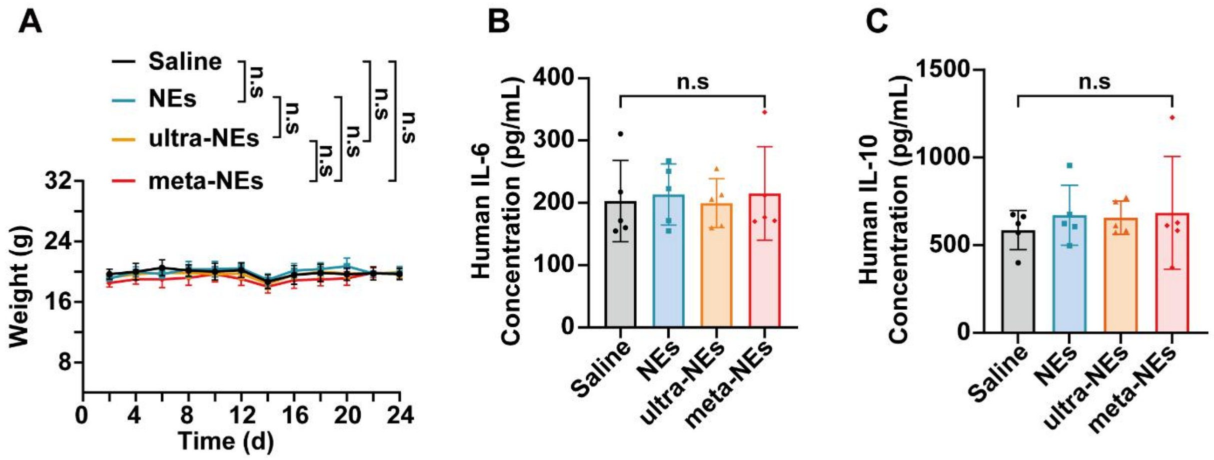

Figure S30. Evaluation of body weight and the risk of a cytokine storm in humanized TNBC mouse model after administration of meta-NEs. (A) Statistics of body weight changes in mice (n = 6 mice per group). (B-C) Levels of human IL-6 and IL-10 in humanized mouse blood after treatment (n = 5 mice per group). Related to Figure 7. Data were analyzed by one-way ANOVA test with Tukey's correction. Error bars denote SEM. n.s denotes no significant difference.

Table S1. Characteristics of the FITC-labeled Abraxane (FITC-Abraxane). PDI, polydispersity index. Related to Figure 3. Data were shown as mean ± SD (n = 3 independent experiments).

	Particle Size (nm)	PDI	Zeta potential (mV)
**Abraxane**	**148.2±1.5**	**0.13±0.07**	**-25.77±0.74**
**FITC-Abraxane**	**160.8±1.0**	**0.13±0.05**	**-21.81±1.71**

Table S2. Primer sequences for real-time PCR. Related to STAR METHODS.

Primers	Forward (5' to 3')	Reverse (5' to 3')
Actb	TGTCCACCTTCCAGCAGATGT	AGCTCAGTAACAGTCCGCCTAGA
Mmp9	TGCCCAGCGACCACAACTC	CGGACCCGAAGCGGACATT
Arg1	GACCACGGGGACCTGGCCTT	ACTGCCAGACTGTGGTCTCCACC
Prok2	TGCTACTTCTGCTGCTACC	CCGCACTGAGAGTCTTGTC
Ccl2	CGGCTGGAGCATCCACGTGTT	TAGCAGCAGGTGAGTGGGGC
Icam1	ACCCACCCCGCAGGTCCAAT	CAGCCGAGGACCATACAGCACG
Tnf	TCGGGGTGATCGGTCCCCAA	GGTGGTTTGCTACGACGTGGG
Trail	GGAAGACCTCAGAAAGTGGCAG	TTTCCGAGAGGACTCCCAGGAT
Ifna	GGATGTGACCTTCCTCAGACTC	ACCTTCTCCTGCGGGAATCCAA
Ifnb1	AAGAGTTACACTGCCTTTGCCATC	CACTGTCTGCTGGTGGAGTTCATC
Cxcl9	CCTAGTGATAAGGAATGCACGATG	CTAGGCAGGTTTGATCTCCGTTC
Cxcl10	AGTGCTGCCGTCATTTTCTG	ATTCTCACTGGCCCGTCAT
