# Supplementary material for: Non-genetically reprogrammed meta-neutrophils potentiate chemo-immunotherapy against lung metastatic triple-negative breast cancer
Source: Cell Rep Med. 2026 Jun 12;7(7):102868. doi: 10.1016/j.xcrm.2026.102868 (PMC13400185; doi:10.1016/j.xcrm.2026.102868)
Supplement: Document S2. Article plus supplemental information [file mmc10.pdf]

Cell Reports Medicine

Article

Non-genetically reprogrammed meta-neutrophils potentiate chemo-immunotherapy against lung metastatic triple-negative breast cancer

Graphical abstract

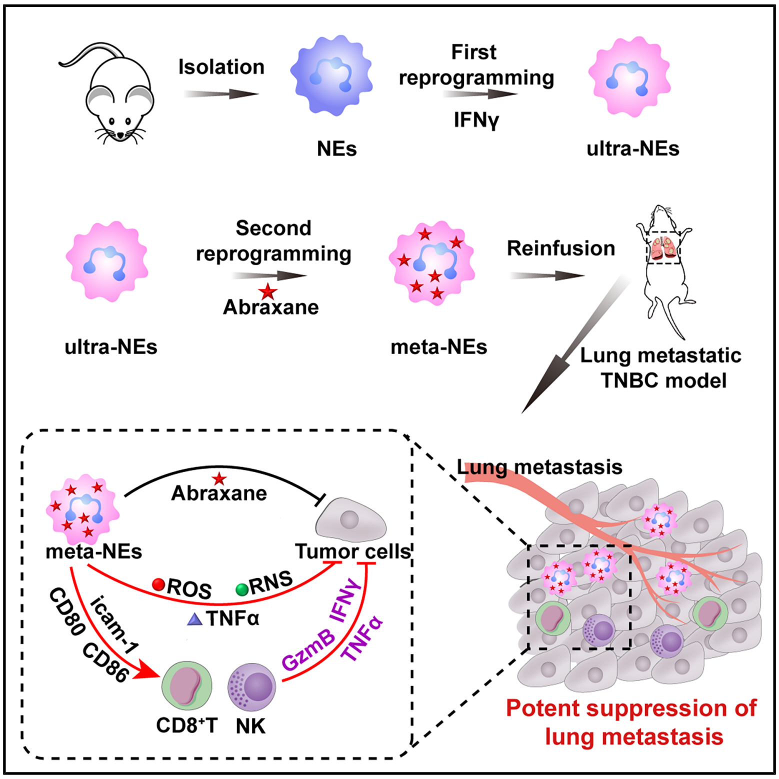

Authors

Meixi Hao, Qifan Hu, Xiuqi Li, ..., Shanshan Chen, Caoyun Ju, Can Zhang

Correspondence

jucaoyun@cpu.edu.cn (C.J.), zhangcan@cpu.edu.cn (C.Z.)

In brief

Hao et al. develop a non-genetic reprogramming strategy using IFNγ training and Abraxane arming to create meta-neutrophils, which sustain antitumor phenotype and enable potent chemo-immunotherapy against lung metastatic triple-negative breast cancer (TNBC), overcoming cell source limitations.

Highlights

- A non-genetic reprogramming strategy is developed to create meta-neutrophils
- Meta-neutrophils maintain antitumor phenotype in immunosuppressive microenvironments
- Meta-neutrophils achieve synergistic chemo-immunotherapy against lung metastatic TNBC
- This strategy applies to healthy and patient-derived NEs, solving cell source limitation

Hao et al., 2026, Cell Reports Medicine 7, 102868
July 21, 2026 © 2026 The Authors. Published by Elsevier Inc.
https://doi.org/10.1016/j.xcrm.2026.102868

Cell Reports Medicine

CellPress
OPEN ACCESS

Article

Non-genetically reprogrammed meta-neutrophils potentiate chemo-immunotherapy against lung metastatic triple-negative breast cancer

Meixi Hao,[1,2] Qifan Hu,[1,2] Xiuqi Li,[1,2] Yijun Chen,[1] Siyuan Hou,[1] Chunjing Han,[1] Sijia Chen,[1] Yanyi Li,[1] Kaiming Li,[1] Lingjing Xue,[1] Lulu Zhu,[1] Shanshan Chen,[1] Caoyun Ju,[1,*] and Can Zhang[1,3,*]

[1]State Key Laboratory of Natural Medicines, Center of Advanced Pharmaceuticals and Biomaterials, China Pharmaceutical University, Nanjing 211198, China

[2]These authors contributed equally

[3]Lead contact

*Correspondence: jucaoyun@cpu.edu.cn (C.J.), zhangcan@cpu.edu.cn (C.Z.)

https://doi.org/10.1016/j.xcrm.2026.102868

SUMMARY

Neutrophils (NEs) exhibit significant therapeutic potential in tumor therapy owing to their distinct advantages. In clinical trials, however, adoptive transfer of NEs shows limited efficacy against advanced solid tumor, partially owing to the unsatisfactory antitumor potency. Besides, the phenotype plasticity of NEs in tumor environment restricts their direct application. Here, we report a non-genetic reprogramming strategy via sequential interferon gamma (IFNγ) training and Abraxane arming to obtain meta-NEs, which hold persisted antitumor phenotype of NEs and potentiated chemo-immunotherapy effect. We find that meta-NEs demonstrate enhanced accumulation in lung metastases and thus harness the strengthened tumoricidal and immunostimulatory potency of NEs and the cytotoxicity of Abraxane to achieve safe and potentiated chemo-immunotherapy against lung metastatic triple-negative breast cancer. In short, we provide a non-genetic reprogramming strategy for NEs to generally improve their antitumor efficacy regardless of donors, which puts forward rationale for the clinical development of NEs in metastatic breast cancer therapy.

INTRODUCTION

Cell therapy is emerging as a developing paradigm in tumor immunotherapy, especially for chimeric antigen receptor (CAR) T cell, which has demonstrated significant progress in hematological malignancies.[1,2] However, CAR T cell therapy faces great challenges in solid tumor treatment owing to the limited infiltration in solid tumors.[3] In contrast, neutrophils (NEs), a type of critical leukocytes in immune systems, have demonstrated efficient tumor infiltration due to their rapid inflammatory chemotaxis toward tumor site and even small metastatic foci,[4,5] as well as effective blood extravasation and tumor penetration.[6] Thus, notable efforts have been dedicated to leverage the tumor tendency of NEs for drug delivery, while ignoring the anti-tumor potency of NEs. A lot of evidences indicate that tumor-infiltrating NEs hold the capability of directly killing cancer cells through the release of toxic reactive oxygen species (ROS), neutrophil extracellular nets (NETs), and so on.[7,8] Notably, they can also stimulate anti-tumor immunity by producing cytokines such as B-cell activating factor (BAFF), CXC-chemokine ligand 8 (CXCL8), and CC-chemokine ligand (CCL20) to recruit and regulate other immune cells like B cells, natural killer (NK) cells, NEs, and T cells.[9–11] These merits make NEs have therapeutic potential in solid tumor immunotherapy.

Despite promise, adoptive NE therapy faces huge challenges due to the cell source. It has been reported that infusion of NEs from healthy donors exhibits limited therapeutic efficacy against advanced solid tumors in phase 1/2 clinical trial (NCT00900497),[12] which can be partially ascribed to the unsatisfactory anti-tumor ability of NEs from healthy donors owing to individual differences. Additionally, autologous NEs, especially from cancerous patients, seem detrimental to tumor therapy due to their pro-tumor risk, although autologous NEs have more biocompatibility.[13] Notable efforts have been dedicated to address the source issue of NEs. For example, the LIfT BioSciences company has found "super donors" whose NEs exhibit exceptional innate cancer killing properties. Based on the hematopoietic stem cells (HSCs) of "super donors," NE progenitor (N-LIfT) cells that can differentiate into immunomodulatory alpha NEs *in vivo* or HER2 CAR-NE through gene engineering HSCs have been developed for solid tumor therapy. However, the limited "super donors," long-term incubation of HSCs *in vitro*, and gene engineering process enhance the economic burden and safety concerns. Most importantly, isolated NEs with short life fail to be directly genetically engineered,[14,15] which increases the difficulty of reprogramming NEs to gain potentiated anti-tumor potency. Alternatively, cytokines have been explored to modulate the immune response of NEs, such as transforming growth factor β inhibitor that can reverse the

 © 2026 The Authors. Published by Elsevier Inc.
This is an open access article under the CC BY license (http://creativecommons.org/licenses/by/4.0/).

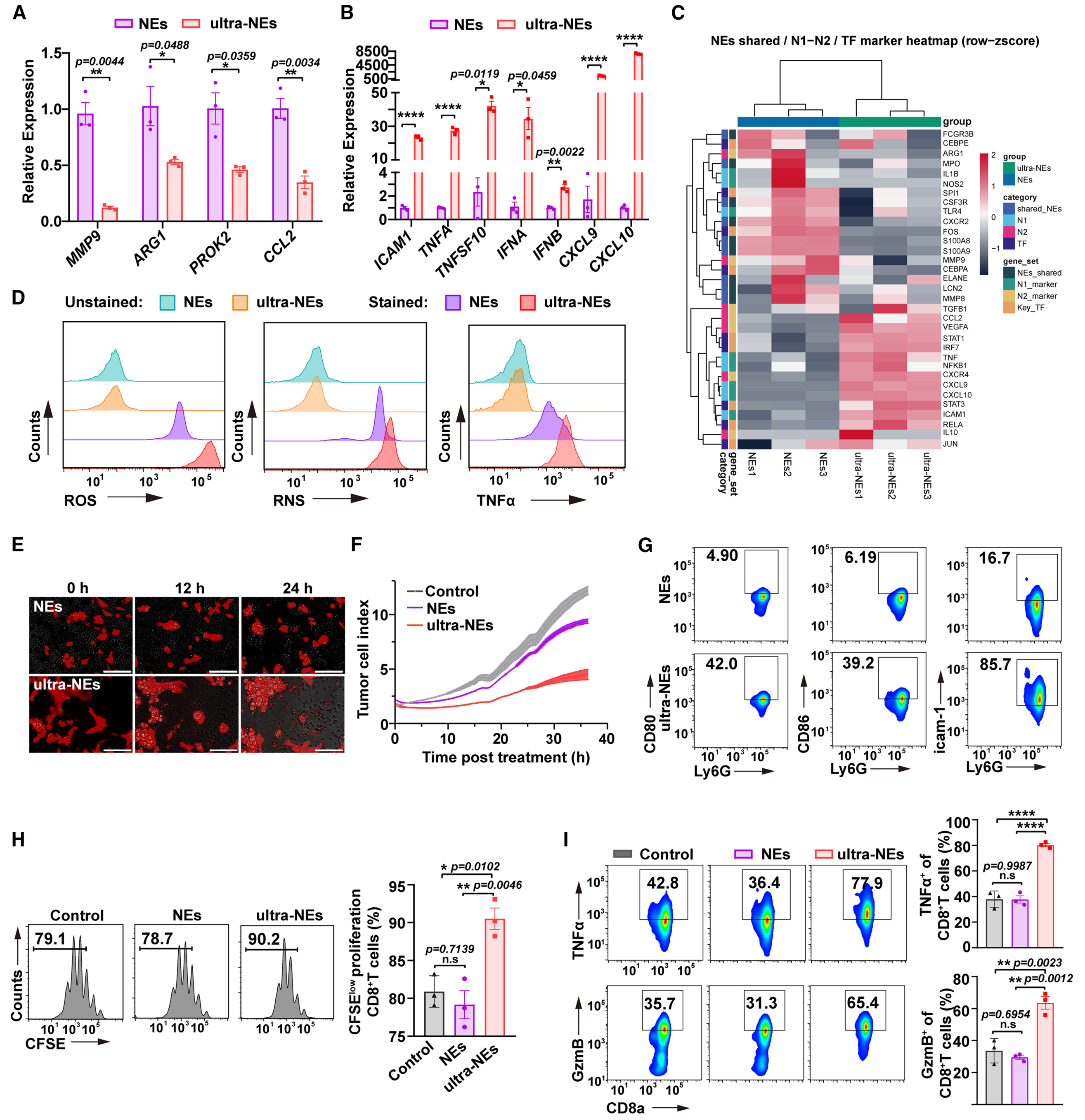

Figure 1. Ultra-NEs strengthen tumoricidal and immunostimulatory potency of NEs from healthy donors

(A and B) Relative mRNA expressions associated with (A) pro-tumor genes and (B) anti-tumor genes of NEs and ultra-NEs isolated from healthy donors.

(C) The marker gene heatmap analysis for NEs and ultra-NEs from healthy donors.

(D) ROS, RNS, and TNFα expressions of NEs and ultra-NEs after stimulation with PMA (100 nM) for 2 h (*n* = 3 samples per group).

(E) Images of mCherry-4T1 cells treated with NEs or ultra-NEs for 12 and 24 h observed by Cytation 1 (*n* = 3 independent samples).

(F) Representative proliferation curves of 4T1 cells after treatment with NEs or ultra-NEs over time as measured by xCELLigence (*n* = 3 independent experiments).

(G) Representative flow cytometry analysis of the expressions of costimulatory molecules CD80, CD86, and icam-1 (*n* = 3 samples per group).

(H) Representative flow cytometry analysis and quantification of the CFSE fluorescence of $CD8^+$T cells cocultured with NEs or ultra-NEs after stimulation by CD3/CD28. $CD8^+$T cells stimulated by CD3/CD28 were used as a control (*n* = 3 samples per group).

(legend continued on next page)

CellPress
OPEN ACCESS

tumor-associated NEs from pro-tumor phenotype to anti-tumor ones.[16–18] Of these endeavors, interferon gamma (IFNγ) has been evidenced to be one of the potent and pleiotropic cytokines in modulating the immune response of NEs.[19–21] Recent evidences suggest that NEs in both human and mouse tumors with high expression of interferon-stimulated genes (ISGs) induced by IFNγ appear essential for successful immunotherapy.[22] More beneficially, IFNγ can delay the programmed cell death and thus extend the lifetime of NEs.[23] However, systemic administration of IFNγ is liable to be rapidly cleared from the blood.[24,25] It is, therefore, essential to frequently re-administer IFNγ to ensure sufficient locoregional concentration to boost NEs, often leading to the systemic toxicity and even playing a pro-tumorigenic role by downregulating major histocompatibility complexes.[25]

To this end, we propose to pre-train NEs via IFNγ to achieve strengthened tumoricidal and immunoregulatory effects of NEs, while avoiding the undesired side effect of systemic administration of IFNγ. Moreover, we would explore whether this strategy can reverse the pro-tumor phenotype of NEs from cancerous patients. Furthermore, we attempt to leverage the IFNγ-trained NEs (ultra-NEs) as the carrier of Abraxane, the Food and Drug Administration (FDA)-approved albumin-bound paclitaxel (PTX) nanoparticles,[26] to arm ultra-NEs with potentiated cytotoxicity for further improving the efficacy of metastatic triple-negative breast cancer (TNBC). The obtained Abraxane-armed ultra-NEs (meta-NEs), which inherit and upgrade the advantages of NEs, can be recruited and accumulated at the lung metastatic foci of TNBC due to their inherent chemotaxis, where they not only release Abraxane to directly kill tumor cells but also utilize ultra-NEs to inhibit tumor growth and promote anti-tumor immunity. In short, we put forward a reprogramming approach for addressing the cell source bottleneck of adoptive NE therapy and provide promising meta-NEs for TNBC treatment, which lays a foundation for clinical transformation in the future.

RESULTS

Ultra-NEs strengthen tumoricidal and immunostimulatory potency of NEs from healthy donors

To understand the IFNγ training effect on NEs, we initially evaluated their nuclear morphology by Wright-Giemsa staining. Our results revealed that ultra-NEs primarily displayed ring-shaped or lobulated nuclei, a feature aligning with the morphology of mature NEs documented in previous studies.[27,28] This outcome suggests that IFNγ training promotes the maturation of NEs (Figure S1). Moreover, we investigated the extent to which IFNγ training prolongs NE survival *in vitro*. Following 12 h of training, the survival rate of ultra-NEs was significantly higher than that of untrained controls. At 24 h, although survival in both groups had declined, over 50% of ultra-NEs remained viable, compared with only approximately 25% in the control group. By 48 h, the survival benefit of IFNγ training, while still statistically significant, dropped below 30%, indicating limited practical utility at this time point (Figure S2). Given the inherently short lifespan of NEs *in vitro*, extending their viability to 24 h may offer a critical window for their use in clinical settings.

Next, we evaluated the anti-tumor potency of ultra-NEs from healthy donors. After IFNγ training, we found the decreased levels of pro-tumor markers including matrix metallopeptidase 9 (*MMP9*), arginase 1 (*ARG1*), prokineticin 2 (*PROK2*), and C-C motif chemokine 2 (*CCL2*) and the increased levels of anti-tumor markers including intercellular adhesion molecule 1 (*ICAM1*), tumor necrosis factor-alpha (*TNFA*), tumor necrosis factor (ligand) superfamily-member 10 (*TNFSF10*), interferon-α (*IFNA*), interferon-β (*IFNB*), C-X-C motif chemokine ligand 9 (*CXCL9*), and C-X-C motif chemokine ligand 10 (*CXCL10*) in ultra-NEs, compared with that of untreated NEs (Figures 1A and 1B).

To further dissect the regulatory effect of IFNγ on the transcriptome of NEs, we performed RNA sequencing analysis on the ultra-NEs and NEs. Principal-component analysis revealed a clear separation between the two groups in the principal component space, indicating that IFNγ activation induced transcriptomic reprogramming (Figure S3A). Hierarchical clustering analysis based on sample correlation further validated the robustness of this effect, with the two groups forming distinct clusters (Figure S3B). The marker gene heatmap showed that IFNγ activation significantly upregulated the expression of pro-inflammatory N1 marker genes and key immune-regulatory transcription factors, while NE signature genes were included to confirm cell identity (Figure 1C). Gene Ontology Molecular Function (GO-MF) enrichment analysis revealed that differentially expressed genes were significantly enriched in molecular functions associated with antigen presentation and immune cell activation, such as "immune receptor activity," "pattern recognition receptor activity," and "MHC class II protein complex binding" (Figure S3C). Gene Ontology Biological Process (GO-BP) enrichment analysis indicated that differentially expressed genes were significantly enriched in biological processes related to pro-inflammatory and anti-tumor activities, including "regulation of immune effector processes," "canonical NF-κB signaling," and "leukocyte-mediated immunity," highlighting the anti-tumor potential of ultra-NEs (Figure S3D). Furthermore, Kyoto Encyclopedia of Genes and Genomes pathway enrichment analysis showed that differentially expressed genes were significantly enriched in pathways related to innate immunity, inflammation-cancer crosstalk, and tumor immunity, including "neutrophil extracellular trap formation," "TNF signaling pathway," and "Th1/Th2 cell differentiation"; these results further support the anti-tumor potential of ultra-NEs (Figure S3E).

In addition, the levels of toxic effector molecules released from ultra-NEs including ROS, reactive nitrogen species (RNS), TNFα, hydrogen peroxide (H_2O_2), and nitric oxide (NO) were significantly upregulated compared with those from NEs, suggesting the strengthened tumoricidal ability of ultra-NEs (Figure 1D; Figures S4A–S4E). To further confirm the anti-tumor potency of

(I) Representative flow cytometry analysis and quantification of TNFα and GzmB of $CD8^+$T cells cocultured with NEs or ultra-NEs after stimulation by CD3/CD28. $CD8^+$T cells stimulated by CD3/CD28 were used as a control (n = 3 samples per group).
Data were analyzed by two-tailed Student's t test (A and B) or one-way ANOVA test with Tukey's correction (H and I). Error bars denote SEM. ****$p < 0.0001$; n.s, no significant difference.

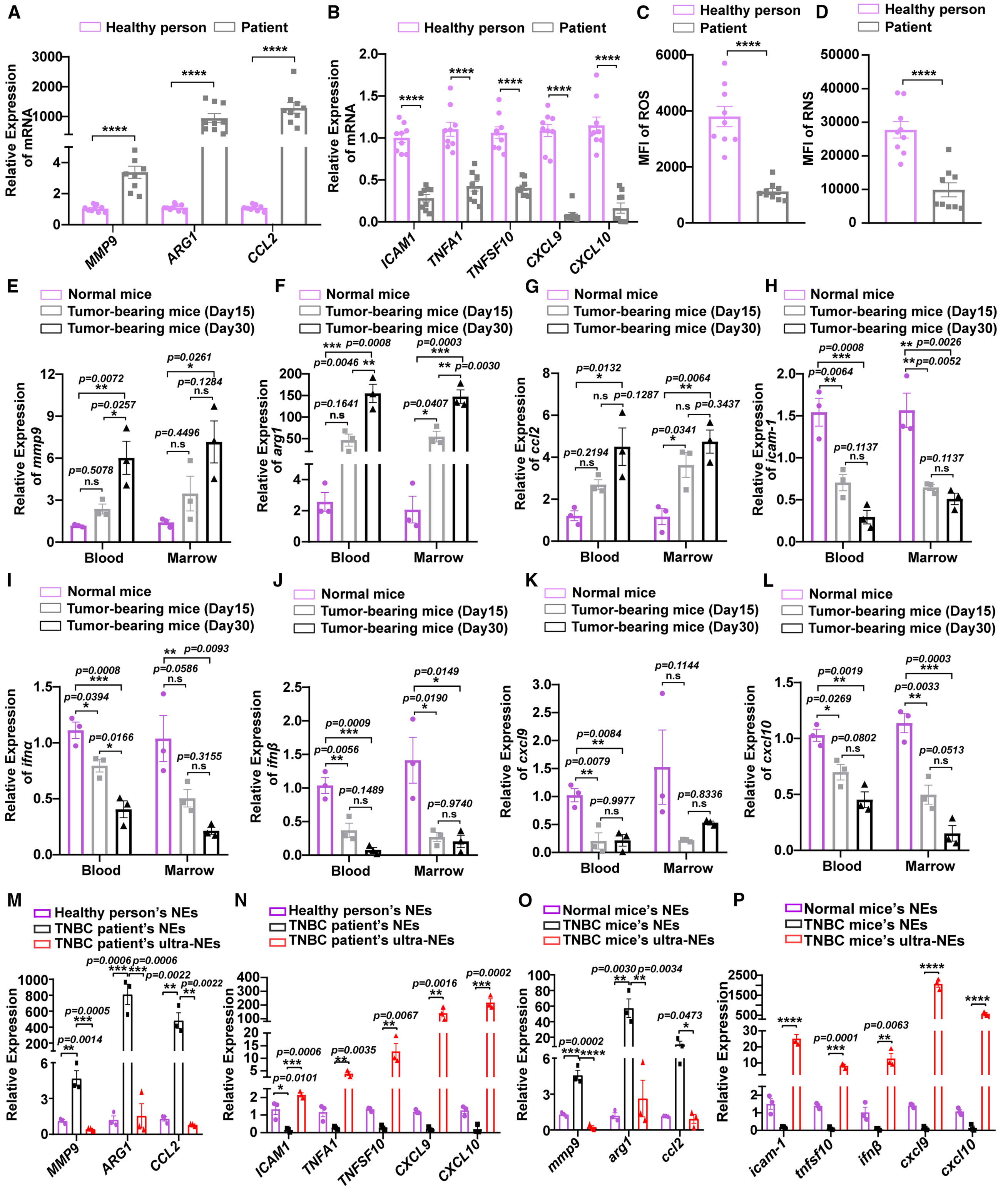

Figure 2. Ultra-NEs reverse the pro-tumor phenotype of NEs from cancerous hosts

(A and B) Relative mRNA expressions associated with (A) pro-tumor genes and (B) anti-tumor genes of NEs in patients suffering from advanced metastatic TNBC (n = 9 independent samples).

(C and D) (C) ROS and (D) RNS expressions of NEs after stimulation with phorbol-12-myristate-13-acetate (PMA, 100 nM) for 2 h.

(legend continued on next page)

ultra-NEs, a direct cytotoxicity assay against mice TNBC 4T1 cells expressing mCherry fluorescence protein (mCherry-4T1) was performed. At an effector cell/target cell ratio of 20:1, ultra-NEs obviously triggered the necrocytosis of tumor cells displaying shrinkage and no intact cell morphology after incubation with ultra-NEs for 12 and 24 h, respectively, while NEs could not effectively kill tumor cells even co-incubating for 24 h (Figure 1E; Videos S1A and S1B), as further confirmed by utilizing a real-time and label-free cellular impedance monitoring station (xCELLigence) (Figure 1F; Figure S4F). Moreover, we compared the tumor killing ability of IFNγ and ultra-NEs and demonstrated that the robust tumor killing ability was ascribed to the anti-tumor potency of ultra-NEs, about 2-fold compared with that of IFNγ (Figure S5).

Interestingly, *CXCL9* and *CXCL10*, the critical mediators of T cell recruitment and activation,[29] exhibited the highest increase in mRNA expressions of ultra-NEs (Figures 1A and 1B). These data implied the potential of ultra-NEs to recruit T cells *in vivo* due to the enhanced expression of *CXCL9* and *CXCL10*. In particular, ultra-NEs had high expression of *CXCL10*, a marker of the ISG response, which is required for successful tumor immunotherapy.[22] Furthermore, we observed that the expressions of costimulatory molecules (CD80 and CD86) and adhesion molecule (icam-1) of ultra-NEs was about 5.9-, 5.6-, and 5.3-fold than that of NEs, respectively (Figure 1G; Figure S6), suggesting the potential of ultra-NEs to immunologically stimulate T cells.[30] Accordingly, we investigated the proliferation and expression of effector molecules of $CD8^{+}$T cells after coculturing with ultra-NEs. The percentages of carboxyfluorescein succinimidyl ester $(CFSE)^{low}$ $CD8^{+}$T cells were 78.7% and 90.2% after co-incubation with NEs or ultra-NEs, respectively (Figure 1H). The expressions of effector molecules such as TNFα and lytic enzyme granzyme B (GzmB) of $CD8^{+}$T cells exhibited similar tendency after ultra-NE treatment (Figure 1I). These results further verified that ultra-NEs held the capability of promoting the proliferation and effector molecule production of $CD8^{+}$T cells, which would be beneficial for boosting the anti-tumor response of $CD8^{+}$T cells.

Taken together, we demonstrated that IFNγ training endowed ultra-NEs with merits of potentiated tumoricidal effect, improved immunostimulatory potency, and prolonged survival, which laid the foundation of future application.

Ultra-NEs reverse the pro-tumor phenotype of NEs from cancerous hosts

Emerging evidences suggest that autologous NEs from cancerous person are tending to be pro-tumor phenotype and with decreased potency, especially in patients suffering from advanced tumors such as lung metastatic breast cancer.[31–33] A similar phenomenon has been found in tumor-bearing mice that NEs derived from bone marrow and blood gradually tend toward a pro-tumor phenotype as tumor progresses. To this end, it is essential to understand whether IFNγ training could reprogram the pro-tumor NEs toward anti-tumor ones. Here, we evaluated the phenotypes of NEs from patients and mice bearing TNBC as compared with those from healthy person and normal mice, respectively. The pro-tumor markers of NEs including *MMP9*, *ARG1*, and *CCL2* showed significantly higher level than that of NEs from healthy person, while the anti-tumor markers such as *ICAM1*, *TNFA*, *TNFSF10*, *CXCL9*, and *CXCL10* were on the contrary (Figures 2A and 2B). These data confirmed that NEs from TNBC patients presented a pro-tumor phenotype as previous reports.[34,35] Furthermore, we also detected the toxic effector molecules secreted from NEs such as ROS and RNS to assess the potency of NEs. As expected, the down-regulated levels of ROS and RNS from NEs of patients compared with that of healthy person indicated the weak tumoricidal effect of NEs from advanced TNBC patients (Figures 2C and 2D). Similarly, NEs from blood or marrow of TNBC-bearing mice showed highly elevated mRNA expressions of pro-tumor genes such as *mmp9*, *arg1*, and *ccl2* and significantly decreased mRNA expressions of anti-tumor genes such as *icam-1*, *ifnβ*, *tnfsf10*, *cxcl9*, and *cxcl10* compared with that of normal mice (Figures 2E–2L). Especially, the variations in phenotypic gene expressions of NEs from TNBC-bearing mice became increasingly apparent as the tumor burden grew (Figures 2E–2L), implying that cancerous patients possessed growing weakened NEs along with tumor progresses.

Next, we explored the phenotype of NEs from TNBC patients after IFNγ training. We found that the levels of pro-tumor genes such as *MMP9*, *ARG1*, and *CCL2* obviously decreased in ultra-NEs compared with NEs (Figure 2M). In contrast, the levels of anti-tumor genes including *ICAM1*, *TNFA*, *TNFSF10*, *CXCL9*, and *CXCL10* significantly increased (Figure 2N), implying that ultra-NEs could circumvent the pro-tumor risk of NEs from TNBC patient. Similar results were found in mice: that the levels of pro-tumor genes (*mmp9*, *arg1*, and *ccl2*) of NEs from tumor-bearing mice downregulated after IFNγ treatment, whereas that of anti-tumor genes (*icam-1*, *tnfsf10*, *ifnβ*, *cxcl9*, and *cxcl10*) upregulated (Figures 2O and 2P).

The antigen-presenting and T cell activation ability of ultra-NEs derived from tumor-bearing mice were also investigated. The results showed that ultra-NEs induced significant upregulation of CD80, CD86, and MHC II expression compared with NEs, confirming that IFNγ-training enhanced the antigen-presenting ability of NEs (Figure S7A). Furthermore, co-incubation of tumor-infiltrating $CD8^{+}$T cells with ultra-NEs enhanced the secretion of IFNγ and TNFα effectors from $CD8^{+}$T cells, confirming the immune-activation effect of ultra-NEs on $CD8^{+}$T cells (Figure S7B). In short, ultra-NEs from cancerous

(E–L) Relative mRNA expression associated with pro-tumor genes including (E) *mmp9*, (F) *arg1*, and (G) *ccl2*, as well as anti-tumor genes including (H) *icam-1*, (I) *ifnα*, (J) *ifnβ*, (K) *cxcl9*, and (L) *cxcl10* of NEs in metastatic TNBC-bearing mice after inoculation for 15 and 30 days, respectively ($n = 3$ mice per group).

(M and N) Relative mRNA expressions associated with (M) pro-tumor genes and (N) anti-tumor genes of NEs and ultra-NEs isolated from advanced TNBC patients ($n = 3$ independent samples).

(O and P) Relative mRNA expressions associated with (O) pro-tumor genes and (P) anti-tumor genes of NEs and ultra-NEs isolated from TNBC mice ($n = 3$ mice per group). NEs from healthy person or normal mice were set as respective controls.

All data were shown as the mean ± SEM. Data were analyzed by one-way ANOVA test with Tukey's correction. ****$p < 0.0001$; n.s, no significant difference.

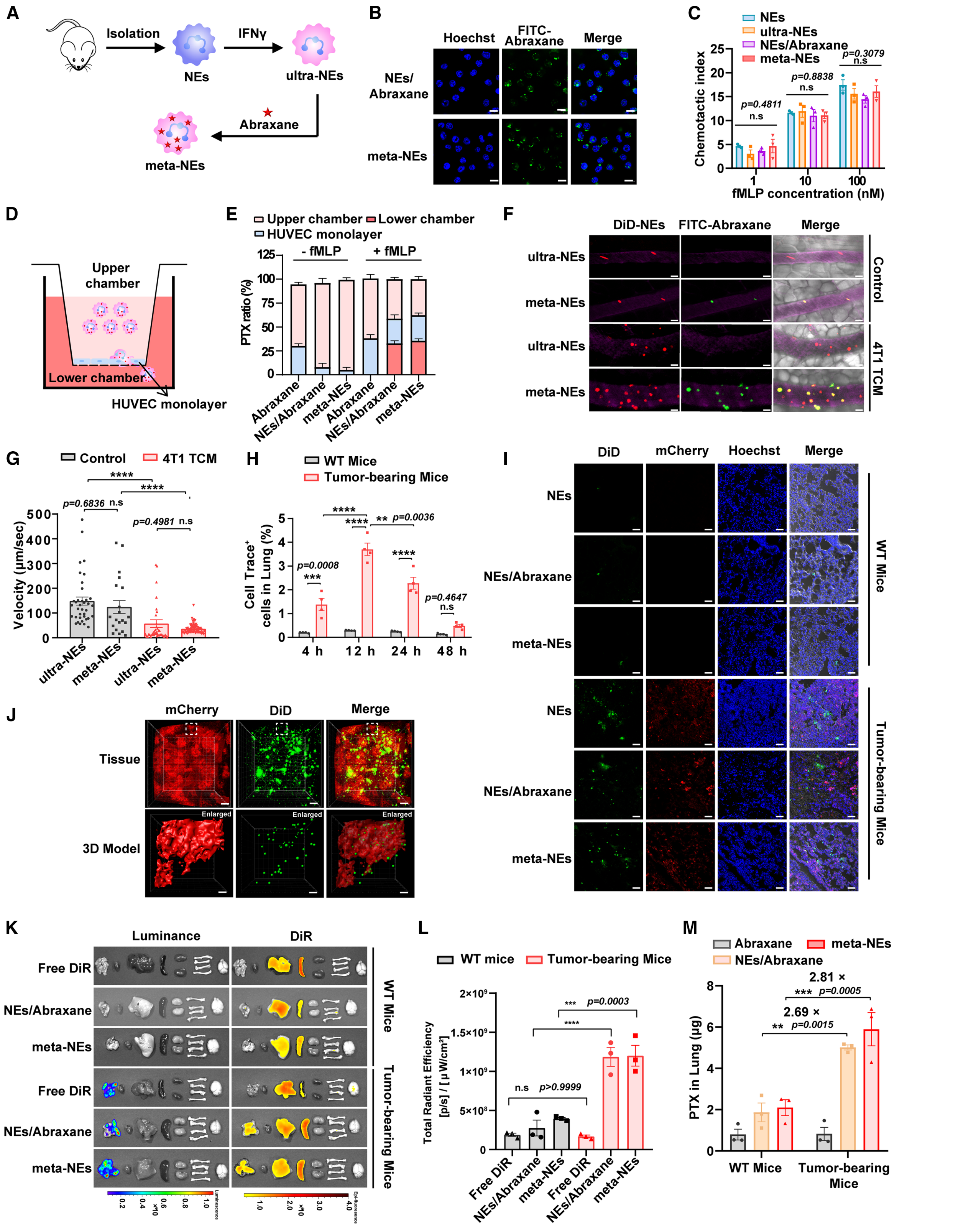

(legend on next page)

hosts avoided the pro-tumor risk of NEs, suggesting the potential of utilizing autogenous NEs of cancerous patients in clinic.

Preparation and pulmonary metastasis accumulation of meta-NEs

To improve the therapeutic effect of reprogrammed ultra-NEs, we proposed to further arm ultra-NEs by using cytotoxic drugs. For this purpose, we produced meta-NEs by incubating albumin-bound PTX nanoparticles (brand name: Abraxane) with ultra-NEs (Figure 3A) at the optimized feeding concentration and incubation time (Figure S8), which showed a drug loading efficiency of PTX in meta-NEs of around 4.5 μg per million NEs. Moreover, the green fluorescence of fluorescein isothiocyanate (FITC)-labeled Abraxane (FITC-Abraxane), which held a similar particle size and zeta potential as Abraxane (Table S1), uniformly dispersed in the cytoplasm of ultra-NEs (Figure 3B), as confirmed by the z stack view (Video S2), verifying that Abraxane had been successfully located within ultra-NEs. Moreover, Abraxane demonstrated intracellular stability under both storage-related physiological conditions (phosphate-buffered saline [PBS] or 50% serum) (Figure S9A), an attribute that may be explained by its non-lysosomal pathway of cellular uptake (Figure S9B). In a tumor-mimicking environment (4T1 tumor cell-conditioned medium [4T1 TCM]), Abraxane was subject to rapid release (Figure S9C), which would favor the tumor killing when arriving at the tumor site.

The physiological functions including viability, chemotaxis, and transendothelial ability determine the targeting efficiency of meta-NEs to tumor site. We confirmed that meta-NEs held similar cell viability (Figure S10), inflammation chemotaxis (Figure 3C), and transendothelial ability (Figures 3D and 3E) as NEs, indicating the preserved physiological functions. Of note, on administration of N-formyl-methionyl-leucyl-phenylalanine (fMLP) as a chemoattractant, about 35% of PTX was detected in the lower chamber in the meta-NE group, while less than 1% PTX was determined in the lower chamber in all three groups without fMLP and the group of Abraxane with fMLP (Figure 3E). It suggested that free Abraxane could hardly cross the monolayer even in the presence of fMLP due to the tight junctions between neighboring endothelial cells, whereas NEs and meta-NEs under inflammatory condition had the capability of transporting across the vessel endothelium along with the encapsulated Abraxane. Additionally, meta-NEs maintained the naive ability of NEs to release NETs (Figure S11), which would favor the cargo release in the tumor environment.

Next, we evaluated the *in vivo* chemotaxis of meta-NEs to the lung metastases. 4T1 breast cancer cell TCM-mediated chemotaxis model was established by subcutaneously injecting 4T1 TCM in the abdomen to simulate the tumor microenvironment, followed by the intravenously administering 1,1′-dioctadecyl-3,3,3′,3′-tetramethylindodicarbocyanine perchlorate (DiD)-labeled ultra-NEs carrying FITC-Abraxane. The movement of DiD and FITC-labeled meta-NEs toward the 4T1 TCM was observed in local veins using two-photon microscopy (Figure 3F; Video S3). Only scattered infused NEs could be found in the absence of 4T1 TCM (Videos S3A and S3B), whereas the number of infused NEs increased in the view after the injection of 4T1 TCM (Videos S3C and S3D). Besides, we observed the significantly reduced flow rate of the infused ultra-NEs and meta-NEs in the simulated tumor environment compared with the normal condition (Figure 3G). Meanwhile, part of the infused NEs rolled or adhered on the blood vessel wall induced by the 4T1 TCM, and during the progress, the red fluorescence of DiD-labeled ultra-NEs and the green fluorescence of FITC-Abraxane mostly overlapped. All these data suggested that meta-NEs in the circulation were recruited by the inflammatory signal released from the tumor site, following which they slowed down and crossed the vessel wall to arrive at the tumor site, causing Abraxane to remain within the cells.

Figure 3. Preparation and pulmonary metastasis accumulation of meta-NEs

(A) Scheme illustration of meta-NEs preparation.
(B) Confocal images of NEs loaded with Abraxane (NEs/Abraxane) and meta-NEs. Abraxane was labeled with FITC. Scale bars, 5 μm (n = 3 independent samples).
(C) Chemotaxis index of meta-NEs under different fMLP concentrations (n = 3 samples per group).
(D) Diagram of the Transwell for the transendothelial ability assay of meta-NEs.
(E) Quantification of PTX amount in the upper chamber, HUVEC layer, and lower chamber with or without 10 nM fMLP using the Transwell model shown in (D) (n = 3 samples per group).
(F) Representative image of meta-NEs in local veins *in situ*, near the site where 4T1 TCM was injected to simulate the tumor microenvironment. NEs were labeled with DiD. Abraxane was labeled with FITC. Scale bars, 20 μm (n = 3 independent samples).
(G) Flow rate of meta-NEs in local veins calculated by the data in (F). Data were calculated with Imaris. n = 37 for IFNγ-NEs in control group. n = 20 for meta-NEs in control group. n = 41 for IFNγ-NEs in 4T1 TCM group. n = 80 for meta-NEs in 4T1 TCM group.
(H) Frequency of CellTrace-labeled meta-NEs in lung tissues of metastasis-bearing mice and WT mice over time (n = 4 mice per group).
(I) Confocal images of lung frozen sections from mCherry-4T1-bearing mice after administration of DiD-labeled meta-NEs for 12 h. Nuclei were stained with Hoechst 33342. Scale bars, 50 μm.
(J) Whole-lung images of mCherry-4T1-bearing mice after treating with DiD-labeled meta-NEs for 12 h by using tissue optical clearing technique combined with two-photon microscopy. Representative region was selected and 3D modeled to illustrate the location of meta-NEs (green) and tumor cells (red). Scale bars: 200 μm (top) and 30 μm (bottom).
(K) *Ex vivo* imaging of tissues harvested from WT mice and 4T1-Luci-bearing mice after intravenous injection of different DiR formulations for 12 h.
(L) Quantitation of DiR fluorescence in lungs with metastases.
(M) Amounts of PTX in lung harvested from WT mice and 4T1-Luci-bearing mice after intravenous injection of different Abraxane formulations for 12 h (n = 3 mice per group).
Data were analyzed by one-way ANOVA test with Tukey's correction (C, G, and L) or two-way ANOVA test with Tukey's correction (H and M). Error bars denote SEM. ****p < 0.0001; n.s, no significant difference.

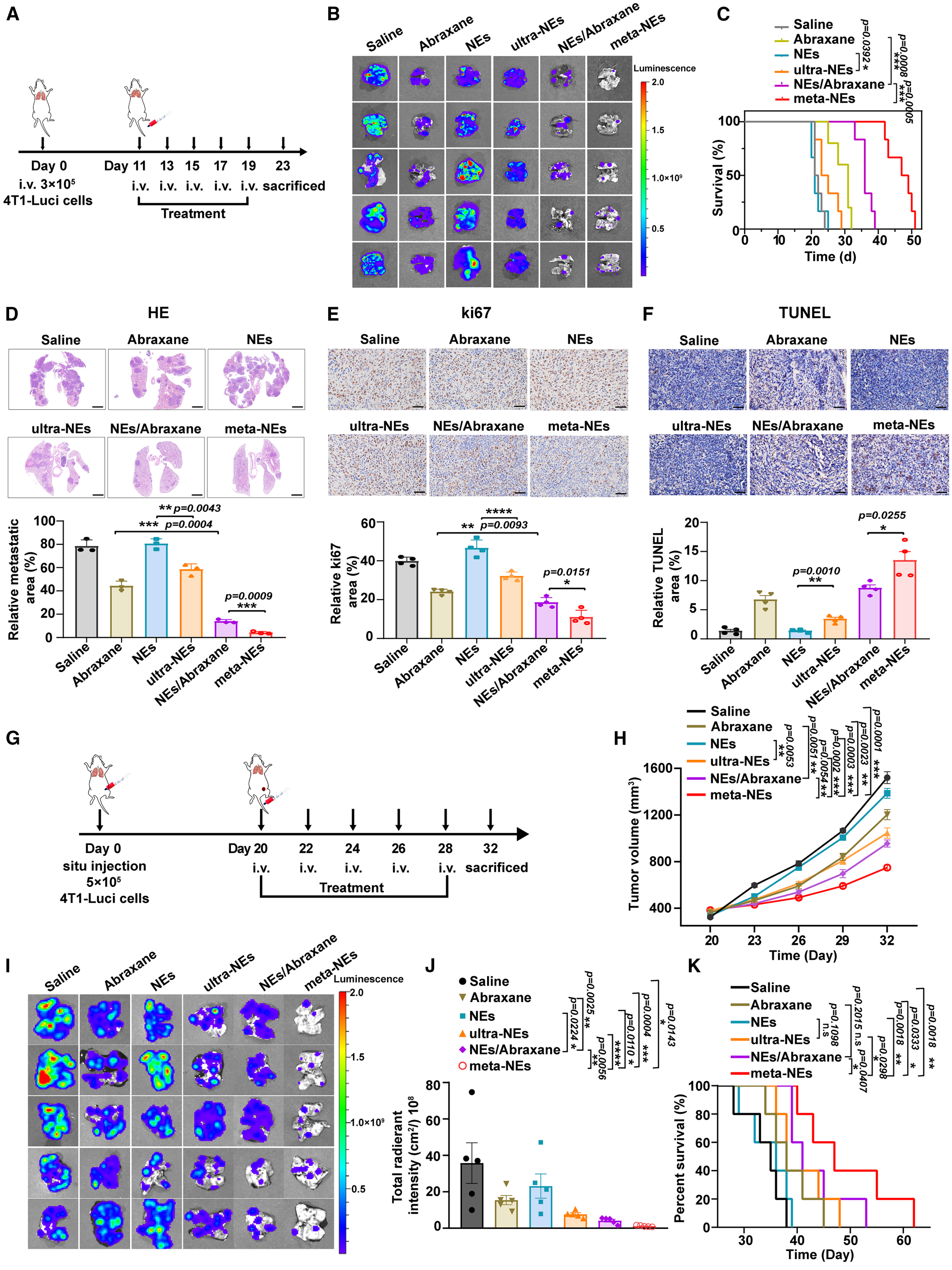

(legend on next page)

To further verify the specific accumulation of meta-NEs in lung metastases, the lung metastases-bearing mice were obtained by intravenous injection of 4T1 cells for 10 days. After that, CellTrace Far Red-labeled meta-NEs were reinfused to investigate the spontaneous migration to lung metastatic foci by analyzing the frequency of CellTrace$^+$ cells at lungs over time. The result displayed that CellTrace$^+$ cells accumulated in lungs in a time-dependent manner and plateaued at 12 h post-administration (Figure 3H). To understand the distribution of meta-NEs in tumor site, we assessed the frozen section of lung tissues with mCherry-4T1 metastases at 12 h post-administration of DiD-labeled meta-NEs. The DiD signals of meta-NEs surrounded the mCherry signals of 4T1 cells, indicating that meta-NEs could successfully accumulate at the lung metastatic foci, whereas the lung section of wild-type (WT) mice showed limited DiD signals (Figure 3I; Figure S12A). The images of whole lung with metastases by using tissue optical clearing technique combined with two-photon microscopy indicated that meta-NEs deeply penetrated to the lungs with metastases (Figure 3J; Video S4), which would be beneficial for the following tumoricidal effect.

To further confirm the accumulation of meta-NEs in lung metastases, tissue distribution assay was performed in TNBC lung metastases-bearing mice, which were obtained by intravenous injection of luciferase-expressing 4T1 cells (4T1-Luci cells). After administration of 1,1′-dioctadecyl-3,3,3′,3′-tetramethylindotricarbocyanine iodide (DiR)-labeled meta-NEs for 12 h, major organs were harvested and observed by using the *ex vivo* imaging system (Figures 3K and 3L). Meta-NEs exhibited remarkably enhanced DiR signals at lung tissues in tumor-bearing mice compared with that in WT mice, implying a specific metastases-targeting ability of the meta-NEs. Moreover, the PTX amount in metastatic lung tissues was about 2.81-fold higher than that in normal lung tissues (Figure 3M; Figure S12B), owing to the effective targeting ability of meta-NEs. Of note, meta-NEs showed comparable metastases-targeting efficiency with NEs, which further demonstrated that the two-step non-genetic engineering had no influence on the chemotaxis of NEs.

Together, we had successfully fabricated meta-NEs that retained the chemotaxis ability of NEs to target and penetrate to the lung metastatic foci, by which they could improve the accumulation and uniform distribution of ultra-NEs and cytotoxic drugs in lung metastases.

Meta-NEs elicit robust inhibition in TNBC lung metastases

Encouraged by the tumoricidal and immunostimulatory potency of ultra-NEs, as well as the ability of meta-NEs to target lung metastases, we proceeded to investigate the suppressive effect of meta-NEs on a mouse model of TNBC lung metastasis. As shown in Figure 4A, a mouse model of lung metastasis was established by intravenous injection 4T1-Luci cells. On day 11 post-inoculation, the mice were randomly divided into six groups and separately received intravenous injections of saline, Abraxane, NEs, ultra-NEs, NEs loaded with Abraxane (NEs/Abraxane), and meta-NEs every 2 days for 5 times. Mice were sacrificed at day 23, and the lung metastases were monitored via monitoring the bioluminescence of 4T1-Luci cells (Figure 4B; Figure S13). We found that mice receiving meta-NEs displayed the fewest lung metastases among all the groups. Of the groups, the NE group exhibited nearly no suppression of lung metastases compared with the saline group, while ultra-NEs showed modest anti-metastasis effect. The result indicated that NEs lacked sufficient anti-tumor effect, whereas ultra-NEs could strengthen the anti-tumor potency due to the IFNγ reprogramming. Combining the strengthened potency of ultra-NEs and the increased accumulation of Abraxane in lung metastases, meta-NEs possessed the most potent anti-metastasis capability of about 97.4%, leading to the longest median survival of 48 days, which was about 2.3-fold of saline (21 days), 1.5-fold of Abraxane (31 days), and 1.3-fold of NEs/Abraxane (36 days) (Figure 4C). Moreover, the hematoxylin and eosin (H&E) staining of lungs harvested from mice receiving different formulations verified the superior anti-metastasis capacity of meta-NEs due to the smallest amount and size of lung metastases (Figure 4D). Consistently, the minimum expression of ki67 (a proliferation marker) and the highest apoptosis area by immunohistochemical staining of terminal deoxynucleotidyl transferase dUTP nick end labeling (TUNEL) in the lungs of meta-NE-treated mice confirmed the significant inhibition on lung metastases (Figures 4E and 4F). Therefore, we believed that meta-NEs held great potential to be robust therapeutic agents against TNBC lung metastases.

As well known, patients with advanced TNBCs are prone to lung metastasis.[36] Thus, spontaneous lung metastases model was constructed by injection of 4T1-Luci cells into the right mammary fat pad of female BALB/c mice for 20 days, until the

Figure 4. Antitumor efficacy of meta-NEs in lung metastasis models

(A) Schematic illustration of the experimental design for intravenous injections of lung metastasis models.

(B) Representative bioluminescence images of lungs harvested from the tumor-bearing mice receiving tested formulations (n = 5 mice per group).

(C) Survival curves of the tumor-bearing mice receiving tested formulations (n = 6 mice per group).

(D) Representative H&E-stained images and quantification of metastatic area in lung sections harvested from mice receiving tested formulations (n = 3 mice per group). Scale bars, 2 mm.

(E) Representative images and quantification of lung sections immunostained by ki67 (n = 4 mice per group). Scale bar, 100 μm.

(F) Representative images and quantification of lung sections immunostained by TUNEL (n = 4 mice per group). Scale bars, 100 μm.

(G) Schematic illustration of the experimental design for spontaneous lung metastasis models.

(H) Primary tumor growth curves (n = 5 mice per group).

(I) Bioluminescence images of lungs harvested from the spontaneous TNBC lung metastasis-bearing mice receiving tested formulations (n = 5 mice per group).

(J) Total radiant efficiency of lung bioluminescence in (I).

(K) Survival curves (n = 5 mice per group).

Data were analyzed by log rank (Mantel-Cox) test (C and K) or one-way ANOVA test with Tukey's correction (D–F, H, and J). Error bars denote SEM. ****$p < 0.0001$; n.s, no significant difference.

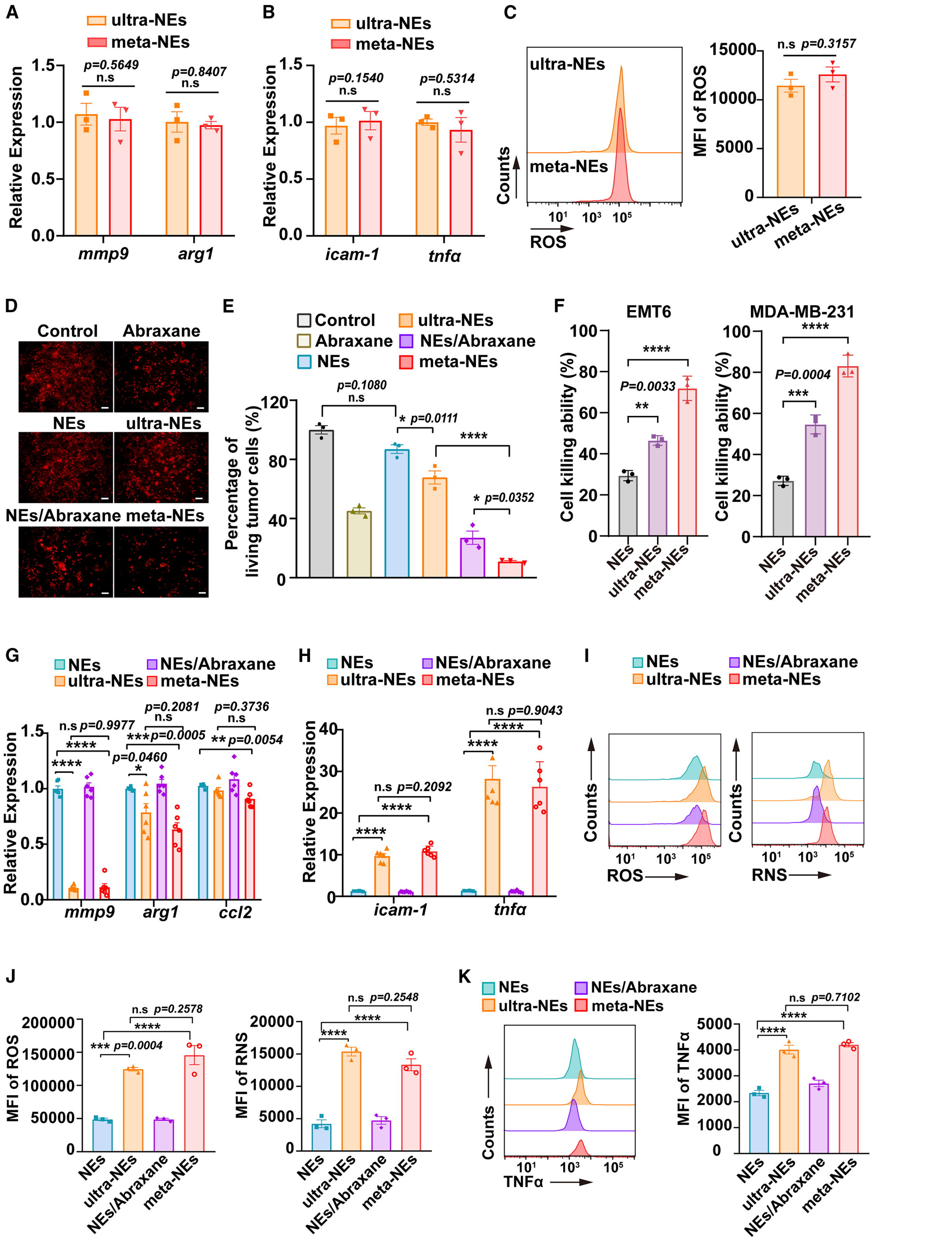

(legend on next page)

presence of lung metastatic lesions was observed by the bioluminescence of 4T1-Luci cells (Figure S14). Before exploring the therapeutic effect of meta-NEs, we first evaluated the biodistribution of meta-NEs to understand whether meta-NEs could traffic to the primary tumor and metastases. As expected, meta-NEs showed similar biodistribution with NEs, which could accumulate at the primary tumor and lung metastatic foci due to the chemotaxis of NEs, and peaked at 12 h for lung metastatic foci and 48 h for primary tumor after injection, respectively (Figures S15A–S15C). The effective targeting of meta-NEs resulted in the higher accumulation of PTX in primary tumor and lung metastases, which displayed 3- and 5-fold higher PTX concentrations than that in the Abraxane group at 12 or 48 h, respectively (Figure S15D). The high accumulation of meta-NEs in both primary tumor and lung metastases guaranteed the potentiated therapeutic effect.

To evaluate the efficacy, these tumor-bearing mice were randomly divided into six groups and received the first administration at day 20 after inoculation (Figure 4G). The volume of primary tumors was monitored, displaying that meta-NEs significantly inhibited the tumor growth than other groups over 32 days (Figure 4H). After that, mice were sacrificed and lung tissues were isolated for detection of the metastatic foci by using the *ex vivo* imaging system. We found that mice receiving meta-NEs possessed the fewest metastases among all the groups (Figures 4I and 4J). Moreover, 50% of mice receiving meta-NEs survived for at least 48 days, compared with saline-treated mice survival of about 33 days (Figure 4K). Together, these results further proved the effectiveness of meta-NEs against advanced spontaneous TNBC lung metastasis.

The tumoricidal potency of meta-NEs

We hypothesized that the superior antitumor capacities of meta-NEs are partially attributable to the antitumor phenotype conferred by IFNγ. To verify this hypothesis, we first wondered whether Abraxane arming would reshape the phenotype of meta-NEs. As shown in Figures 5A–5C, meta-NEs displayed comparable levels of pro-tumor markers of *mmp9* and *arg1* and anti-tumor markers of *icam-1* and *tnfα*, as well as the released ROS, with ultra-NEs. To further validate the tumoricidal potency of meta-NEs, we treated mCherry-4T1 cells and observed that they exhibited the weakest fluorescence intensity (Figures 5D and 5E), indicating the potent anti-tumor efficacy of meta-NEs. This enhanced killing effect was not limited to 4T1 cells; meta-NEs also demonstrated robust cytotoxicity against two additional TNBC cell lines (EMT6 and MDA-MB-231), consistently outperforming ultra-NEs (Figure 5F). This superior activity may be attributed to the combined direct cytotoxicity of the Abraxane released from meta-NEs and the toxic effector molecules secreted from ultra-NEs.

To further understand whether meta-NEs possessed the tumoricidal potency in the tumor environment where NEs are liable to convert to the pro-tumor ones, we assayed the roles of reinfused meta-NEs in lung metastatic foci. For convenient assay, DiD-labeled formulations including NEs, NEs/Abraxane, ultra-NEs, and meta-NEs were infused into 4T1 lung metastasis-bearing mice. As Ly6G was the specific and representative marker for NEs, the isolated $Ly6G^{+}DiD^{+}$ cells from the lungs harvested from the mice with a purity above 90% were considered as the reinfused meta-NEs (Figure S16). Next, we analyzed relative gene expressions of these isolated cells. The levels of pro-tumor genes of *mmp9*, *arg1*, and *ccl2* decreased about 4.2-, 1.6-, and 1.1-fold in meta-NE-treated mice compared with that in NE group, respectively (Figure 5G). By contrast, the expressions of anti-tumor genes of *icam-1* and *tnfα* increased about 8.6- and 19.6-fold in mice receiving meta-NEs than that in NE-treated mice, respectively (Figure 5H). Moreover, these genes of meta-NEs showed similar levels as those of ultra-NEs. These data suggested that meta-NEs in the tumor site maintained the anti-tumor phenotype.

We also measured the levels of toxic effector molecules, including ROS, RNS, and TNFα, to elucidate the antitumor efficacy of micron-scale nanocarriers within metastatic lesions. As expected, in pulmonary metastatic nodules, reinfused ultra-NEs and meta-NEs elicited significantly higher levels of ROS, RNS, and TNFα compared with their unprimed counterparts (i.e., NEs and NEs/Abraxane) (Figures 5I–5K). These findings indicate that the secretion of cytotoxic mediators is conferred by IFNγ priming, and that meta-NEs retain this enhanced effector function even within the metastatic microenvironment. Consistent results were obtained in pulmonary metastases from spontaneous TNBC lung metastasis mouse model (Figure S17), where ultra-NEs and meta-NEs also induced elevated secretion of ROS, RNS, and TNFα. This further confirms that the enhanced release of cytotoxic effectors is attributable to IFNγ preactivation and importantly, that this effect is preserved in advanced spontaneous metastatic settings.

Figure 5. The tumoricidal potency of meta-NEs
(A) Relative mRNA expressions of pro-tumor genes in meta-NEs (n = 3 samples per group).
(B) Relative mRNA expressions of anti-tumor genes in meta-NEs (n = 3 samples per group).
(C) ROS level of meta-NEs after stimulating with 100 nM PMA for 2 h (n = 3 samples per group).
(D) Representative fluorescence images of anti-tumor effect of meta-NEs against mCherry-4T1 cells at an effector cell: target cell ratio of 20:1. Images were recorded at 12 h post-incubation. Untreated mCherry-4T1 cells were used as controls. Scale bars, 100 μm (n = 3 independent samples).
(E) Percentage of living cells after incubation with meta-NEs, which was calculated by the fluorescence intensity of (D).
(F) Evaluating the cytotoxicity of meta-NEs against EMT6 and MDA-MB-231 (n = 3 samples per group).
(G) Pro-tumor gene expression of reinfused NEs in lung metastatic foci (n = 6 mice per group).
(H) Anti-tumor gene expressions of reinfused NEs in lung metastatic foci (n = 6 mice per group).
(I) Flow cytometry analysis of ROS and RNS expressions of reinfused NEs in lung metastatic foci (n = 3 mice per group).
(J) Quantification of ROS and RNS expression.
(K) Flow cytometry analysis and quantification of TNFα expression.
Data were analyzed by two-tailed Student's t test (A, B, and C) or one-way ANOVA test with Tukey's correction (E–H and J–K). Error bars denote SEM. $^{****}p < 0.0001$; n.s, no significant difference.

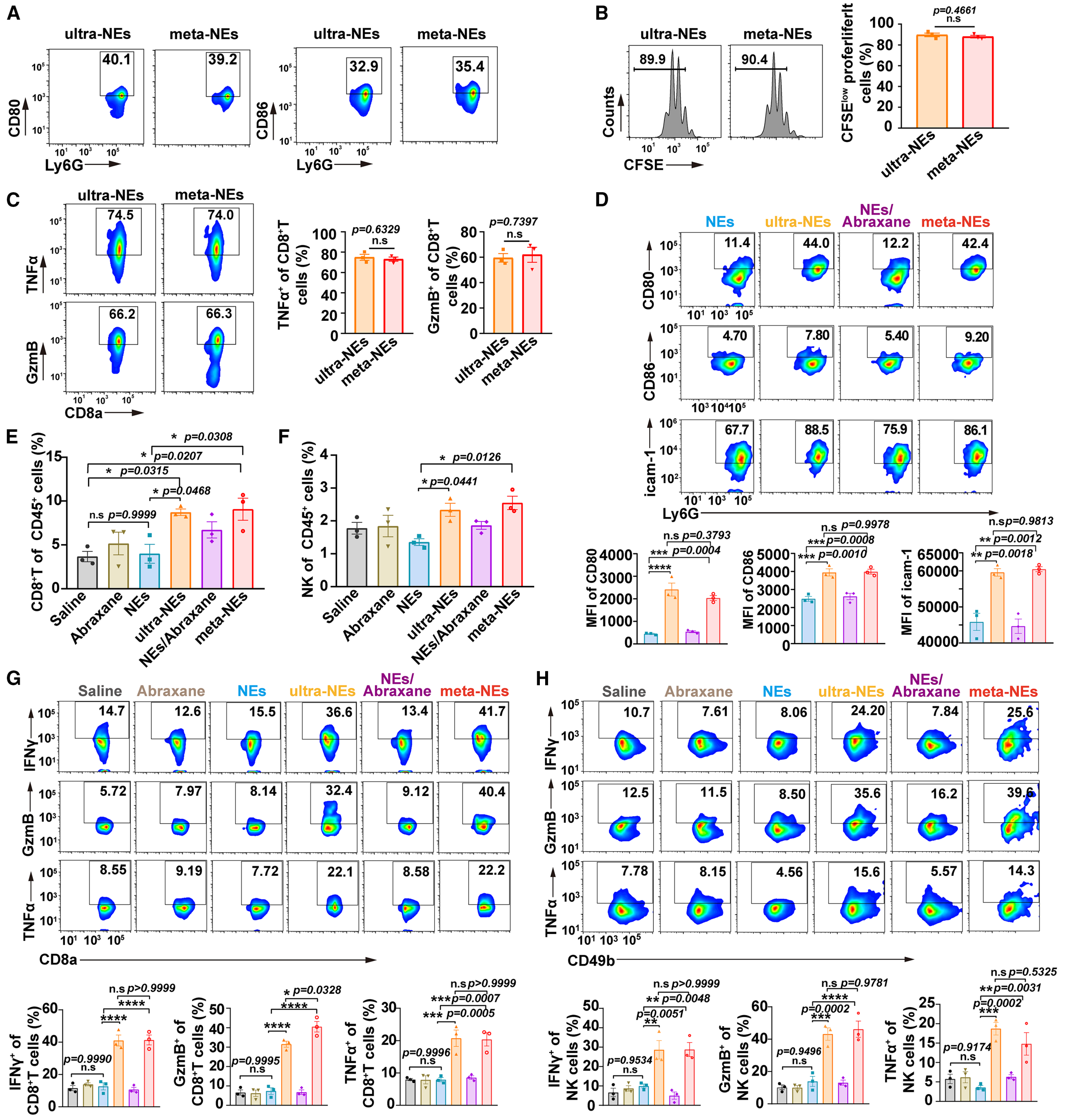

Figure 6. The immunoregulatory potency of meta-NEs

(A) CD80 and CD86 expressions of meta-NEs (n = 3 samples per group).

(B) Representative flow cytometry analysis and quantification of CFSE fluorescence of $CD8^+$T cells co-cultured with ultra-NEs or meta-NEs stimulated by CD3/CD28 (n = 3 samples per group).

(C) Representative flow cytometry images and quantification of TNFα and GzmB expressions of $CD8^+$T cells co-cultured with ultra-NEs or meta-NEs stimulated by CD3/CD28 (n = 3 samples per group).

(D) Flow cytometry analysis and quantification of CD80, CD86, and icam-1 in reinfused NEs in lung metastatic foci (n = 3 mice per group).

(E) Relative infiltration percentage of $CD8^+$T cells in metastatic foci (n = 3 mice per group).

(F) Relative infiltration percentage of NK cells in metastatic foci (n = 3 mice per group).

(legend continued on next page)

Collectively, these results demonstrate that the antitumor phenotype of meta-NEs is sustained within lung metastases, which is crucial for their ability to inhibit metastatic progression.

The immunoregulatory potency of meta-NEs

Given the previously demonstrated immunomodulatory effects of ultra-NEs on CD8$^+$T cells, we hypothesized that the potent antitumor activity of meta-NEs may be partly attributed to their regulatory effects on CD8$^+$T cells. To test this hypothesis, we first evaluated the immunomodulatory potency of meta-NEs on CD8$^+$T cells, including their CD80 and CD86, as well as their ability to promote CD8$^+$T cell proliferation and enhance effector molecule production. The results showed that meta-NEs exhibited no significant differences from ultra-NEs in terms of CD80 and CD86 expression, or in their capacity to promote CD8$^+$T cell proliferation and effector cytokine secretion (Figures 6A–6C; Figure S18). These findings indicate that meta-NEs retain the immunomodulatory functions conferred by IFNγ treated.

Further, we assayed the frequency of reinfused cells with expressions of CD80 and CD86 and adhesion molecule (icam-1) in lung metastases. Higher population and expression levels of CD80$^+$, CD86$^+$, and icam-1$^+$ NEs cells were found in lungs harvested from mice receiving meta-NEs and ultra-NEs than those from mice receiving NEs and NEs/Abraxane (Figure 6D), which indicated the persistent immunoregulatory effect of ultra-NEs and meta-NEs. For further proof of this, we measured the relative infiltration percentage of immune cells in lung metastatic foci. The number of tumor-infiltrated CD8$^+$T cells and NK cells improved 2.5- and 1.4-fold after treatment with meta-NEs than that in mice treated with saline and increased 2.3- and 1.9-fold compared with NE groups, respectively (Figures 6E and 6F). However, the tumor-infiltrated macrophages and DCs showed no significant difference in all treatment groups (Figure S19). Similar results were obtained in pulmonary metastases from a spontaneous TNBC lung metastasis mouse model (Figure S20). Therefore, we concluded that meta-NEs mainly modulated the immune microenvironment by recruiting CD8$^+$T cells and NK cells rather than macrophages and DCs.

We further employed enzyme-linked immunosorbent assay (ELISA) to quantitatively assess the levels of IFNγ and GzmB secreted in both tumor tissues and peripheral blood of mice from different treatment groups. The results are detailed in Figure S21. Within tumor tissues, the meta-NE group exhibited a marked increase in IFNγ concentration, reaching approximately 10-fold that of the saline group. Moreover, GzmB levels were significantly elevated in both the ultra-NE and meta-NE groups. These results suggest that meta-NE treatment promotes the secretion of anti-tumor effector molecules by tumor-infiltrating immune cells, such as T cells and NK cells. In peripheral blood, no statistically significant differences in IFNγ or GzmB concentrations were observed among any of the treatment groups, indicating that the anti-tumor effector molecule secretion and cytotoxic effects were largely confined to the local tumor site, rather than eliciting a systemic response.

To deeply understand the role of meta-NEs in modulating CD8$^+$T cells and NK cells, the anti-tumor responses of CD8$^+$T cells and NK cells in lung metastases were evaluated. We found that the tumor-infiltrated CD8$^+$T cells experienced a significant up-regulation of the production of IFNγ, GzmB, and TNFα in mice receiving meta-NEs, which indicated a boosted activation of CD8$^+$T cells with an expected tumor-killing capacity (Figure 6G). Similar enhanced potency of NK cells was achieved in meta-NE group due to their potentiated expressions of effector molecules including IFNγ, GzmB, and TNFα (Figure 6H). It was consistent with the previous reports, proving that meta-NEs could enhance the tumoricidal function of NK cells.[37,38] Last, we detected the CD80 and CD86 expressions in tumor-infiltrated DCs (Figure S22). There were no significant differences among all the tested groups. These results indicated that meta-NEs held the immunoregulatory capability to directly boost the activation of CD8$^+$T cells and NK cells rather than promoting the maturation of DCs. Consistent with this, in the spontaneous TNBC lung metastasis model, meta-NE treatment also led to significantly elevated expression of IFNγ, GzmB, and TNFα in tumor-infiltrating CD8$^+$T cells and NK cells (Figure S23), further demonstrating that meta-NEs can remodel the immune microenvironment in metastatic lesions by potentiating the activation of CD8$^+$T cells and NK cells within the lung. Moreover, similar results were performed on primary tumors. In mice receiving meta-NEs, tumor-infiltrating CD8$^+$T cells and NK cells significantly upregulated the production of IFNγ, GzmB, and TNFα, indicating enhanced activation and tumor-killing capacity (Figure S24).

To investigate the synergistic interplay between Abraxane-mediated cytotoxicity and NE-induced immune activation, we systematically evaluated their temporal synergy using a Transwell co-culture system. In this setup, distinct immune cell subsets were seeded in the upper chamber, while tumor cells were placed in the lower chamber. Assessment of GzmB and TNFα expression in CD8$^+$T cells from the upper chamber revealed significantly increased positivity rates for both markers in groups supplemented with either ultra-NEs or meta-NEs compared with the control, indicating rapid immune cell activation (Figures S25A and S25B). Concurrently, tumor cells from the lower chamber were evaluated for ki67 positivity as a measure of proliferation inhibition. The group co-cultured with both CD8$^+$T cells and ultra-NEs in the upper chamber exhibited a significantly lower ki67 positivity rate compared with the group with CD8$^+$T cells alone, suggesting that ultra-NEs exert indirect anti-tumor effects via CD8$^+$T cell activation. Furthermore, the group with both CD8$^+$T cells and meta-NEs in the upper chamber displayed a markedly reduced ki67 positivity rate compared with the group with CD8$^+$T cells and ultra-NEs, indicating that Abraxane released by meta-NEs synergizes with their immune-activating capacity to enhance anti-tumor efficacy. Notably,

(G) Flow cytometry analysis and quantification of IFNγ, GzmB, and TNFα levels of tumor-infiltrated CD8$^+$T cell in lung metastatic foci (n = 3 mice per group).
(H) Flow cytometry analysis and quantification of IFNγ, GzmB, and TNFα levels of tumor-infiltrated NK cells in lung metastatic foci (n = 3 mice per group).
Data were analyzed by two-tailed Student's t test (B and C) or one-way ANOVA test with Tukey's correction (D–H). Error bars denote SEM. ****$p < 0.0001$; n.s, no significant difference.

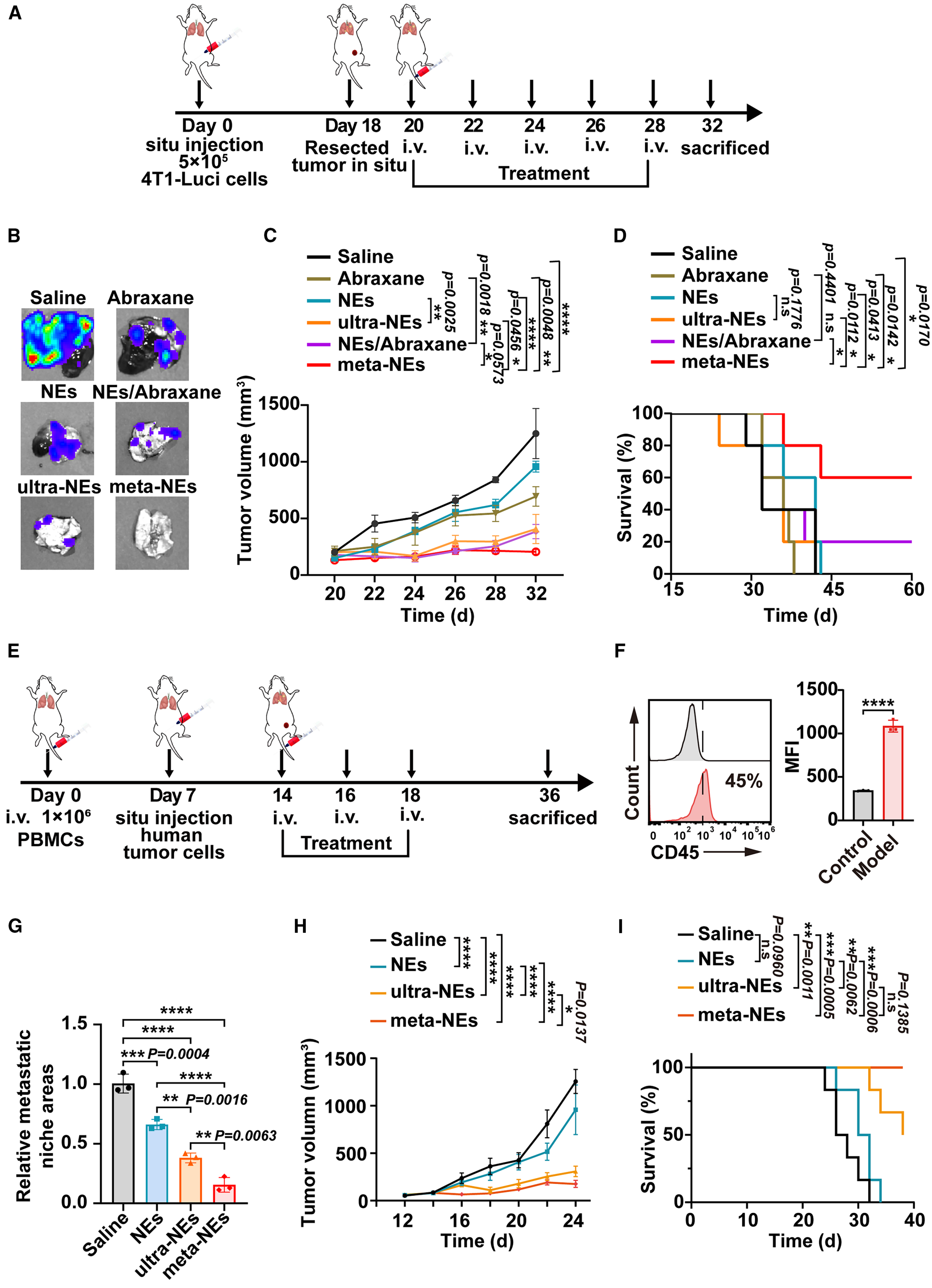

(legend on next page)

the group with ultra-NEs alone in the upper chamber exhibited the weakest anti-tumor activity, suggesting that the anti-tumor effects of ultra-NEs primarily rely on immunomodulation of $CD8^+$T cells, whereas their direct tumoricidal effect—mainly mediated by antibody-dependent cellular cytotoxicity[39]—is limited without direct tumor cell contact (Figure S25C). Collectively, these findings demonstrate that, within the observed time frame, NE-induced immune activation and Abraxane-mediated cytotoxicity occur in a temporally synergistic manner, implying that both mechanisms jointly contribute to the augmentation of anti-tumor immune responses.

Overall, we confirmed that the non-genetic reprogramming strategy significantly enhances the immunoregulatory potency of NEs within the tumor environment. This, combined with the IFNγ-primed tumoricidal phenotype of the NEs themselves and the direct cytotoxic action of the conjugated Abraxane, constitutes a three-pronged approach that underpins the superior chemo-immunotherapeutic efficacy of meta-NEs against lung metastatic TNBC.

The clinical application potential of meta-NEs

In order to better fit the clinical application, the superior therapeutical efficacy of meta-NEs was further confirmed in a TNBC spontaneous lung metastasis model with *in situ* resection of primary tumor (Figure 7A). Mice treated with meta-NEs exhibited the strongest anti-tumor efficacy, according to the markedly reduced metastatic foci (Figure 7B), delayed postoperative recurrence *in situ* (Figure 7C), as well as the significantly prolonged survival (Figure 7D). Promisingly, three of five mice receiving meta-NEs survived to 60 days, while mice from other groups survived no more than 49 days. In short, these findings indicated that meta-NEs showed great potential in inhibiting the postoperative recurrence and metastasis of TNBC.

To further align with clinical practice, a humanized TNBC mouse model was established (Figure 7E), and successful model construction was confirmed by the proportion of $CD45^+$ cells in peripheral blood exceeding 45% (Figure 7F). The results demonstrated that meta-NEs not only effectively inhibited lung metastases (Figure 7G; Figure S26) but also exerted a potent therapeutic effect on orthotopic tumors, achieving a tumor inhibition rate of 83% (Figure 7H). Moreover, mice treated with meta-NEs exhibited significantly prolonged survival, with no deaths observed within 40 days, whereas all mice in the saline group died by day 31 post-tumor inoculation (Figure 7I). These findings further validate the robust antitumor efficacy of meta-NEs.

Safety of meta-NEs

To evaluate the *in vivo* safety of meta-NEs, we first utilized a lung metastasis model established by intravenous injection of 4T1 cells. None of the treatment caused significant loss of body weights except the saline group at day 20 due to the devastating growth of lung metastases (Figure S27A). Moreover, no tissue damage was found in each group through the assessment of organ indexes and H&E-stained histologic sections of heart, liver, spleen, and kidney (Figures S27B and S27C). Specifically, treatment with meta-NEs did not induce any significant changes in the expressions of markers of liver injury including alanine aminotransferase, alkaline phosphatase, and aspartate aminotransferase, as well as kidney injury including blood urea nitrogen and creatinine (Figure S28A). To investigate whether meta-NEs induce lung injury, we employed whole-body plethysmography to perform a multidimensional assessment of pulmonary function in mice. The results showed that, compared with the saline, mice receiving meta-NEs exhibited no significant differences in the following respiratory parameters: tidal volume ($p = 0.9616$), an indicator of single-breath efficiency; minute volume ($p = 0.7311$), reflecting overall ventilation capacity; enhanced pause ($p = 0.6229$), a measure of airway resistance and expiratory difficulty; end-expiratory pause ($p = 0.9998$), representing the proportion of expiratory time; average expiratory flow ($p = 0.7769$); peak expiratory flow ($p = 0.0675$); and time to peak expiratory flow ($p = 0.4736$) (Figure S29). Collectively, these findings indicate that the meta-NE treatment used in this study did not impair acute ventilatory function, increase airway resistance, or reduce respiratory efficiency in mice, suggesting that it does not cause significant lung tissue injury. Moreover, the number of leukocytes, lymphocytes, monocytes, platelets, and red blood cells and the amount of hemoglobin also showed no noticeable difference among all the groups (Figure S28B). These results, therefore, indicated the good safety of meta-NEs.

Additionally, in the humanized TNBC mouse model, no significant abnormalities in body weight or serum cytokine levels were observed in the meta-NE treatment group and no notable adverse reactions were detected, further confirming its favorable safety profile and potential for clinical application (Figure S30).

DISCUSSION

Cell therapy has attracted significant attention in tumor therapy due to its natural biological activities, such as active tendency to target site, cytotoxic capability, and so on.[40,41] Of these types

Figure 7. Therapeutic efficacy of meta-NEs against both primary tumors and metastases in composite TNBC models featuring spontaneous lung metastasis post-resection and humanized mice

(A) Schematic illustration of the experimental design in spontaneous TNBC lung metastasis model.

(B) Representative bioluminescence images of lungs in spontaneous TNBC lung metastasis model ($n = 5$ mice per group).

(C) Tumor growth curves in spontaneous TNBC lung metastasis model ($n = 5$ mice per group).

(D) Survival curves ($n = 5$ mice per group).

(E) Schematic illustration of the experimental design in humanized mouse model.

(F) CD45 expression levels in humanized mouse model ($n = 3$ mice per group).

(G) Relative metastatic niche areas in humanized mouse model ($n = 3$ mice per group).

(H) Primary tumor growth curves in humanized mouse model ($n = 6$ mice per group).

(I) Survival curves in humanized mouse model ($n = 6$ mice per group).

Data were analyzed by one-way ANOVA test with Tukey's correction (C, G, and H), two-tailed Student's *t* test (F), or log rank (Mantel-Cox) test (D and I). Error bars denote SEM. $^*p < 0.05$, $^{**}p < 0.01$, $^{****}p < 0.0001$; n.s, no significant difference.

of therapy, NEs hold merits especially for their rapid inflammation chemotaxis and deep tumor penetration and thus have been focused on as the drug carrier to improve drug accumulation in solid tumor tissues. However, their distinct tumoricidal ability and immunoregulatory potency has been ignored in cell therapy owing to their easy-to-change phenotypes in tumor environment.[6,42] Herein, we propose a non-genetic engineering approach to reprogram the NEs from a naive or pro-tumor phenotype to an anti-tumor phenotype via IFNγ training and to further strengthen their anti-tumor efficacy via Abraxane-arming for the treatment of lung metastatic TNBC.

In this study, we have addressed the cell source issue for the future application of NE-based therapy. As well known, fresh NEs from healthy donors lack satisfactory effectiveness in treatment of advanced solid tumors,[12] while those from "super donors" possess improved potency but are limited by the insufficient amount of "super donors." Worse, the isolated NEs from cancerous patients exert a pro-tumor response due to the plasticity of NEs in tumor environment.[31–33] To avoid these disadvantages, we fabricated ultra-NEs by IFNγ-induced non-genetic reprogramming, which converted NEs from both autologous and allogeneic NEs toward an anti-tumor phenotype and potentiated their tumoricidal ability and immunoregulatory potency. Notably, this reprogramming approach endowed the infused ultra-NEs maintaining tumoricidal and immunoregulatory potency in lung metastases, circumventing the liable phenotype conversion even in tumor environment. Further, the prolonged survival of ultra-NEs favors the accomplishment of non-clinic and clinic quality control of NE-based therapy. Leveraging these merits, ultra-NEs deserve as the potential cell source for future application.

To further potentiate the therapeutic effect in hard-to-cure advanced solid tumors with metastases, such as TNBC, ultra-NEs were weaponed with Abraxane based on our previous methods[43–45] to gain meta-NEs, which combined the merits of ultra-NEs and superior cytotoxicity of Abraxane. Moreover, we had demonstrated that NEs weaponed with Abraxane possessed superior tumor infiltration and robust anti-tumor effect.[44,45] Although several innovative treating regimens have been exploited in TNBC therapy such as immune checkpoint,[46] the low response rate implies that only a few patients suffering TNBC lung metastases could benefit from this therapy.[47] Cytotoxic chemotherapy remains the mainstream treatment in advanced TNBC but is also restricted by the unsatisfactory drug content in primary and metastatic tumors, especially the small metastatic foci.[48,49] Herein, leveraging the chemotaxis and tumor-penetrating abilities of ultra-NEs, meta-NEs achieved the highly increased accumulation of PTX in primary tumors and lung metastatic foci. We demonstrated that large amounts of the reinfused meta-NEs were found in the primary tumor and metastatic foci, even in the depth, verifying the high targeting efficiency of meta-NEs *in vivo*, which benefits the subsequent tumor killing and immunoregulation.

The efficacy of meta-NEs in three mice models with advanced metastatic TNBC indicated the enhanced potency on the suppression of tumor growth and metastases, especially the inhibition rate of lung metastases up to 97%, and an obviously prolonged survival. The probable reasons include the efficient TNBC chemotaxis of meta-NEs leading to the high accumulation of PTX, the strengthened tumoricidal ability of ultra-NEs themselves, and the improved immunoregulatory potency on tumor-infiltrated $CD8^+$T cells and NK cells. Promisingly, meta-NEs, which synergize the cytotoxicity of PTX and potency of NEs, could circumvent the heterogeneity bottleneck of solid tumors faced by the single CAR-engineered immune cells.[50] The reinfusion of amounts of meta-NEs had no toxicity on normal tissues and no severe immune response, probably owing to the shorter lifespan and weaker immune responses compared with other immune cells such as T cells, thus implying the non-genetic reprogrammed meta-NEs with broad potential prospects because of their safety. Notably, meta-NEs are fabricated by the treatment of NEs with IFNγ and Abraxane, of which NEs, IFNγ, or Abraxane have been separately applied in clinic, suggesting the clinical potential of meta-NEs. However, the meta-NEs still need great efforts to be applied in future, including the establishment of quality control criteria and integrated and automated instruments. More importantly, meta-NEs present different pharmacokinetic profile and action mechanism from traditional medicines due to the "alive" characteristics, which also requires additional research to make them qualified "living drugs."

In conclusion, we designed a non-genetic engineering strategy for anti-tumor NE production and maintenance and further potentiated their cytotoxicity by arming anti-tumor NEs with cytotoxic drug PTX-albumin-bound nanoparticles. The obtained meta-NEs demonstrated effective chemo-immunotherapy against lung metastatic TNBC through the combination effect of strengthened tumoricidal and immunoregulatory potency of NEs and accumulated cytotoxicity of PTX, which provided a neutrophil-based therapy for advanced TNBC. Notably, the expanded cell source of NEs with demonstrated safety and effectiveness would favor the development of clinical NE therapy. Further, the non-genetic reprograming strategy could be utilized in other immune cells, such as macrophages, NK cells, and so on, to improve their therapeutic effect in tumor therapy, which requires further exploration.

Limitations of the study

This study has several limitations. First, the anti-tumor potential of meta-NEs was validated using blood samples from a limited number of clinical patients and healthy individuals. Future studies should include larger patient cohorts covering a broader range of breast cancer subtypes for further validation. Second, our conclusion regarding the immunomodulatory potential of meta-NEs are primarily based on observational experimental findings and lack systematic mechanistic analysis. Future investigations should employ diverse molecular biology approaches to comprehensively elucidate the immunomodulatory mechanisms of meta-NEs.

Furthermore, the chemo-immunotherapy potential of meta-NEs has only been demonstrated in mouse models. However, even humanized mouse models cannot fully recapitulate the heterogeneity and immune microenvironment of human tumors. Therefore, future clinical trials are warranted to systematically validate the efficacy of this strategy. The current study has only performed preliminary safety assessments of meta-NEs; subsequent research should systematically evaluate the long-term toxicity of this therapeutic strategy to fully establish its safety

profile. Additionally, this strategy could be extended to tumor types beyond TNBC, thereby broadening the therapeutic applicability of this non-genetic reprograming strategy.

RESOURCE AVAILABILITY

Lead contact

Further information and requests for resources and reagents should be directed to and will be fulfilled by the lead contact, Can Zhang (zhangcan@cpu.edu.cn).

Materials availability

This study did not generate new unique reagents.

Data and code availability

- All data reported in this paper will be shared by the lead contact upon request.
- This study does not report original code. Transcriptome data have been deposited at NCBI Sequence Read Archive (SRA) and are publicly available as of the date of publication. Accession numbers are listed in the key resources table.
- Any additional information required to reanalyze the data reported in this work paper is available from the lead contact upon request.

ACKNOWLEDGMENTS

We thank the Public Platform of the State Key Laboratory of Natural Medicines and Jiangsu Key Laboratory of Drug Design and Optimization for assistance with the pathological-section imaging and flow cytometry and Nanjing Jingdu Hospital for assistance with the recruitment of volunteers and peripheral blood collection. We are grateful to Yumeng Shen and Ping Zhou for their help in performing fluorescence-activated cell sorting experiments. This work was supported by the National Natural Science Foundation of China (82130102 to C.Z., 82373823 to M.H., 92159304 to C.Z., and 81930099 to C.Z.), the Natural Science Foundation of Jiangsu Province (BK20212011 to C.Z. and BK20230104 to M.H.), the "Open Competition to Select the Best Candidates" Key Technology Program for Nucleic Acid Drugs of NCTIB (NCTIB2022HS01014 to C.Z.), and the National Major Scientific and Technological Special Project for "Significant New Drugs Development" (2019ZX09301163 to C.Z.).

AUTHOR CONTRIBUTIONS

M.H., Q.H., and X.L. contributed equally to this work. M.H. performed the animal experiments and analyzed the data and subsequently addressed the reviewers' comments by performing additional experiments and supervised these experiments. She also wrote the original draft of the manuscript and contributed to its review and editing. Q.H. designed and conducted most of the experiments in original draft and analyzed the data. X.L. assisted in the experiments of human NEs and subsequently addressed the reviewers' comments by performing additional experiments. Y.C. assisted with the RNA-seq analysis and cell killing experiments. S.H. provided assistance on experimental design and tissue clearing. C.H assisted in the experiments and subsequently addressed the reviewers' comments by performing additional experiments. Sijia Chen, Y.L., and K.L. assisted in *in vivo* pharmacodynamic experiments. L.X. assisted in data analysis. L.Z and Shanshan Chen assisted in breeding of mice. C.J. wrote the original manuscript and supervised most of the experiments. C.Z. conceived the project and supervised all experiments.

DECLARATION OF INTERESTS

The authors declare no competing interests.

STAR★METHODS

Detailed methods are provided in the online version of this paper and include the following:

- KEY RESOURCES TABLE
- EXPERIMENTAL MODEL AND STUDY PARTICIPANT DETAILS
  - Cell lines
  - Primary cells
  - Mice
  - The 4T1 lung metastasis model
  - The spontaneous 4T1 lung metastasis model
  - The humanized triple-negative breast cancer mouse model
- METHOD DETAILS
  - Preparation of ultra-NEs
  - Characterization of NEs and ultra-NEs
  - Preparation and characterization of *meta*-NEs
  - Chemotaxis of *meta*-NEs *in vitro*
  - Chemotaxis of *meta*-NEs *in vivo*
  - *In vivo* biodistribution of *meta*-NEs
  - Anti-tumor efficacy and safety of *meta*-NEs
  - Evaluation the tumoricidal and immunoregulatory potency of *meta*-NEs *in vitro*
  - Evaluation the tumoricidal and immunoregulatory potency of *meta*-NEs *in vivo*
- QUANTIFICATION AND STATISTICAL ANALYSIS

SUPPLEMENTAL INFORMATION

Supplemental information can be found online at https://doi.org/10.1016/j.xcrm.2026.102868.

Received: September 16, 2025
Revised: February 27, 2026
Accepted: May 18, 2026
Published: June 12, 2026

REFERENCES

1. June, C.H., and Sadelain, M. (2018). Chimeric Antigen Receptor Therapy. N. Engl. J. Med. *379*, 64–73. https://doi.org/10.1056/NEJMra1706169.
2. Diorio, C., Teachey, D.T., and Grupp, S.A. (2025). Allogeneic chimeric antigen receptor cell therapies for cancer: progress made and remaining roadblocks. Nat. Rev. Clin. Oncol. *22*, 10–27. https://doi.org/10.1038/s41571-024-00959-y.
3. Du, B., Qin, J., Lin, B., Zhang, J., Li, D., and Liu, M. (2025). CAR-T therapy in solid tumors. Cancer Cell *43*, 665–679. https://doi.org/10.1016/j.ccell.2025.03.019.
4. Reglero-Real, N., Pérez-Gutiérrez, L., Yoshimura, A., Rolas, L., Garrido-Mesa, J., Barkaway, A., Pickworth, C., Saleeb, R.S., Gonzalez-Nuñez, M., Austin-Williams, S.N., et al. (2021). Autophagy modulates endothelial junctions to restrain neutrophil diapedesis during inflammation. Immunity *54*, 1989–2004.e9. https://doi.org/10.1016/j.immuni.2021.07.012.
5. Ballesteros, I., Rubio-Ponce, A., Genua, M., Lusito, E., Kwok, I., Fernández-Calvo, G., Khoyratty, T.E., van Grinsven, E., González-Hernández, S., Nicolás-Ávila, J.Á., et al. (2020). Co-option of Neutrophil Fates by Tissue Environments. Cell *183*, 1282–1297.e18. https://doi.org/10.1016/j.cell.2020.10.003.
6. Wu, H., Li, W., Hao, M., Wang, Y., Xue, L., Ju, C., and Zhang, C. (2022). An EPR-Independent extravasation Strategy: Deformable leukocytes as vehicles for improved solid tumor therapy. Adv. Drug Deliv. Rev. *187*, 114380. https://doi.org/10.1016/j.addr.2022.114380.
7. Cui, C., Chakraborty, K., Tang, X.A., Zhou, G., Schoenfelt, K.Q., Becker, K.M., Hoffman, A., Chang, Y.F., Blank, A., Reardon, C.A., et al. (2021). Neutrophil elastase selectively kills cancer cells and attenuates

tumorigenesis. Cell *184*, 3163–3177.e21. https://doi.org/10.1016/j.cell.2021.04.016.

8. Jaillon, S., Ponzetta, A., Di Mitri, D., Santoni, A., Bonecchi, R., and Mantovani, A. (2020). Neutrophil diversity and plasticity in tumour progression and therapy. Nat. Rev. Cancer *20*, 485–503. https://doi.org/10.1038/s41568-020-0281-y.
9. Mantovani, A., Cassatella, M.A., Costantini, C., and Jaillon, S. (2011). Neutrophils in the activation and regulation of innate and adaptive immunity. Nat. Rev. Immunol. *11*, 519–531. https://doi.org/10.1038/nri3024.
10. Chen, Z., Zhang, G., Ren, X., Yao, Z., Zhou, Q., Ren, X., Chen, S., Xu, L., Sun, K., Zeng, Q., et al. (2023). Cross-talk between Myeloid and B Cells Shapes the Distinct Microenvironments of Primary and Secondary Liver Cancer. Cancer Res. *83*, 3544–3561. https://doi.org/10.1158/0008-5472.Can-23-0193.
11. Keller, M., Spanou, Z., Schaerli, P., Britschgi, M., Yawalkar, N., Seitz, M., Villiger, P.M., and Pichler, W.J. (2005). T cell-regulated neutrophilic inflammation in autoinflammatory diseases. J. Immunol. *175*, 7678–7686. https://doi.org/10.4049/jimmunol.175.11.7678.
12. Maharaj, D., Vianna, P.G., Ward, W., Messina, A.J., Rayborn, T., Gouvea, J.V., Hammer, R.D., and Cui, Z. (2017). Young donor white blood cell immunotherapy induces extensive tumor necrosis in advanced-stage solid tumors. Heliyon *3*, e00438. https://doi.org/10.1016/j.heliyon.2017.e00438.
13. Cai, W., Fan, T., Xiao, C., Deng, Z., Liu, Y., Li, C., and He, J. (2025). Neutrophils in cancer: At the crucial crossroads of anti-tumor and pro-tumor. Cancer Commun. *45*, 888–913. https://doi.org/10.1002/cac2.70027.
14. Wu, Y., Ma, J., Yang, X., Nan, F., Zhang, T., Ji, S., Rao, D., Feng, H., Gao, K., Gu, X., et al. (2024). Neutrophil profiling illuminates anti-tumor antigen-presenting potency. Cell *187*, 1422–1439.e24. https://doi.org/10.1016/j.cell.2024.02.005.
15. Evrard, M., Kwok, I.W.H., Chong, S.Z., Teng, K.W.W., Becht, E., Chen, J., Sieow, J.L., Penny, H.L., Ching, G.C., Devi, S., et al. (2018). Developmental Analysis of Bone Marrow Neutrophils Reveals Populations Specialized in Expansion, Trafficking, and Effector Functions. Immunity *48*, 364–379.e8. https://doi.org/10.1016/j.immuni.2018.02.002.
16. Fridlender, Z.G., Sun, J., Kim, S., Kapoor, V., Cheng, G., Ling, L., Worthen, G.S., and Albelda, S.M. (2009). Polarization of Tumor-Associated Neutrophil Phenotype by TGF-β: “N1” versus “N2” TAN. Cancer Cell *16*, 183–194. https://doi.org/10.1016/j.ccr.2009.06.017.
17. Flavell, R.A., Sanjabi, S., Wrzesinski, S.H., and Licona-Limón, P. (2010). The polarization of immune cells in the tumour environment by TGFβ. Nat. Rev. Immunol. *10*, 554–567. https://doi.org/10.1038/nri2808.
18. Li, Y., Hu, Q., Li, W., Liu, S., Li, K., Li, X., Du, J., Yu, Z., Wang, C., and Zhang, C. (2021). Simultaneous blockage of contextual TGF-β by cyto-pharmaceuticals to suppress breast cancer metastasis. J. Control. Release *336*, 40–53. https://doi.org/10.1016/j.jconrel.2021.06.012.
19. Ellis, T.N., and Beaman, B.L. (2004). Interferon-γ activation of polymorphonuclear neutrophil function. Immunology *112*, 2–12. https://doi.org/10.1111/j.1365-2567.2004.01849.x.
20. Gocher, A.M., Workman, C.J., and Vignali, D.A.A. (2022). Interferon-γ: teammate or opponent in the tumour microenvironment? Nat. Rev. Immunol. *22*, 158–172. https://doi.org/10.1038/s41577-021-00566-3.
21. Creusat, F., Jouan, Y., Gonzalez, L., Barsac, E., Ilango, G., Lemoine, R., Soulard, D., Hankard, A., Boisseau, C., Guillon, A., et al. (2024). IFN-γ primes bone marrow neutrophils to acquire regulatory functions in severe viral respiratory infections. Sci. Adv. *10*, eadn3257. https://doi.org/10.1126/sciadv.adn3257.
22. Gungabeesoon, J., Gort-Freitas, N.A., Kiss, M., Bolli, E., Messemaker, M., Siwicki, M., Hicham, M., Bill, R., Koch, P., Cianciaruso, C., et al. (2023). A neutrophil response linked to tumor control in immunotherapy. Cell *186*, 1448–1464.e20. https://doi.org/10.1016/j.cell.2023.02.032.
23. Polak, D., and Bohle, B. (2022). Neutrophils-typical atypical antigen presenting cells? Immunol. Lett. *247*, 52–58. https://doi.org/10.1016/j.imlet.2022.04.007.
24. Remsik, J., Tong, X., Kunes, R.Z., Li, M.J., Estrera, R., Snyder, J., Thomson, C., Osman, A.M., Chabot, K., Sener, U.T., et al. (2025). Interferon-γ orchestrates leptomeningeal anti-tumour response. Nature *643*, 1087–1096. https://doi.org/10.1038/s41586-025-09012-z.
25. Castro, F., Cardoso, A.P., Gonçalves, R.M., Serre, K., and Oliveira, M.J. (2018). Interferon-Gamma at the Crossroads of Tumor Immune Surveillance or Evasion. Front. Immunol. *9*, 847. https://doi.org/10.3389/fimmu.2018.00847.
26. Schmid, P., Rugo, H.S., Adams, S., Schneeweiss, A., Barrios, C.H., Iwata, H., Diéras, V., Henschel, V., Molinero, L., Chui, S.Y., et al. (2020). Atezolizumab plus nab-paclitaxel as first-line treatment for unresectable, locally advanced or metastatic triple-negative breast cancer (IMpassion130): updated efficacy results from a randomised, double-blind, placebo-controlled, phase 3 trial. Lancet Oncol. *21*, 44–59. https://doi.org/10.1016/S1470-2045(19)30689-8.
27. van Grinsven, E., Mukherjee, A.K., and Udalova, I.A. (2025). Shaping neutrophil morphology and function: the importance of a segmented nucleus. Nat. Rev. Immunol. *26*, 269–283. https://doi.org/10.1038/s41577-025-01237-3.
28. Liu, J., Li, Z., Li, M., Du, W., Baumeister, W., Yang, J., and Guo, Q. (2023). Vimentin regulates nuclear segmentation in neutrophils. Proc. Natl. Acad. Sci. USA *120*, e2307389120. https://doi.org/10.1073/pnas.
29. Harlin, H., Meng, Y., Peterson, A.C., Zha, Y., Tretiakova, M., Slingluff, C., McKee, M., and Gajewski, T.F. (2009). Chemokine Expression in Melanoma Metastases Associated with CD8(+) T-Cell Recruitment. Cancer Res. *69*, 3077–3085. https://doi.org/10.1158/0008-5472.Can-08-2281.
30. Singhal, S., Bhojnagarwala, P.S., O’Brien, S., Moon, E.K., Garfall, A.L., Rao, A.S., Quatromoni, J.G., Stephen, T.L., Litzky, L., Deshpande, C., et al. (2016). Origin and Role of a Subset of Tumor-Associated Neutrophils with Antigen-Presenting Cell Features in Early-Stage Human Lung Cancer. Cancer Cell *30*, 120–135. https://doi.org/10.1016/j.ccell.2016.06.001.
31. Patel, S., Fu, S., Mastio, J., Dominguez, G.A., Purohit, A., Kossenkov, A., Lin, C., Alicea-Torres, K., Sehgal, M., Nefedova, Y., et al. (2018). Unique pattern of neutrophil migration and function during tumor progression. Nat. Immunol. *19*, 1236–1247. https://doi.org/10.1038/s41590-018-0229-5.
32. Sagiv, J.Y., Michaeli, J., Assi, S., Mishalian, I., Kisos, H., Levy, L., Damti, P., Lumbroso, D., Polyansky, L., Sionov, R.V., et al. (2015). Phenotypic Diversity and Plasticity in Circulating Neutrophil Subpopulations in Cancer. Cell Rep. *10*, 562–573. https://doi.org/10.1016/j.celrep.2014.12.039.
33. Geh, D., Leslie, J., Rumney, R., Reeves, H.L., Bird, T.G., and Mann, D.A. (2022). Neutrophils as potential therapeutic targets in hepatocellular carcinoma. Nat. Rev. Gastro. Hepat. *19*, 257–273. https://doi.org/10.1038/s41575-021-00568-5.
34. Wculek, S.K., and Malanchi, I. (2015). Neutrophils support lung colonization of metastasis-initiating breast cancer cells. Nature *528*, 413–417. https://doi.org/10.1038/nature16140.
35. Coffelt, S.B., Kersten, K., Doornebal, C.W., Weiden, J., Vrijland, K., Hau, C.S., Verstegen, N.J.M., Ciampricotti, M., Hawinkels, L.J.A.C., Jonkers, J., and de Visser, K.E. (2015). IL-17-producing gammadelta T cells and neutrophils conspire to promote breast cancer metastasis. Nature *522*, 345–348. https://doi.org/10.1038/nature14282.
36. Smid, M., Wang, Y., Zhang, Y., Sieuwerts, A.M., Yu, J., Klijn, J.G.M., Foekens, J.A., and Martens, J.W.M. (2008). Subtypes of breast cancer show preferential site of relapse. Cancer Res. *68*, 3108–3114. https://doi.org/10.1158/0008-5472.Can-07-5644.
37. Spörri, R., Joller, N., Hilbi, H., and Oxenius, A. (2008). A novel role for neutrophils as critical activators of NK cells. J. Immunol. *181*, 7121–7130. https://doi.org/10.4049/jimmunol.181.10.7121.

CellPress
OPEN ACCESS

38. Sun, R., Luo, J., Li, D., Shu, Y., Luo, C., Wang, S.S., Qin, J., Zhang, G.M., and Feng, Z.H. (2014). Neutrophils with protumor potential could efficiently suppress tumor growth after cytokine priming and in presence of normal NK cells. Oncotarget *5*, 12621–12634. https://doi.org/10.18632/oncotarget.2181.

39. Kwak, J.W., and Houghton, A.M. (2025). Targeting neutrophils for cancer therapy. Nat. Rev. Drug Discov. *24*, 666–684. https://doi.org/10.1038/s41573-025-01210-8.

40. Cubillos-Ruiz, A., Guo, T., Sokolovska, A., Miller, P.F., Collins, J.J., Lu, T.K., and Lora, J.M. (2021). Engineering living therapeutics with synthetic biology. Nat. Rev. Drug Discov. *20*, 941–960. https://doi.org/10.1038/s41573-021-00285-3.

41. Feigal, E.G., DeWitt, N.D., Cantilena, C., Peck, C., and Stroncek, D. (2019). At the end of the beginning: immunotherapies as living drugs (vol 20, pg 955, 2019). Nat. Immunol. *20*, 1405. https://doi.org/10.1038/s41590-019-0476-0.

42. Li, W., Su, Z., Hao, M., Ju, C., and Zhang, C. (2020). Cytopharmaceuticals: An emerging paradigm for drug delivery. J. Control. Release *328*, 313–324. https://doi.org/10.1016/j.jconrel.2020.08.063.

43. Xue, J., Zhao, Z., Zhang, L., Xue, L., Shen, S., Wen, Y., Wei, Z., Wang, L., Kong, L., Sun, H., et al. (2017). Neutrophil-mediated anticancer drug delivery for suppression of postoperative malignant glioma recurrence. Nat. Nanotechnol. *12*, 692–700. https://doi.org/10.1038/Nnano.2017.54.

44. Ju, C., Wen, Y., Zhang, L., Wang, Q., Xue, L., Shen, J., and Zhang, C. (2019). Neoadjuvant Chemotherapy Based on Abraxane/Human Neutrophils Cytopharmaceuticals with Radiotherapy for Gastric Cancer. Small *15*, e1804191. https://doi.org/10.1002/smll.201804191.

45. Zhang, L., Wang, Q., Dai, Y., Chen, J., Wu, T., Ju, C., Xue, L., and Zhang, C. (2023). New-generation cytopharmaceuticals with powerfully boosted extravasation for enhanced cancer therapy. J. Control. Release *359*, 116–131. https://doi.org/10.1016/j.jconrel.2023.05.037.

46. Nanda, R., Chow, L.Q.M., Dees, E.C., Berger, R., Gupta, S., Geva, R., Pusztai, L., Pathiraja, K., Aktan, G., Cheng, J.D., et al. (2016). Pembrolizumab in Patients With Advanced Triple-Negative Breast Cancer: Phase Ib KEYNOTE-012 Study. J. Clin. Oncol. *34*, 2460–2467. https://doi.org/10.1200/Jco.2015.64.8931.

47. Voorwerk, L., Slagter, M., Horlings, H.M., Sikorska, K., van de Vijver, K.K., de Maaker, M., Nederlof, I., Kluin, R.J.C., Warren, S., Ong, S., et al. (2019). Immune induction strategies in metastatic triple-negative breast cancer to enhance the sensitivity to PD-1 blockade: the TONIC trial. Nat. Med. *25*, 920–928. https://doi.org/10.1038/s41591-019-0432-4.

48. Wan, L., Pantel, K., and Kang, Y. (2013). Tumor metastasis: moving new biological insights into the clinic. Nat. Med. *19*, 1450–1464. https://doi.org/10.1038/nm.3391.

49. Schroeder, A., Heller, D.A., Winslow, M.M., Dahlman, J.E., Pratt, G.W., Langer, R., Jacks, T., and Anderson, D.G. (2012). Treating metastatic cancer with nanotechnology. Nat. Rev. Cancer *12*, 39–50. https://doi.org/10.1038/nrc3180.

50. Baker, D.J., Arany, Z., Baur, J.A., Epstein, J.A., and June, C.H. (2023). CAR T therapy beyond cancer: the evolution of a living drug. Nature *619*, 707–715. https://doi.org/10.1038/s41586-023-06243-w.

51. Hao, M., Hou, S., Li, W., Li, K., Xue, L., Hu, Q., Zhu, L., Chen, Y., Sun, H., Ju, C., and Zhang, C. (2020). Combination of metabolic intervention and T cell therapy enhances solid tumor immunotherapy. Sci. Transl. Med. *12*, eaaz6667. https://doi.org/10.1126/scitranslmed.aaz6667.

STAR★METHODS

KEY RESOURCES TABLE

REAGENT or RESOURCE	SOURCE	IDENTIFIER
Antibodies		
Anti-mouse Ly-6G Antibody(Alexa Fluro 488)	BioLegend (USA)	Cat# 127626; RRID: AB_2561340
Anti-mouse/human CD11b Antibody (APC)	BioLegend (USA)	Cat# 101211; RRID: AB_312794
Anti-mouseTNF-α Antibody (PE)	BioLegend (USA)	Cat# 506306; RRID: AB_315427
Anti-mouse CD8a Antibody (APC/ Cyanine7)	BioLegend (USA)	Cat# 100714; RRID: AB_312753
Anti-mouseCD49b Antibody (Pacific Blue™)	BioLegend (USA)	Cat# 108917; RRID: AB_2249376
Anti-mouse IFN-γ Antibody Antibody	BioLegend (USA)	Cat# 505810; RRID: AB_315404
Anti-human/mouse Granzyme B Antibody (FITC)	BioLegend (USA)	Cat# 515403; RRID: AB_2114575
Anti-mouse CD80 Antibody (FITC)	BioLegend (USA)	Cat# 104706; RRID: AB_313127
Anti-mouse CD86 Antibody ((Alexa Fluor 647)	BioLegend (USA)	Cat# 105019; RRID: AB_493465
Anti-mouse ICAM-1 Antibody (PE)	Invitrogen (USA)	Cat# 12-0541-81; RRID: AB_465706
Anti-mouse MHCII Antibody (PE)	BioLegend (USA)	Cat# 116407; RRID: AB_313726
Anti-mouse CD45 Antibody (PE)	BioLegend (USA)	Cat# 147711; RRID: AB_2563597
Anti-mouse F4/80 Antibody (APC)	BioLegend (USA)	Cat# 123115; RRID: AB_893493
Anti-mouse CD11c Antibody (FITC)	BioLegend (USA)	Cat# 117305; RRID: AB_313774
Anti-Mouse CD3 SAFIRE Purified	BioGem (USA)	Cat# 05112-25; RRID: AB_3099697
Anti-Mouse CD28 SAFIRE Purified	BioGem (USA)	Cat# 10312-25; RRID: AB_3099698
Bacterial and virus strains		
Firefly Luciferase-mCherry Lentivirus	GeneChem (China)	N/A
mCherry expressing lentivirus	GeneChem (China)	N/A
Cell lines		
4T1	ATCC	N/A
EMT6	ATCC	N/A
HUVEC	ATCC	N/A
MDA-MB-231	ATCC	N/A
Chemicals, peptides, and recombinant proteins		
RPMI1640	HyClone (USA)	N/A
DMEM	HyClone (USA)	N/A
Fetal bovine serum	HyClone (USA)	N/A
ACK lysis buffer	Haoyang (China)	N/A
PBS	HyClone (USA)	N/A
10xPBS	Solarbio (China)	Cat# P1022
Percoll	HyClone (USA)	Cat# 17-0819-09
Recombinant mouse IFNγ	Prospec-Tany	Cat# cyt-358
Recombinant human IFNγ	Thermo (USA)	Cat# 300-02-100UG
RNA isolater Total RNA Extraction Reagent	Vazyme (China)	Cat# R401-01
HiScript III RT SuperMix for qPCR	Vazyme (China)	Cat# R323-01
Hieff qPCR SYBR Green Master Mix	YEASEN (China)	Cat# 11201ES03
Ionomycin	YEASEN (China)	Cat# 50402ES03
PMA	Sigma-Aldrich	Cat# P1585
Brefeldin A	Beyotime (China)	Cat# S1536

(*Continued on next page*)

CellPress
OPEN ACCESS

Continued

REAGENT or RESOURCE	SOURCE	IDENTIFIER
DAF-FM DA	Beyotime (China)	Cat# S0019M
Abraxane	BMS (USA)	N/A
Formyl-Met-Leu-Phe	Sigma-Aldrich	Cat# 59880-97-6
4% PFA	Lingfeng (China)	Cat# 30525-89-4
DiD	YEASEN (China)	Cat# 40758ES25
DiR	YEASEN (China)	Cat# 40757ES25
D-Luciferin Potassium Salt	YEASEN (China)	Cat# 40902ES02
FITC	Sigma (USA)	Cat# 46950-50MG-F
LysoTracker red DND-99	YEASEN (China)	Cat# 40739ES50
CellTrace™	Thermo (USA)	Cat# C34554
DNAase	Sigma (USA)	Cat# 11284932001
Hank's Balanced Salt Solution	Solarbio (China)	N/A
DAPI	Sigma (USA)	Cat# D9542
Tissue clearing reagent	Abcam (UK)	Cat# ab243298
Critical commercial assays		
Apoptosis Detection Kit	Vazyme (China)	Cat# A211-02
Nitric oxide detection kit	Beyotime (China)	Cat# S0021S
Oxidative stress detection kit	Enzo Life Sciences (USA)	Cat# ENZ-51042-K500
Hydrogen peroxide assay kit	Beyotime (China)	Cat# S0038
Human neutrophil isolation solution®	Haoyang (China)	Cat# LZS11131
EasySep Mouse CD8$^+$T cell Isolation Kit	StemCell (Canada)	Cat# 19853
Deposited data		
RNA-seq data	This paper	NCBI BioProject: PRJNA1464142
Experimental models		
BALB/c mice	GemPharmatech	N/A
NSFG mice	Qinglongshan Biotechnology	N/A
Oligonucleotides		
Primers for *Actb/Mmp9/Arg1/Prok2/Ccl2/Icam1/Tnf/Trail/Ifna/Ifnb1/Cxcl9/Cxcl10*, see Table S2	Genscript (China)	N/A
Software and algorithms		
Graphpad Prism 10.1.2	Graphpad software	https://www.graphpad.com
ImageJ	N/A	https://imagej.net/ij/
Living Image® Software	PerkinElmer	https://www.perkinelmer.com

EXPERIMENTAL MODEL AND STUDY PARTICIPANT DETAILS

Cell lines

The murine triple-negative breast cancer cells (4T1 and EMT6), human umbilical vein endothelial cells (HUVEC), and human triple-negative breast cancer cells (MDA-MB-231) were purchased from the American Type Culture Collection (ATCC). For *in vivo* imaging, 4T1-Luci and mCherry-4T1 cell lines were generated by stable transfection of parental 4T1 cells with firefly luciferase- or mCherry-expressing lentivirus (GeneChem) according to the manufacturer's instructions. Cells were cultured in DMEM high-glucose medium (EMT6, MDA-MB-231) or RPMI-1640 (4T1, HUVEC), supplemented with 10% fetal bovine serum (FBS), 100 U/mL penicillin, and 100 μg/mL streptomycin at 37°C in a humidified 5% CO_2 atmosphere. All cell lines were authenticated by STR profiling and routinely confirmed mycoplasma-free. Phenotypic authenticity was verified through morphological and growth kinetic analysis.

Primary cells

Peripheral blood mononuclear cells (PBMCs) were isolated from venous blood of healthy donors (n = 3 healthy female donors, aged 20–60 years). Written informed consent was obtained from all donors prior to blood collection, and the study was approved by the Ethics Committee of Nanjing Jingdu Hospital (Approval No. DZQH-KYLLFS-23-29). For isolation, whole blood was mixed 1:1 (*v/v*) with Human PBMC Isolation Solution (Beijing Bestopcell TechnologyCo., LTD; BA3332) in sterile tubes. The mixture was centrifuged

at 700×g for 30 min at 20°C. Following centrifugation, PBMCs were collected from the middle white buffy coat layer (between plasma and isolation solution), transferred to a new tube, and washed twice with ice-cold phosphate-buffered saline (PBS) by centrifugation at 300×g for 5 min per wash. The cell pellet was then resuspended in PBS or the appropriate assay buffer for further use.

Human NEs were isolated from venous blood of healthy donors and triple negative breast cancer patients (n = 9 healthy female donors and n = 9 female triple-negative breast cancer patients, aged 20–60 years) and the study was also approved by the Ethics Committee of Nanjing Jingdu Hospital (Approval No. DZQH-KYLLFS-23-29). Written informed consent was obtained from all donors prior to blood collection, and the study also was approved by the Ethics Committee of Nanjing Jingdu Hospital. For isolation, whole blood was mixed 1:1 (*v/v*) with human neutrophil isolation solution (Tianjin Haoyang Biological Products Technology Co., LTD; LZS11131), followed by centrifugation at 600×g for 30 min at 4°C. NEs in the lower layer were collected and washed three times with ice-cold PBS using centrifugation at 300×g for 5 min per wash. The final NE pellet was resuspended in PBS or the appropriate assay buffer for further use.

For murine NEs, NEs were isolated from blood or bone marrow using continuous Percoll gradient centrifugation method. Briefly, blood and bones from BALB/c mice or tumor-bearing mice were collected. The cells from blood were directly centrifugated at 200 g for 10 min, while that from the bone marrow were firstly flushed from the bone with RPMI 1640 medium and centrifugation at 200*g* for 3 min. Then, the red blood cells were removed by ACK lysis buffer, followed by centrifugation at 200*g* for 3 min. The obtained cells were resuspended in RPMI 1640 medium to gain a unicellular suspension, which was added onto a mixture solution consisting of 55%, 65% and 75% (*v: v*) Percoll in PBS and centrifugated at 1000 g for 30 min. NEs were collected at the interface of the 65% and 75% fractions and washed by ice-cold PBS thrice for *in vitro* and *in vivo* studies. The viability was assayed using the Annexin V-FITC Apoptosis Detection Kit (Vazyme) according to the manufacturer's instructions. The purity was determined using flow cytometry (Attune NxT, ThermoFisher Scientific) through analyzing the positive ratios of anti-mouse Ly6G antibody and anti-mouse CD11b antibody.

Mice

Given that triple negative breast cancer predominantly occurs in females and represents the primary patient population for this disease, female mice were used in this study. BALB/c mice (female, 6–8 weeks, 18–22 g) were provided by the GemPharmatech Co. Ltd. NSFG mice (female, 7 weeks, 17–22 g) were provided by Jiangsu Qinglongshan Biotechnology Co. Ltd. All mice were fed in a pathogen-free environment at 22°C temperature and 50% humidity. All animal care and experiments were performed according to the guidelines approved by the requirements of China Pharmaceutical University Institutional Animal Care and Use Committee (Approval No. YSL-2024-10-016).

The 4T1 lung metastasis model

Mouse 4T1 lung metastasis model was constructed by intravenously injection of 3×10^5 4T1 (Luci-4T1 or mCherry-4T1) cells suspended in saline (100 μL) into BALB/c mice for 10 days.

The spontaneous 4T1 lung metastasis model

The spontaneous 4T1 lung metastasis model was established by injection of 5×10^5 4T1-Luci cells into the right mammary fat pad of female BALB/c mice for 20 days, or the primary tumor was resected at Day 18 to construct the *in situ* resection model with spontaneous 4T1 lung metastasis.

The humanized triple-negative breast cancer mouse model

Female NSFG mice were intravenously injected with 1×10^6 PBMCs per mouse via the tail vein. Subsequently, MDA-MB-231 cells were inoculated into the right mammary fat pad of the mice to establish the humanized triple-negative breast cancer mouse model.

METHOD DETAILS

Preparation of ultra-NEs

NEs were incubated with 100 U/mL of recombinant mouse IFNγ (Prospec-Tany) or human IFNγ (Thermo) for 12 h at 37°C in a humidified environment containing 5% CO_2 to gain ultra-NEs. The viability of ultra-NEs after *in vitro* culture for 12 h were evaluated using the Annexin V-FITC Apoptosis Detection Kit according to the manufacturer's instructions.

Characterization of NEs and ultra-NEs

For determining the effect of IFNγ on the nuclear morphology of NEs, the cells were isolated and adjusted to a density of 1×10^6 cells/mL. One milliliter of the cell suspension was added to a confocal dish and incubated at 37°C in a 5% CO_2 incubator for 30 min to allow cell adherence. The supernatant was discarded, and the dish was air-dried at room temperature. Cells were fixed in 70% ethanol at 4°C, followed by staining with diluted 1×modified Giemsa solution for 15 min. After staining, the cells were rinsed three times with purified water. Following air-drying, cell morphology was observed under an upright microscope (Olympus, BX53) using a 100×oil immersion objective.

CellPress
OPEN ACCESS

For determining the effect of IFNγ on the survival of NEs, NEs were divided into two groups: one group was untreated NEs, and the other group was NEs stimulated with IFNγ (ultra-NEs). Cell viability was detected at 12 h, 24 h and 48 h, respectively.

For gene expression assay, healthy donor derived NEs were treated with or without IFNγ. Total RNA of 1×10^6 NEs or ultra-NEs from different source was isolated by using RNA isolater Total RNA Extraction Reagent (Vazyme Biotech Co., Ltd), which was then reverse-transcribed into cDNA using the HiScript III RT SuperMix for qPCR (Vazyme Biotech Co., Ltd). Converted cDNA (10 ng) was used for each reaction, using Hieff qPCR SYBR Green Master Mix (11201ES03, YEASEN) and 200 nmol/L of forward or reverse primer set. Analyses were done using the StepOnePlus (Applied Biosystems). β-actin (Actb) was used as a reference gene.

For gene enrichment pathway analysis, healthy donor derived NEs were treated with or without IFNγ. RNA-seq analysis involved retaining genes with TPM >1 in ≥2 samples, performing PCA on 1,000 highly variable genes using prcomp (scaled/centered), and conducting Ward's hierarchical clustering (distance: 1 - Pearson r) with row z-score-normalized heatmaps. Differential expression was determined at $|log_2FC| \geq 1$ and padj <0.05, followed by GO enrichment (clusterProfiler; Ensembl IDs, org.Hs.e.g.,.db). Separate analyses were performed for two Gene Ontology branches: molecular function (MF) and biological process (BP). The Benjamini-Hochberg (BH) method was used for *p*-value adjustment. Differential expression was determined at $|log_2FC| \geq 1$ and padj <0.05, followed by KEGG pathway enrichment analysis (clusterProfiler; Entrez ID, org.Hs.e.g.,.db). The species parameter was set to "hsa" (Homo sapiens). Focused analyses were performed on matched entries extracted from all KEGG results, and the categorized dot plots only show pathways with significant ORA results (padj <0.05). All plots were generated in R (ggplot2) and composited in Python (matplotlib/PIL) at 600 DPI using R v4.x and relevant packages.

For determining antigen-presenting ability of ultra-NEs derived from tumor-bearing mice, NEs were isolated from healthy or tumor-bearing mice. Among this, the tumor-bearing mice's NEs were treated with IFNγ (20 ng/mL) for 12 h to obtain the ultra-NEs. Then, the healthy mice's NEs, tumor-bearing mice's NEs and tumor-bearing mice's ultra-NEs were collected and stained with anti-mouse CD80 antibody, anti-mouse CD86 antibody and anti-mouse MHC II antibody, respectively. The fluorescence intensity was determined by using flow cytometry.

For determining T cells activation ability of ultra-NEs derived from tumor-bearing mice, tumor-bearing mice's NEs or ultra-NEs were co-cultured with tumor-infiltrating $CD8^+$T cells at a ratio of 1:2 for 12 h. $CD8^+$T cells were collected and incubated with 1 μM ionomycin and 50 ng/mL PMA in the presence of 5 μg/mL brefeldin A for 4 h. Then the cells were collected, and the expression of IFNγ and TNFα on $CD8^+$T cells was analyzed by flow cytometry.

For measurement of RNS and ROS, 1×10^6 NEs or ultra-NEs from different source were first treated with 100 nM PMA for 2 h. Then, the expression of RNS by NEs or ultra-NEs was detected by flow cytometry with DAF-FM DA (Beyotime) and the secretion of NO was quantified by a nitric oxide detection kit (Beyotime). The expression of ROS by NEs or ultra-NEs was detected by flow cytometry with an Oxidative stress detection kit (Enzo Life Sciences) and the secretion of H_2O_2 was quantified with a hydrogen peroxide assay kit (Beyotime). Meanwhile, the expression of TNFα by NEs or ultra-NEs was analyzed using flow cytometry by staining with anti-mouse TNFα antibody.

For determining the anti-tumor potency, 1×10^4 mCherry-4T1 cells were plated on a chamber slide. After culture for 12 h, 2×10^5 NEs or ultra-NEs were added into the culture. Live imaging of mCherry and bright filed were taken every 10 min using the Cytation 1 (BioTek) cell imaging reader. The xCELLigence station was placed in an incubator at 37°C with 5% CO_2. Then 4T1 tumor cells (7.5×10^3/100 μL) were seeded in each well with 10% FBS RPMI 1640 medium. Once the tumor cells had attached and doubled to 1.5×10^4 tumor cells per well, each plate was added with 3×10^5 NEs or ultra-NEs. The tumor cell index was dynamically recorded as cell proliferation parameter every 5 min until 36 h.

For cytotoxicity analysis of IFNγ, 5×10^3 MDA-MB-231-luci cells were plated on a 96-well plate. 20 ng/mL IFNγ, 2.5×10^4 NEs with or without IFNγ treating were then co-incubated with MDA-MB-231-luci cells for 24 h at 37°C. The luminescence intensity was measured by microplate reader.

Preparation and characterization of *meta*-NEs

For preparation of FITC-Abraxane, FITC (6 mg/mL in DMSO, Sigma) was added into the solution of Abraxane (2 mg/mL PTX, Celgene) under magnetic stirring for 2 h at room temperature, which was then purified using a dialysis bag (MWCO = 10 KD, Solarbio) against PBS at 4°C. The particle size and zeta potential of FITC-Abraxane were measured by a dynamic light scattering (DLS) analyzer (BrookHaven Instrument).

Meta-NEs or FITC labeled *meta*-NEs were obtained by incubating ultra-NEs with Abraxane or FITC-Abraxane (1 mg/mL PTX) for 30 min at 37°C. For confocal imaging, FITC labeled *meta*-NEs were stained by Hoechst 33342 and observed by CLSM (ZEISS, LSM 880 with Airyscan). For confocal microscopy imaging of the uptake pathway of *meta*-NEs, Abraxane were labeled with FITC (Green), lysosomes were labeled with LysoTracker red DND-99 (Red), cell nuclei were labeled with Hoechst 33342 (Blue) and observed by CLSM (ZEISS, LSM 880 with Airyscan). The amount of PTX in *meta*-NEs was determined by using high-performance liquid chromatography (HPLC-LC-2010A HT, SHIMADZU).

In vitro stability of *meta*-NEs was evaluated under different conditions. Briefly, *meta*-NEs (1×10^6 cells per well) were seeded in 24-well plate and incubated with PBS, 50% serum RPMI 1640 medium, or 4T1 TCM. At different periods (0, 2, 4, 6, and 8 h), the amounts of PTX in the NEs and released in the supernatant medium were determined by using HPLC.

The NETs release was observed by incubating *meta*-NEs (1×10^6 cells per well) with 100 nM PMA for 4 h, followed by fixing with 4% paraformaldehyde (PFA) for 15 min at 4°C. Then, the cells were stained by Hoechst 33324, and observed by using CLSM (ZEISS, LSM 880 with Airyscan).

To evaluate the *in vitro* cytotoxicity, NEs, ultra-NEs or *meta*-NEs (2×10^5 cells/well) were added to each well with mCherry-4T1 cells (1×10^4 cells/well). After 12 h, each well was photographed randomly by an inverted fluorescence microscope (Olympus). The relative area of mCherry signal was investigated by Multi-functional Microplate Reader (BioTek, Cytation 5 imaging reader).

Chemotaxis of *meta*-NEs *in vitro*

The chemotactic function was investigated by a transwell migration assay. NEs, ultra-NEs, NEs/Abraxane or *meta*-NEs (1×10^6 cells/mL) were added into the upper chamber of the transwell (3 μm, 24 mm, Corning), while the lower chamber contained different concentration (1, 10, 100 nM) of fMLP (Sigma). Meanwhile, fMLP-free medium in the lower chamber was set as controls. After incubation for 8 h, the number of NEs in the lower chamber was counted using the automatic cell counter (Countstar BioMed). The chemotaxis index [$(N_{fMLP}-N_{blank})/N_{blank}$] was calculated, where N_{fMLP} and N_{blank} are the numbers of NEs in the lower chamber with or without fMLP, respectively.

To evaluate the capability of chemotactic migration across the blood vessel, we first established a HUVEC monolayer model on the membrane of transwell (3 μm, 24 mm, Corning), which showed a TEER over 300 Ω cm^2 by using a Millicell-ERS voltohmmeter (Millipore). Then, Abraxane (4.5 μg PTX), NEs/Abraxane (1×10^6 cells, 4.5 μg PTX) or *meta*-NEs (1×10^6 cells, 4.5 μg PTX) were added into separate upper chamber, while 10 nM fMLP was added into the lower chamber. Meanwhile, fMLP-free medium in the lower chamber was set as controls. After incubation for 8 h, the amount of PTX in the upper chamber, lower chamber, and in the middle HUVEC cells was quantified by using HPLC.

Chemotaxis of *meta*-NEs *in vivo*

To evaluate the *in vivo* chemotaxis of *meta*-NEs toward tumors, *meta*-NEs were labeled with DiD cell staining solution. After injection of 200 μL of 4T1 TCM subcutaneously into the abdomen of healthy female BALB/c mice which simulates the local tumor inflammation microenvironment for 4 h, 1.1×10^7 DiD-labeled ultra-NEs or DiD and FITC labeled *meta*-NEs were injected intravenously into the model mice. After 2 h, the mice were anesthetized and the monolayer of the subcutaneous venous plexus at the abdominal injection site was separated. The intravascular fluorescence signals of DiD and FITC were observed using CLSM. The velocity of cells was calculated with Imaris.

For further analysis of *in vivo* chemotaxis, the proportion of exogenous NEs in the lungs were determined in mouse 4T1 lung metastasis model. The tumor-bearing mice were intravenously injected with CellTrace Far Red (Life Technologies)-labeled NEs formulations (1.1×10^7 cells/mice). The lungs were harvested at different time (4, 12, 24, 48 h) post implantation, followed by digestion with 1 mg/mL DNAase (Sigma) and 1 mg/mL collagenase I in Hank's Balanced Salt Solution (Solarbio) for 1 h at 37°C. Then, the red blood cells were removed by ACK lysis buffer. The infused NEs labeled with CellTrace Far Red were analyzed by flow cytometry. WT mice were used as controls.

To better understand the distribution of *meta*-NEs in lung tissues, DiD labeled NEs formulations (1.1×10^7 cells/mice) were intravenously injected into mCherry-4T1 lung metastasis mice, respectively. At 12 h post-injection, the lungs were harvested, fixed, dehydrated and sectioned. Lung sections were stained with dihydrochloride (DAPI), and observed using CLSM. For the full field imaging, the harvested lungs of mice receiving *meta*-NEs were cleared with tissue clearing reagent (PEGASOS), scanned with CLSM and analyzed by Imaris.

In vivo biodistribution of *meta*-NEs

The *in vivo* biodistribution was firstly evaluated in 4T1 lung metastasis model. Free DiR, DiR labeled NEs/Abraxane (1.1×10^7 cells/mice) or DiR labeled *meta*-NEs (1.1×10^7 cells/mice) were intravenously injected into the 4T1-Luci lung metastasis-bearing mice, respectively. After injection for 12 h, the mice were sacrificed and the organs were harvested for *ex vivo* imaging by the IVIS Spectrum Imaging System (PerkinElmer). The fluorescence intensity of the DiR signal was analyzed using Living Image Software. Besides, Abraxane (2.5 mg/kg PTX), NEs/Abraxane (1.1×10^7 cells/mice, 2.5 mg/kg PTX) or *meta*-NEs (1.1×10^7 cells/mice, 2.5 mg/kg PTX) were intravenously injected into the 4T1 lung metastasis-bearing mice, respectively. At 12 h post-injection, the organs including heart, liver, spleen, lung and spleen were harvested. The weighed organs were homogenized in saline, mixed with acetonitrile and vortexed. The amount of PTX extracted in the supernatant was quantified by HPLC. WT mice were used as controls.

In the spontaneous lung metastasis-bearing mice, DiR labeled NEs/Abraxane (1.1×10^7 cells/mice) or DiR labeled *meta*-NEs (1.1×10^7 cells/mice) were intravenously injected. After injection for different time (4, 8, 12, 24 and 48 h), the mice were sacrificed. The primary tumors and lungs were harvested for *ex vivo* imaging by the IVIS Spectrum Imaging System (PerkinElmer). The fluorescence intensity of the DiR signal was analyzed using Living Image Software. Moreover, *in vivo* biodistribution of PTX delivered by different formulations of Abraxane [Abraxane (2.5 mg/kg PTX), NEs/Abraxane (1.1×10^7 cells/mice, 2.5 mg/kg PTX) or *meta*-NEs (1.1×10^7 cells/mice, 2.5 mg/kg PTX)] was determined by quantifying the amount of PTX in tumors and lungs.

Anti-tumor efficacy and safety of *meta*-NEs

For 4T1 lung metastasis model, these tumor-bearing mice were randomly divided into six groups (n = 5 mice/group) and administrated with saline, NEs (1.1×10^7 cells/mice), ultra-NEs (1.1×10^7 cells/mice), Abraxane (2.5 mg/kg PTX), NEs/Abraxane (1.1×10^7 cells/mice, 2.5 mg/kg PTX) and *meta*-NEs (1.1×10^7 cells/mice, 2.5 mg/kg PTX) every two days for five times, respectively. The body weights of all mice were monitored during treatment. At Day23, after pulmonary function was analyzed using a whole-body plethysmography system, the mice were sacrificed, whose lungs were harvested and imaged to observe the luciferase bioluminescence using *ex vivo* IVIS Spectrum. Then, the lung tissues were fixed in 4% PFA overnight at 4°C and divided into sections (5 μm thickness) using paraffin method. The sections were stained with H&E, ki67 or TUNEL, followed by observation via dotSlide virtual microscopy (Olympus, Japan). The relative metastatic area, percentage of TUNEL and ki67 were calculated by ImageJ. The other organs including heart, liver, spleen and kidney were collected and weighed to calculate the organ index. After that, these organs were dissected, embedded in paraffin, sectioned, and stained with H&E, followed by observation using dotSlide virtual microscopy (Olympus, Japan). The blood samples were also harvested and detected by a whole blood analyzer. Meanwhile, the serum samples were collected and analyzed for evaluation of ALT, AST, ALP, BUN and CRE using per kit instructions and analyzed for evaluation of IFNγ and GzmB by ELISA. Additionally, another six mice in above treatment group were monitored to record the survival periods during treatment. For spontaneous 4T1 lung metastasis model, these tumor-bearing mice were randomly divided into six groups (n = 5 mice/group) and receiving the same treatment as above. The volume of primary tumor was measured by vernier caliper every 2 days. At Day 32, mice were sacrificed, whose lung were harvested and imaged to observe the luciferase bioluminescence using IVIS Spectrum. Another five mice in above treatment group were monitored for survival evaluation.

For *in situ* resection model with spontaneous 4T1 lung metastasis, mice were randomly divided into six groups (n = 5 mice/group) at Day 20 and receiving the same treatment as above. The volume of primary tumor was measured by vernier caliper every 2 days for evaluation of the tumor recurrence. At Day 32, mice were sacrificed, whose lung were harvested and and imaged to observe the luciferase bioluminescence using IVIS Spectrum. Another five mice in above treatment group were monitored for survival evaluation.

For humanized TNBC mouse model, female NSFG mice were intravenously injected with 1×10^6 PBMCs per mouse via the tail vein. Subsequently, MDA-MB-231 cells were inoculated into the right mammary fat pad of the mice to establish the humanized TNBC mouse model. Mice were randomly divided into four groups with the following treatments: Saline, NEs (7×10^6 cells/mice), ultra-NEs (7×10^6 cells/mice), and *meta*-NEs (7×10^6 cells/mice, 2.5 mg/kg PTX). Administration was performed once every 2 days for a total of 3 treatments. Animal survival was monitored daily, with the endpoint defined as a subcutaneous tumor volume exceeding 1500 mm^3 or the onset of a moribund state necessitating euthanasia. Survival data were statistically analyzed using the Kaplan-Meier method. Tumor volume was assessed on alternate days using caliper measurements; the length (L, major axis) and width (W, minor perpendicular axis) were used to calculate volume (V) according to the formula: $V = (L \times W^2)/2$. Individual body weights were recorded every other day to evaluate overall health and potential treatment toxicity. A sustained body weight loss exceeding 15% from the initial baseline was established as a criterion indicative of significant toxicity. All mice were humanely euthanized on experimental day 38. Terminal blood samples were collected via orbital venous plexus blood collection. Serum, separated by centrifugation, was analyzed using commercially available ELISA kits to quantify circulating levels of the IL-6 and IL-10 according to the manufacturers'protocols. A complete necropsy was performed, with the lungs excised for macroscopic examination. Visible metastatic foci on the lung surfaces measuring $\geq$1 mm^2 were systematically identified and enumerated to quantify pulmonary metastatic burden.

Evaluation the tumoricidal and immunoregulatory potency of *meta*-NEs *in vitro*

To determine the expression of CD80, CD86 and ICAM-1, NEs, ultra-NEs and *meta*-NEs were stained with anti-mouse CD80 antibody, anti-mouse CD86 antibody and anti-mouse icam-1 antibody, respectively. The fluorescence intensity was determined by using flow cytometry.

For evaluating the immunoregulatory effect of *meta*-NEs on $CD8^+$T cell *in vitro*, splenic $CD8^+$T cells from WT BALB/c mice were isolated by the EasySep Mouse $CD8^+$T cell Isolation Kit (StemCell) as pervious report.[51] The proliferation of $CD8^+$T cells were detected by the CFSE assay following the manufacturer's instructions (Beyotime). $CD8^+$T cells (1×10^5 cells/well) were co-cultured with NEs, ultra-NEs or *meta*-NEs in a ratio of 1:2 in a 96-well non-treated round bottom plate (Corning) with pre-coated Anti-CD3 (5 μg/mL, BioGems) and anti-CD28 antibodies (2 μg/mL, BioGems). After 72 h, the cells were collected and staining with anti-mouse CD8a antibody, anti-mouse Ly6G antibody at 4°C for 15 min. After washing with PBS twice, the proliferation of $CD8^+$T cells was evaluated by flow cytometry. For determination of the expressions of effector molecules in $CD8^+$T cells, $CD8^+$T cells were collected as described above and incubated with 1 μM ionomycin and 50 ng/mL PMA for 4 h in the presence of 5 μg/mL brefeldin A. Then, the cells were collected and stained with 1 μg/mL PE-Cy7 anti-mouse CD8a at 4°C for 30 min, followed by fixing with 4% PFA and permeabilization using 0.1% Triton X-100. After that, the cells were stained with anti-mouse GzmB recombinant antibody and anti-mouse TNFα antibody for 30 min at 4°C, and analyzed by flow cytometry.

To determine the synergistic effect between Abraxane-mediated cytotoxicity and NEs-induced immune activation, the temporal synergy was systematically evaluated using a Transwell co-culture system. The experiment was divided into four groups. For the experimental group, T cells and *meta*-NEs were added to the upper chamber of the Transwell system, and tumor cells were placed in the lower chamber. Three control groups were set as follows: the first control group contained T cells in the upper chamber and tumor cells in the lower chamber; the second control group contained ultra-NEs in the upper chamber and tumor cells in the lower

chamber; the third control group contained T cells and ultra-NEs in the upper chamber and tumor cells in the lower chamber. A total of 1 mL of cell suspension at a density of 1×10^6 cells/mL was added to the upper chamber of the Transwell chemotaxis system, and tumor cell medium containing 10% FBS was added to the lower chamber. The Transwell plate was incubated in a 37°C, 5% CO_2 incubator for 12 h. The lower-chamber tumor cells were collected, and the intracellular FITC fluorescence intensity of the drug and tumor cell viability were analyzed by flow cytometry. The upper-chamber cells were collected, and the secretion of inflammatory factors in T cells was detected by flow cytometry.

Evaluation the tumoricidal and immunoregulatory potency of *meta*-NEs *in vivo*

After injection of DiD-labeled NEs formulations (1.1×10^7 cells/mice) into established or spontaneous lung metastasis-bearing mice for 12 h, respectively, mice were euthanized. Lungs were harvested, shredded and digested with 1 mg/mL DNAase (Sigma) and 1 mg/mL collagenase I in Hank's Balanced Salt Solution (Solarbio) for 1 h at 37°C. After removing the red blood cells by ACK lysis buffer, the collected cells were purified by Percoll density gradient centrifugation as mentioned above and stained with Ly6G antibody. Reinfused NEs were sorted as $Ly6G^+DiD^+$ by fluorescence-activated cell sorting (BD FACS Aria II). The cells were washed with ice-cold PBS twice and analyzed by RT-qPCR as mentioned above. To measure the expression of costimulatory molecules of reinfused NEs, the $Ly6G^+DiD^+$ NEs were stained with anti-mouse CD45 antibody, anti-mouse Ly6G antibody, anti-mouse CD80 antibody, anti-mouse CD86 antibody and anti-mouse icam-1 antibody, followed by analysis using flow cytometry. The expressions of RNS and ROS in the $Ly6G^+DiD^+$ NEs were measured using DAF-FM DA and an Oxidative stress detection kit, respectively, according to the manufacturer's instructions.

For evaluation of the immunomodulating ability *in vivo*, the secretion levels of TNFα and GzmB in tumor tissue (lung) were first determined by ELISA. Moreover, leukocytes in the isolated lungs and orthotopic tumors were isolated by 40–70% percoll gradient centrifugation. To investigate the infiltration of immune cells in lungs with metastases, the isolated leukocytes were stained with anti-mouse CD45 antibody, anti-mouse CD8a, anti-mouse CD49b, anti-mouse F480, and anti-mouse CD11c at 4°C for 30 min, followed by analysis using flow cytometry. The anti-tumor response of tumor-infiltrated $CD8^+$T cells were explored using the same method *in vitro* except for using the isolated leukocytes. The anti-tumor response of tumor-infiltrated NK cells was explored by staining the isolated leukocytes with anti-mouse CD45 antibody, anti-mouse CD49b antibody, anti-mouse GzmB antibody, anti-mouse IFNγ antibody and anti-mouse TNFα antibody at 4°C for 30 min, and analyzed by flow cytometry. To measure the functions of tumor-infiltration DCs, the isolated leukocytes were stained with anti-mouse CD45 antibody, anti-mouse CD11c, anti-mouse CD80 antibody, anti-mouse CD86 antibody at 4°C for 30 min, and analyzed by flow cytometry.

QUANTIFICATION AND STATISTICAL ANALYSIS

Statistical analyses were performed using GraphPad Prism 10.1.2. All plots show mean ± SEM. A one-way ANOVA test and two-way ANOVA with Tukey's correction was used for comparisons of multiple groups and a Student's unpaired *t* test was used for two-group comparisons in the appropriate conditions. A log rank (Mantel-Cox) test was used to analyze survival differences. Statistical significance was set at *$p < 0.05$, **$p < 0.01$, ***$p < 0.001$, and ****$p < 0.0001$. n.s: no significant difference.

Cell Reports Medicine, Volume *7*

Supplemental information

Non-genetically reprogrammed meta-neutrophils potentiate chemo-immunotherapy against lung metastatic triple-negative breast cancer

Meixi Hao, Qifan Hu, Xiuqi Li, Yijun Chen, Siyuan Hou, Chunjing Han, Sijia Chen, Yanyi Li, Kaiming Li, Lingjing Xue, Lulu Zhu, Shanshan Chen, Caoyun Ju, and Can Zhang

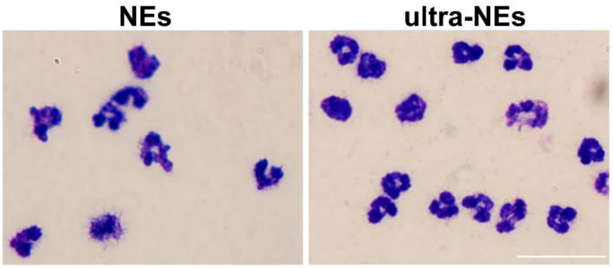

Figure S1. Assessment of NEs Nuclear Morphology Following IFNγ Treatment by Wright-Giemsa Staining. Related to Figure 1. Sclar Bar: 25 µm.

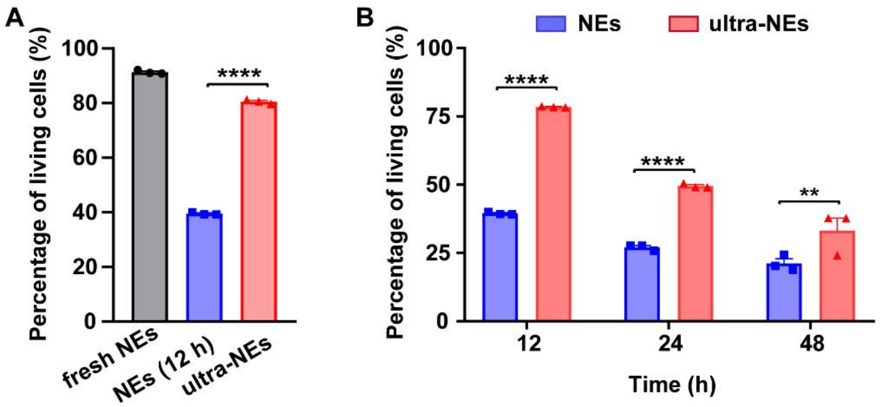

Figure S2. Cell viability of NEs from healthy donors after trained with IFNγ. (A) Cell viability of NEs after IFNγ treated for 12 h. (B) Cell viability of NEs after IFNγ treated for different time. Related to Figure 1. Data were shown as mean ± SEM. n = 3 independent samples, ****P<0.0001, **P<0.01.

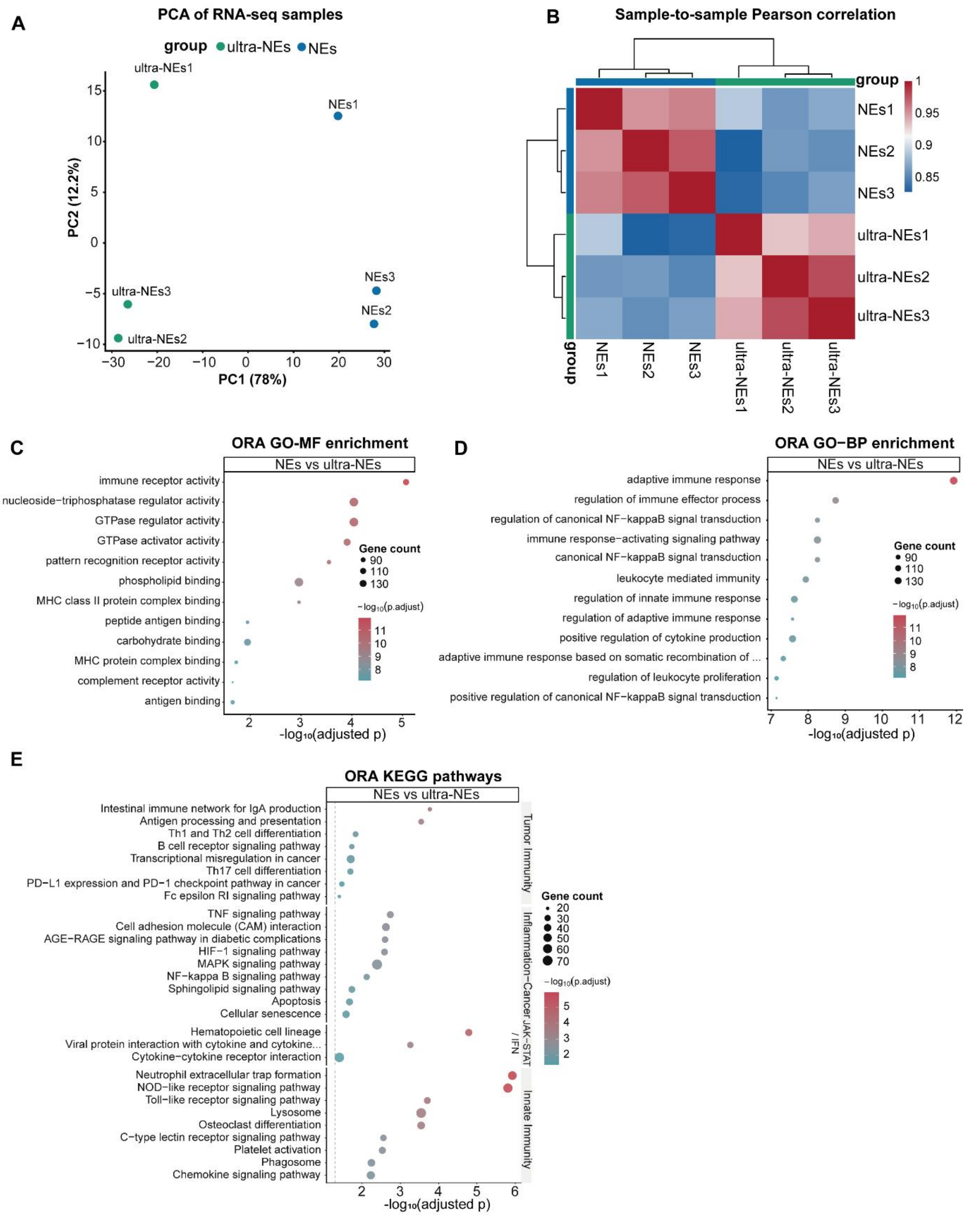

Figure S3. Enhanced RNA-seq analysis overview. (A) Principal component analysis (PCA). (B) Hierarchical clustering analysis. (C) GO-MF enrichment analysis. (D) GO-BP enrichment analysis. (E) KEGG pathway analysis. Related to Figure 1.

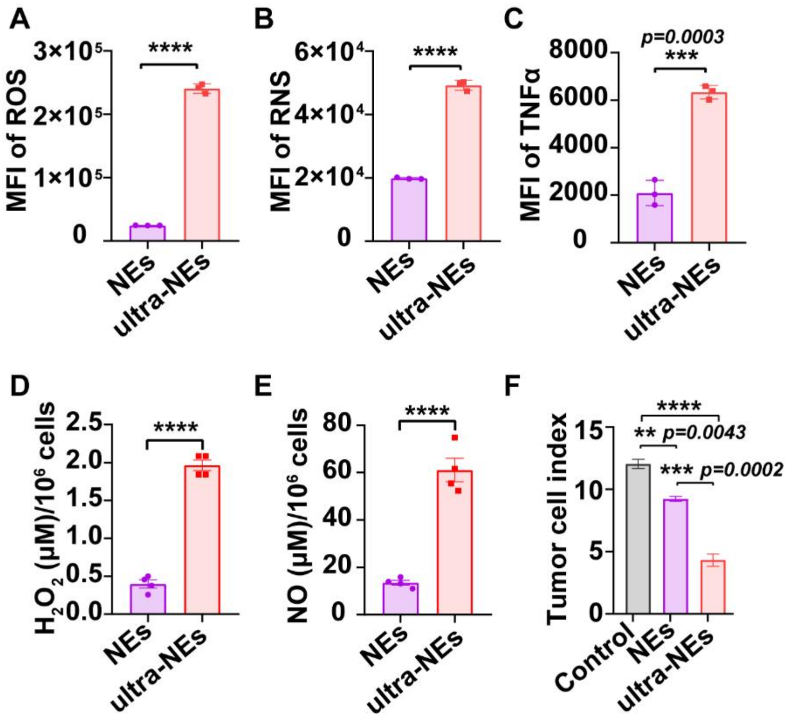

Figure S4. Toxic effector molecules expression and tumor cell index of ultra-NEs. Quantification of ROS (A), RNS (B), TNFα (C), hydrogen peroxide (H2O2, D), and nitric oxide (NO, E) secreted by ultra-NEs. H2O2 is one of the important effector molecules of ROS. NO is the main source of RNS. (F) Quantification of tumor cell index. Related to Figure 1. Data were analyzed by two-tailed Student's t test (A-E), or one-way ANOVA test with Tukey's correction (F). All data were shown as mean ± SEM (n = 4 samples per group), ****P<0.0001.

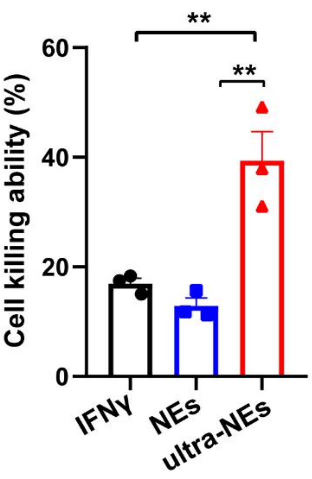

Figure S5. The tumor-killing ability of IFNγ and ultra-NEs. Related to Figure 1. Data were analyzed by one-way ANOVA test with Tukey's correction and shown as mean ± SEM (n = 3 samples per group). ***P*<0.005.

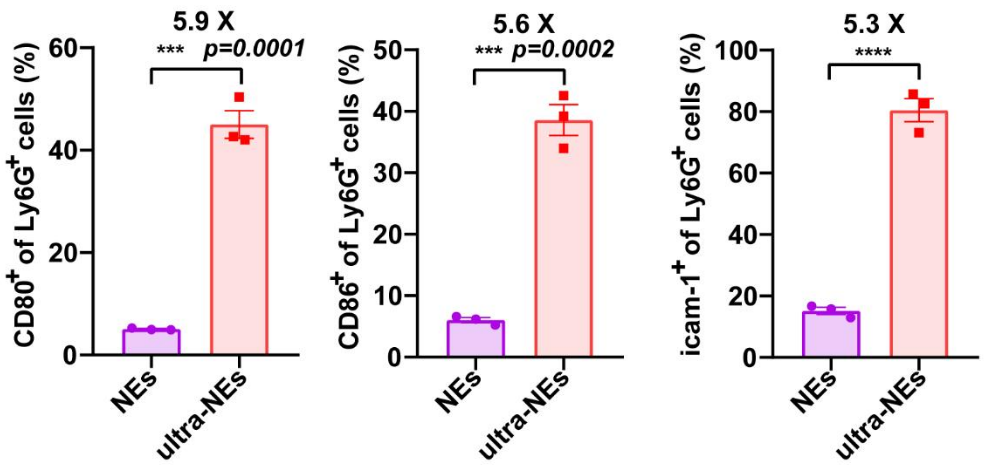

Figure S6. Quantification of the expressions of CD80, CD86 or icam-1 in ultra-NEs. Related to Figure 1. Data were analyzed by two-tailed Student's t test and shown as mean ± SEM (n = 4 samples per group). ****P<0.0001.

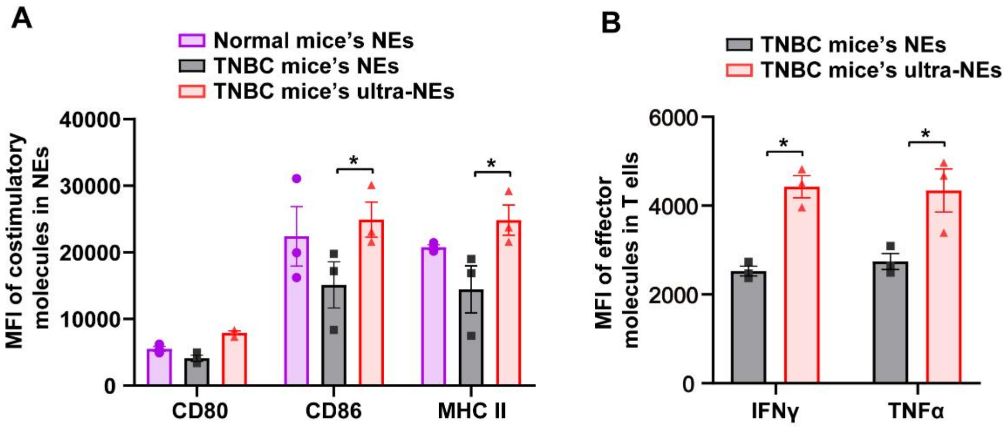

Figure S7. The antigen-presenting and T cell activation ability of NEs derived from tumor-bearing mice after IFNγ training. (A) Quantification of the expressions of CD80, CD86 or MHC II in ultra-NEs derived from tumor-bearing mice. (B) Quantification of TNFα and IFNγ expression of tumor-infiltrating $CD8^{+}$T cells co-cultured with ultra-NEs derived from tumor-bearing mice. Related to Figure 2. Data were analyzed by one-way ANOVA test with Tukey's correction and shown as mean ± SEM (n = 3 samples per group). $*P<0.01$.

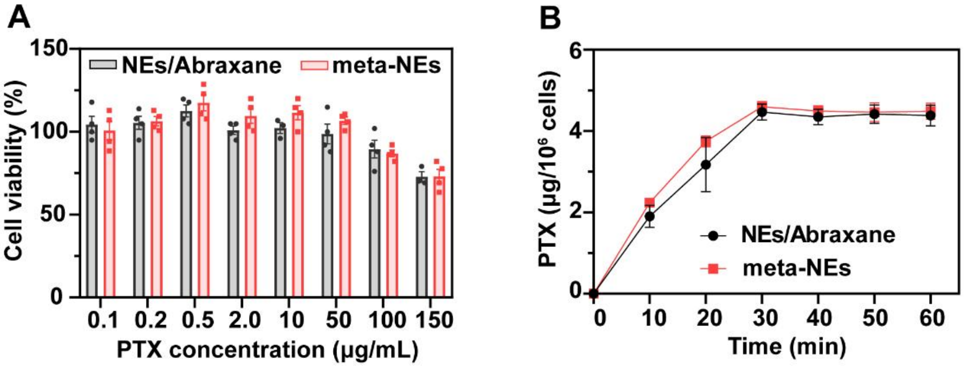

Figure S8. Screening of the concentration and incubation time of Abraxane. (A) In vitro cytotoxicity of different concentration of Abraxane on ultra-NEs or NEs for 12 hours (n = 4 independent samples). (B) Loading efficiency at different time of NEs/Abraxane and meta-NEs. n= 3 samples per group. Related to Figure 3. Data were shown as mean ± SEM.

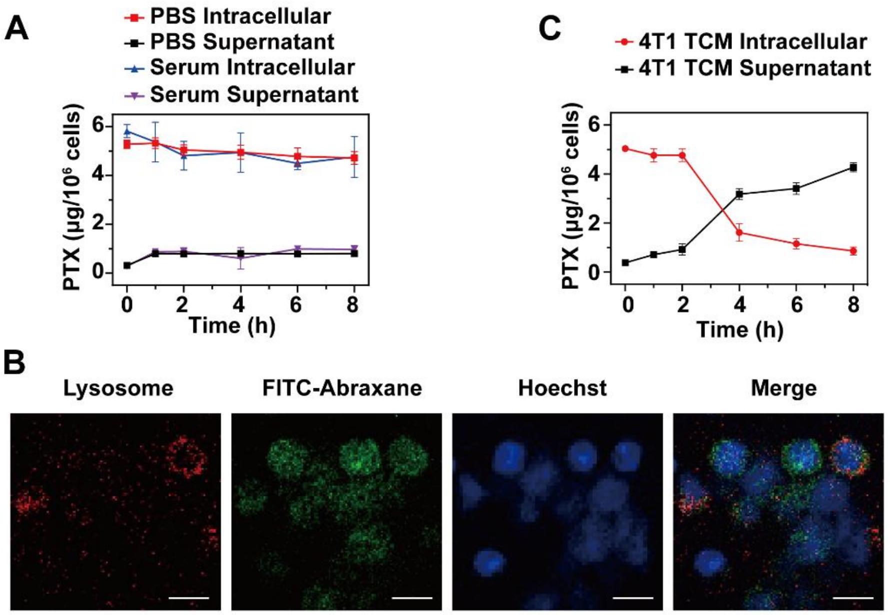

Figure S9. Stability and drug release of meta-NEs. (A) In vitro stability of meta-NEs after incubation with PBS or 50% serum over time (n = 3 samples per group). (B) The confocal images of the uptake pathway of meta-NEs. Abraxane were labeled with FITC (Green), lysosomes were labeled with LysoTracker Red DND-99 (Red), and cell nuclei were labeled with Hoechst 33342 (Blue). Scale bar: 20 μm. (C) Release of PTX from meta-NEs after incubation with 4T1 TCM over time (n = 3 samples per group). Related to Figure 3.

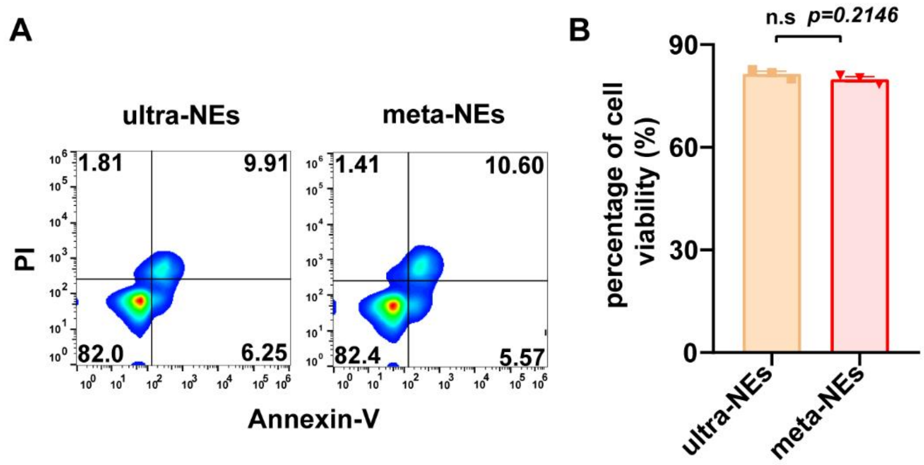

Figure S10. Cell viability of meta-NEs. (A) Representative image of apoptosis of ultra-NEs before and after drug loading determined by flow cytometry. (B) Quantification of cell viability (n = 3 samples per group). Related to Figure 3. Data were analyzed by two-tailed Student's t test and shown as mean ± SEM. n.s denotes no significant difference.

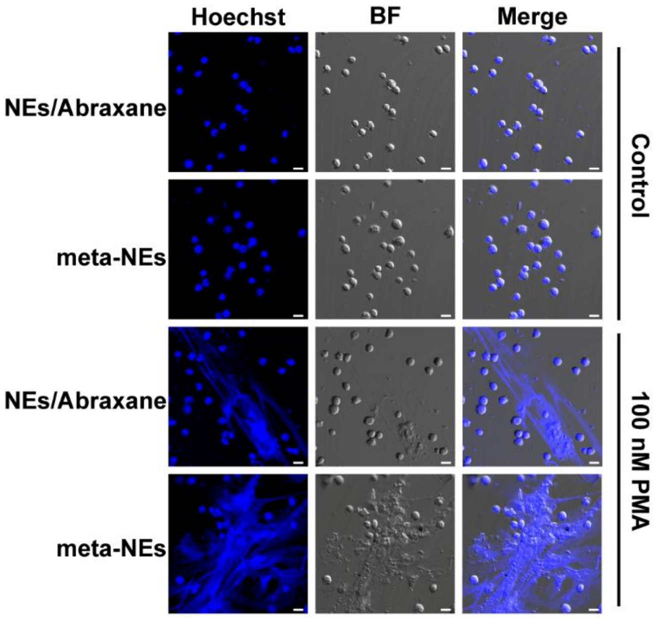

Figure S11. Representative image of the release of NETs from meta-NEs after treatment with 100 nM PMA for 4 hours in vitro. Scale bars, 10 μm. Related to Figure 3.

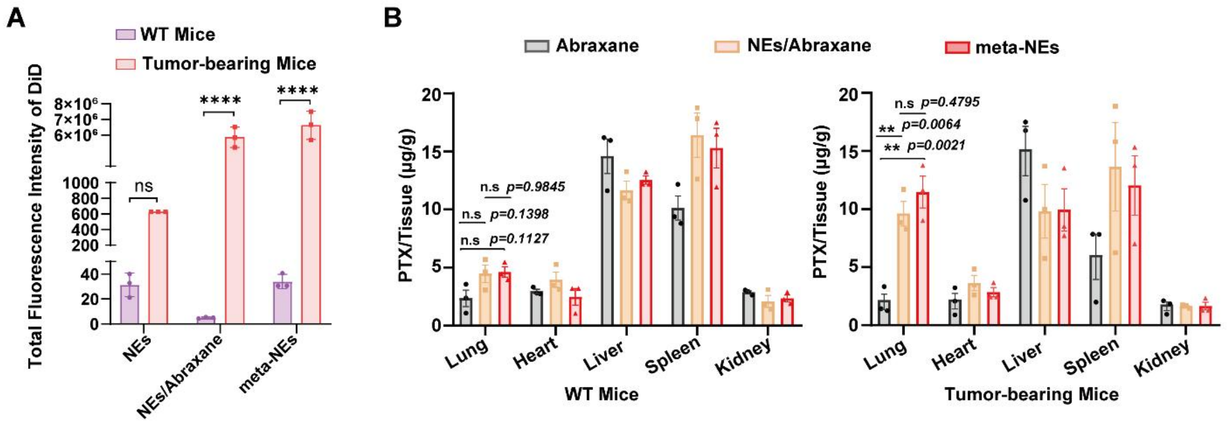

Figure S12. Distribution of NEs and PTX in WT mice and tumor-bearing mice. (A) Quantitative analysis of DiD fluorescence in Figure 3I (n = 3 mice per group). (B) Amounts of PTX amounts in different tissues harvested from WT mice and 4T1-Luci-bearing mice after intravenous injection of different Abraxane formulations for 12 hours (n = 3 mice per group). Related to Figure 3.

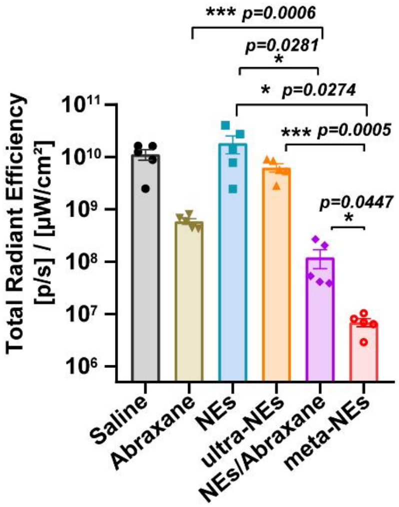

Figure S13. Total radiant efficiency of lung bioluminescence from the tumor-bearing mice after treatment (N= 5 mice per group). Related to Figure 4. Data were shown as the mean ± SEM and analyzed by one-way ANOVA test with Tukey's correction.

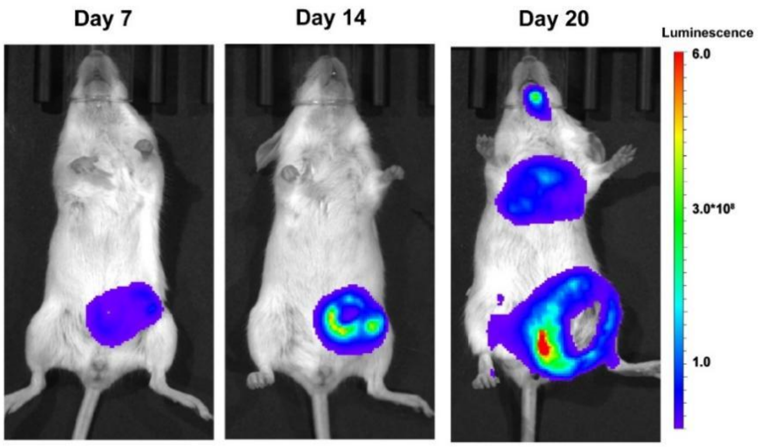

Figure S14. The bioluminescence images of spontaneous TNBC lung metastasis mouse model. Related to Figure 4.

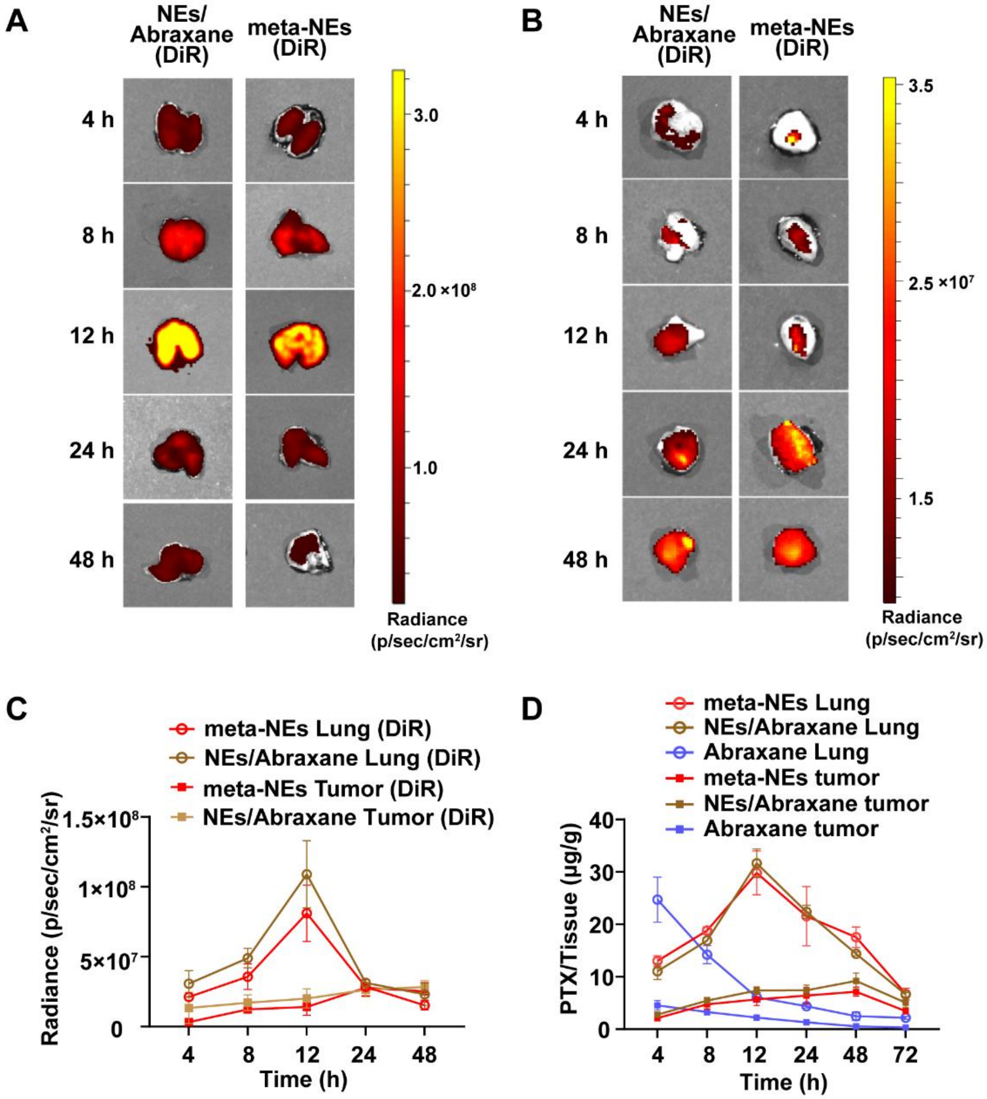

Figure S15. Distribution of meta-NEs in primary tumor and lung metastasis foci. Ex vivo images of the lung metastasis loci (A) and primary tumor (B) collected from mice receiving DiR-labeled meta-NEs and NEs/Abraxane at 4, 8, 12, 24 and 48-hours post-injection. (C) The fluorescent quantification of A) and B). (D) Quantification of PTX in primary tumor and lung metastatic foci after intravenous injection of different Abraxane formulations with the dosage of PTX at 2.5 mg/kg. n = 3 mice per group. Error bars denote SEM. Related to Figure 4.

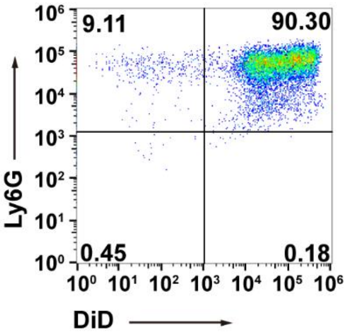

Figure S16. Purity of meta-NEs sorted from lungs of 4T1 metastasis bearing mice. Meta-NEs were labeled with DiD before intravenous injection. Related to Figure 5.

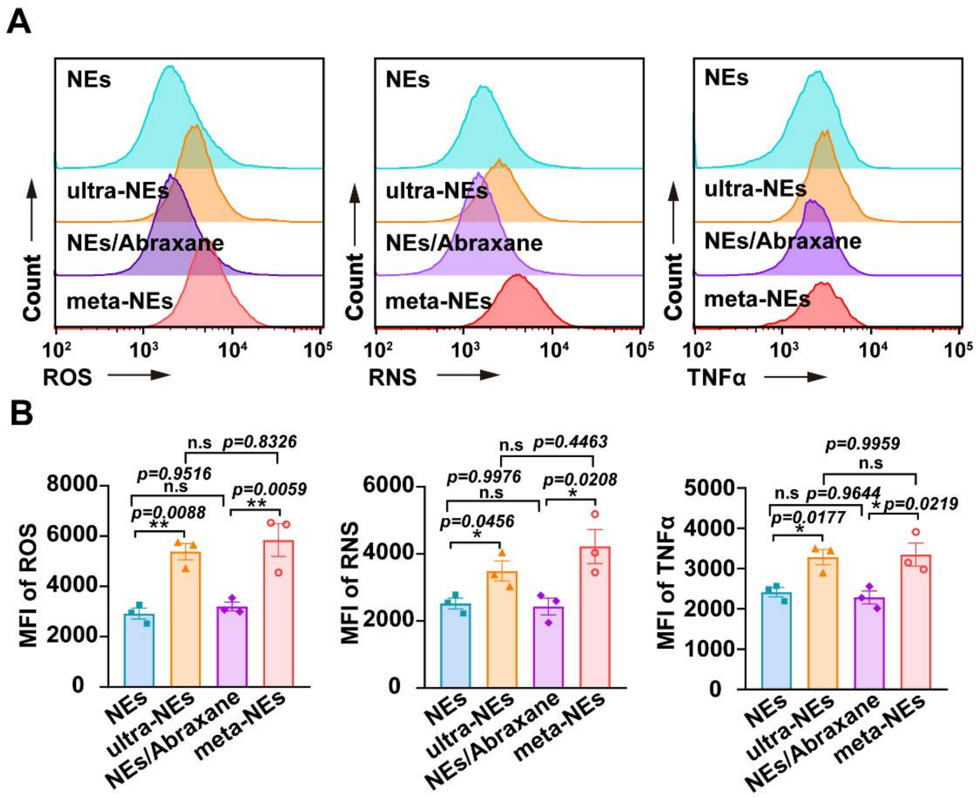

Figure 17. Flow cytometry analysis of ROS, RNS, and TNFα expression of reinfused NEs in lung metastatic foci from spontaneous TNBC lung metastasis mouse model. (A) Representative flow cytometry analysis of ROS, RNS, and TNFα expression. (B) Quantification of ROS, RNS, and TNFα expression. n = 3 mice per group. Related to Figure 5. Data were analyzed by one-way ANOVA test with Tukey's correction and shown as the mean ± SEM. n.s denotes no significant difference.

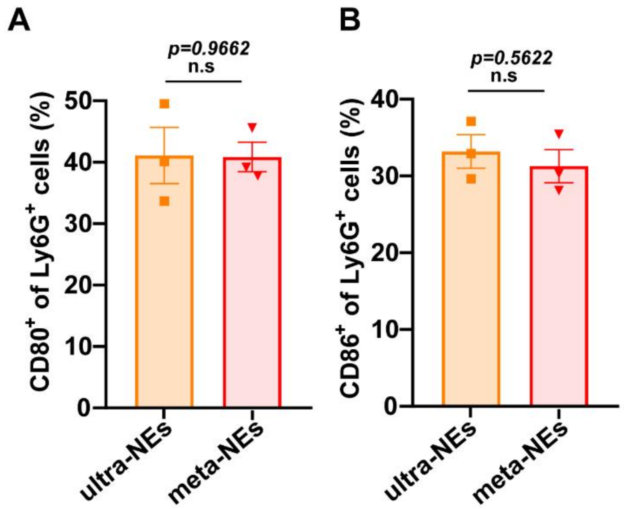

Figure S18. Quantification of co-stimulation molecules of meta-NEs. (A) Quantification of CD80 expression of meta-NEs. (B) Quantification of CD86 expression of meta-NEs. Ultra-NEs were used as a control. Related to Figure 6. Data were analyzed by two-tailed Student's t test and shown as mean ± SEM (n = 3 samples per group). n.s denotes no significant difference.

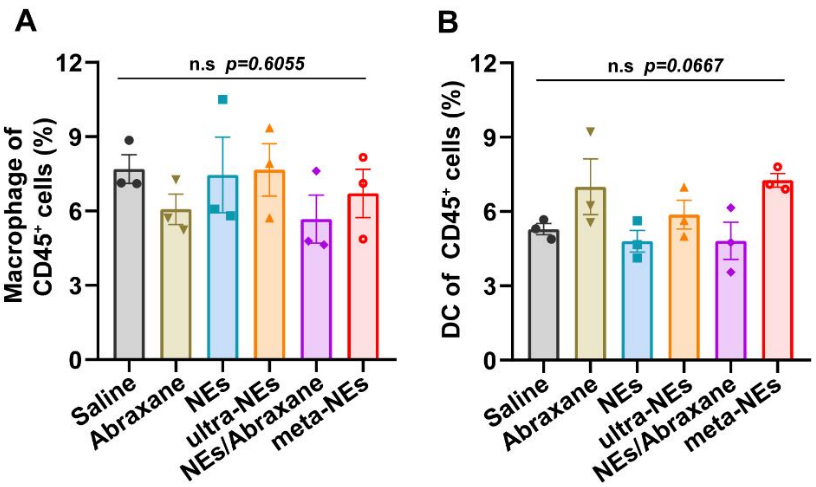

Figure S19. The proportion of macrophage and DCs in lung metastatic foci. Related to Figure 6. Data were analyzed by one-way ANOVA test with Tukey's correction and shown as mean ± SEM (n = 3 mice per group). n.s denotes no significant difference.

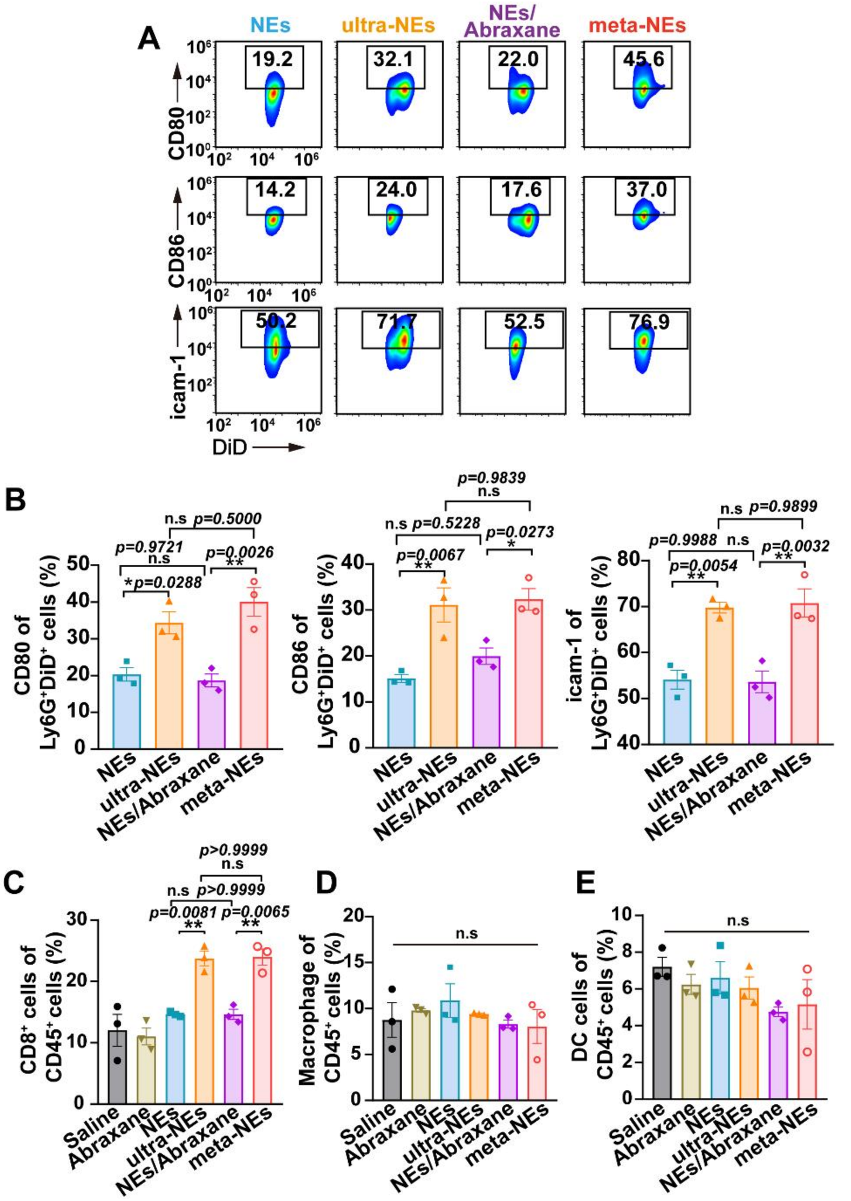

Figure S20. The expressions of CD80, CD86, and icam-1 of reinfused NEs and the frequency of infiltrated immune cells in lungs metastatic foci from spontaneous TNBC lung metastasis bearing mice. (A) Representative flow cytometry analysis of CD80, CD86, and icam-1 expression. (B) Quantification of CD80, CD86, and icam-1 expression. (C-E) The frequency of infiltrated $CD8^{+}$T cells (C), macrophages (D), and DCs (E) in spontaneous lung metastatic foci. n = 3 mice per group. Related to Figure 6. Data were analyzed by one-way ANOVA test with Tukey's correction and shown as the mean ± SEM. n.s denotes no significant difference.

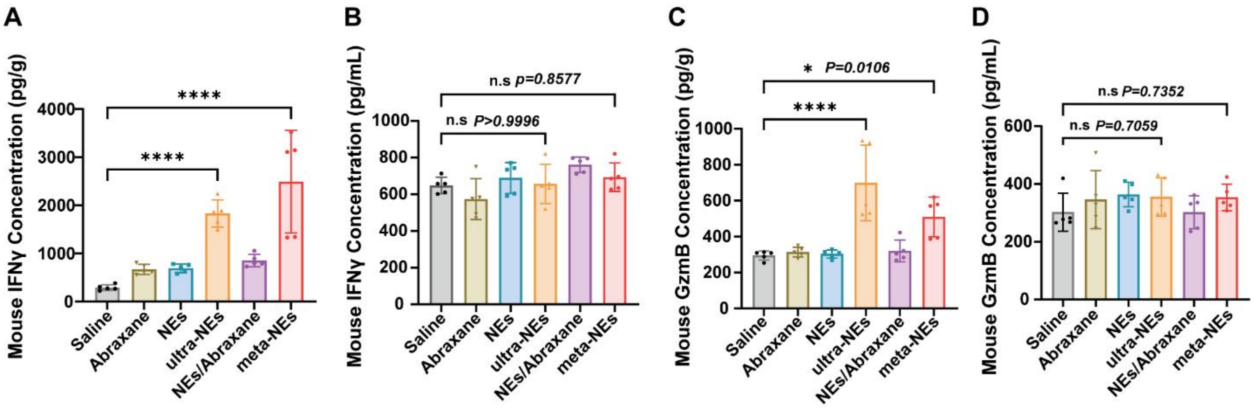

Figure S21. ELISA analysis of secreted cytokine levels in peripheral blood and tumor tissues of mice (n = 5 mice per group). (A) IFNγ levels in tumor tissues. (B) IFNγ levels in serum. (C) GZMB levels in tumor tissues. (D) GZMB levels in serum. Related to Figure 6. Data were analyzed by one-way ANOVA test with Tukey's correction and shown as the mean ± SEM. ****$P < 0.0001$, n.s denotes no significant difference.

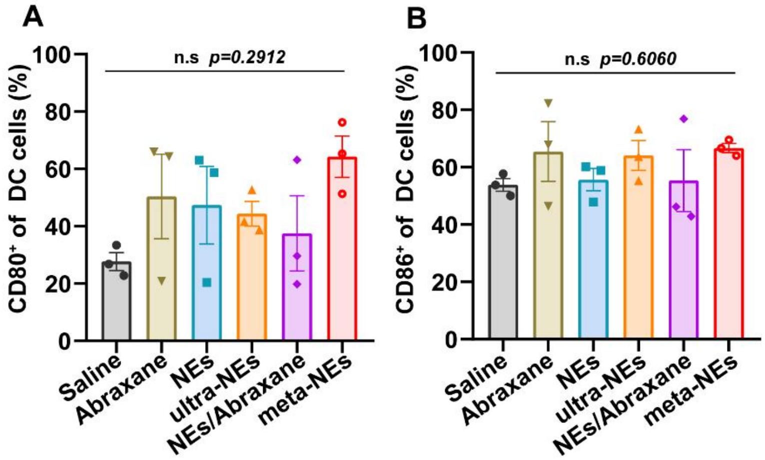

Figure S22. Expressions of CD80 and CD86 of DCs in lung metastatic foci. Related to Figure 6. Data were analyzed by one-way ANOVA test with Tukey's correction and shown as mean ± SEM (n = 3 mice per group). n.s denotes no significant difference.

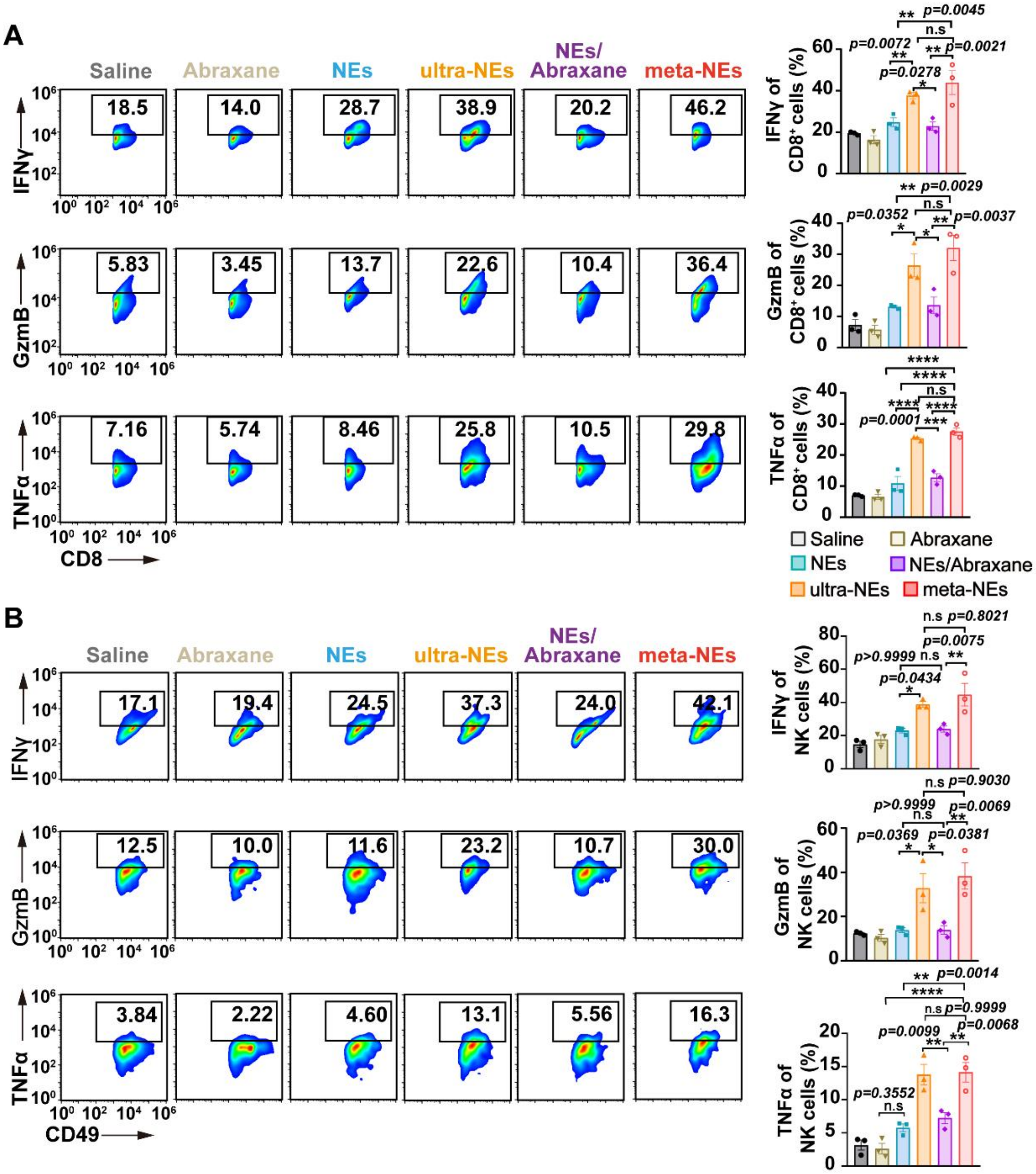

Figure S23. Flow cytometry analysis of IFNγ, GzmB and TNFα levels of tumor-infiltrated CD8^{+}T cell (A) and NK cells (B) in lung metastatic foci from spontaneous TNBC lung metastasis bearing mice. n = 3 mice per group. Related to Figure 6. Data were analyzed by one-way ANOVA test with Tukey's correction and as the mean ± SEM. ****P<0.0001, n.s denotes no significant difference.

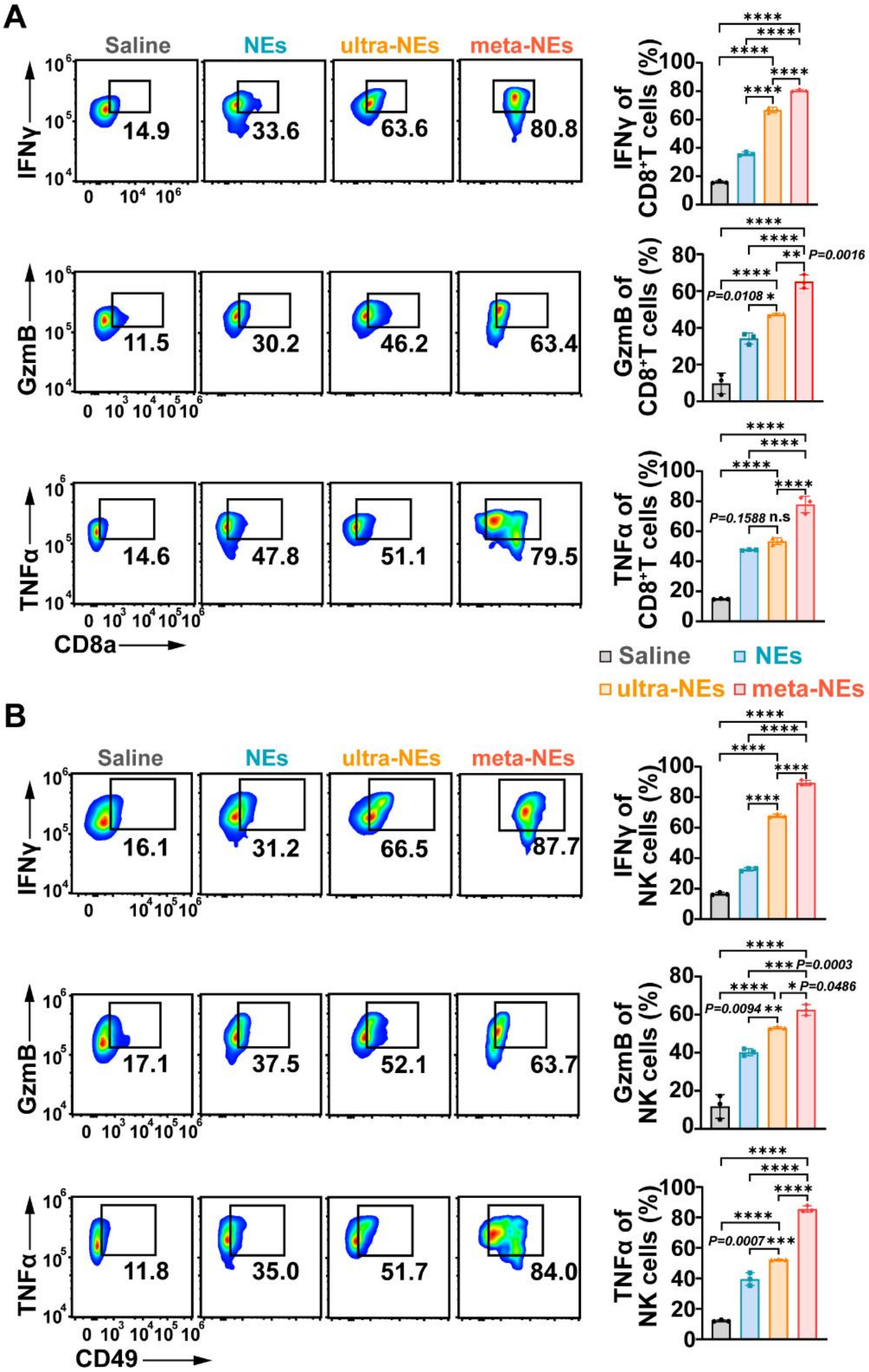

Figure S24. Flow cytometry analysis of IFNγ, GzmB and TNFα levels of tumor-infiltrated CD8^{+}T cell (A) and NK cells (B) in primary tumors from spontaneous TNBC lung metastasis bearing mice. n = 3 mice per group. Related to Figures 6. Data were analyzed by one-way ANOVA test with Tukey's correction and as the mean ± SEM. ****P<0.0001, n.s denotes no significant difference.

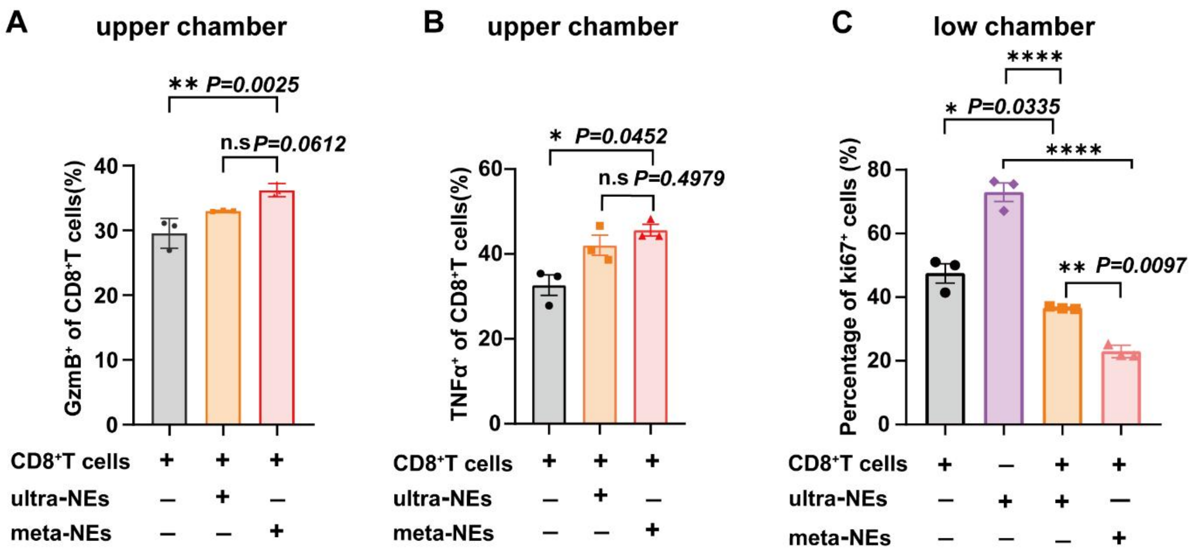

Figure S25. Transwell assay evaluating synergistic effects between NEs-mediated immune activation and Abraxane-mediated cytotoxicity. Expression of GzmB (A) and TNFα (B) in $CD8^{+}$T cells from the upper chamber. (C) Expression of ki67 in tumor cells from the lower chamber. n = 3 samples per group. Related to Figure 6. Data were analyzed by one-way ANOVA test with Tukey's correction. Error bars denote SEM. ****$P < 0.0001$, n.s denotes no significant difference.

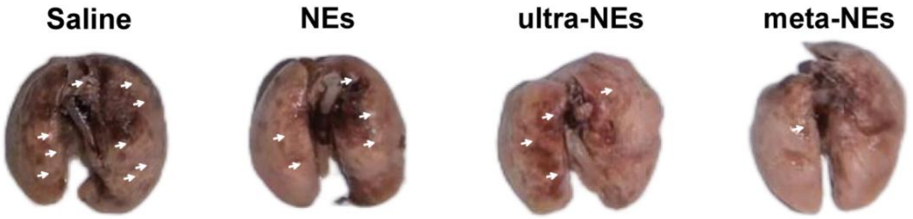

Figure S26. Representative lung metastasis images from a humanized TNBC mouse model. Related to Figure 7.

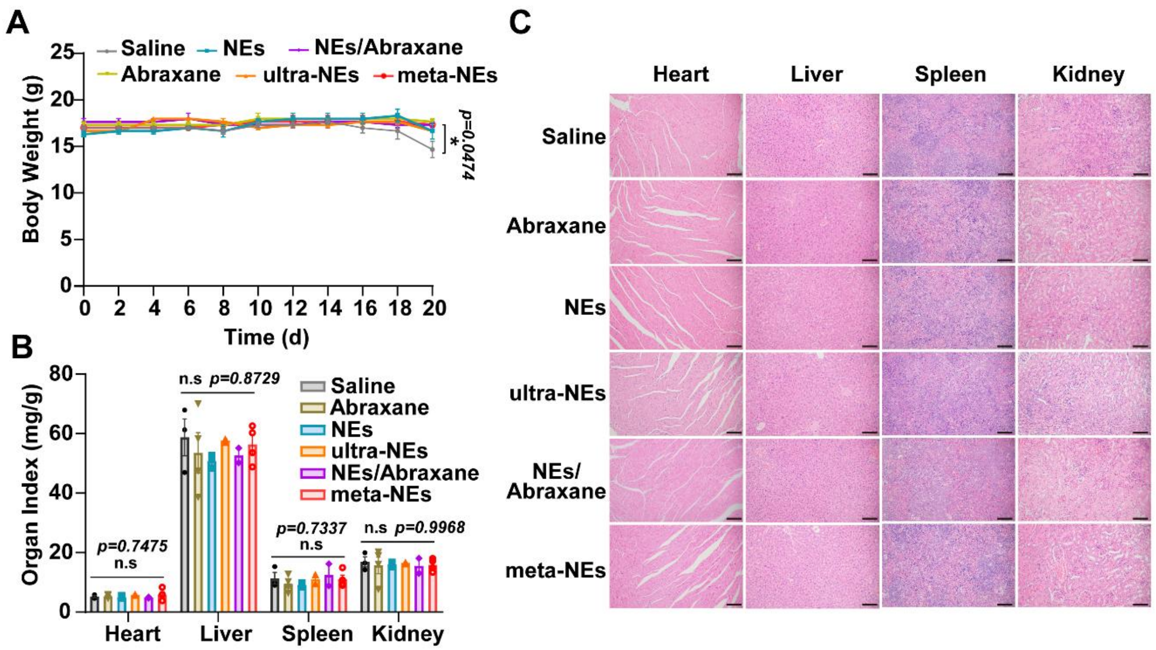

Figure S27. Evaluation of body weight, organ indexes, and H&E changes in established lung metastasis model after administration of meta-NEs. (A) Variations in body weights during the process of treatment (n = 3 mice per group). (B) Organ weight normalized to body weight (n = 4 mice per group). (C) H&E staining of tissue sections of heart, liver, spleen and kidney. Scale bar, 100 μm. Related to Figure 7. Data were analyzed by or two-tailed Student's *t* test (A) or one-way ANOVA test with Tukey's correction (B) and shown as the mean ± SEM. n.s denotes no significant difference.

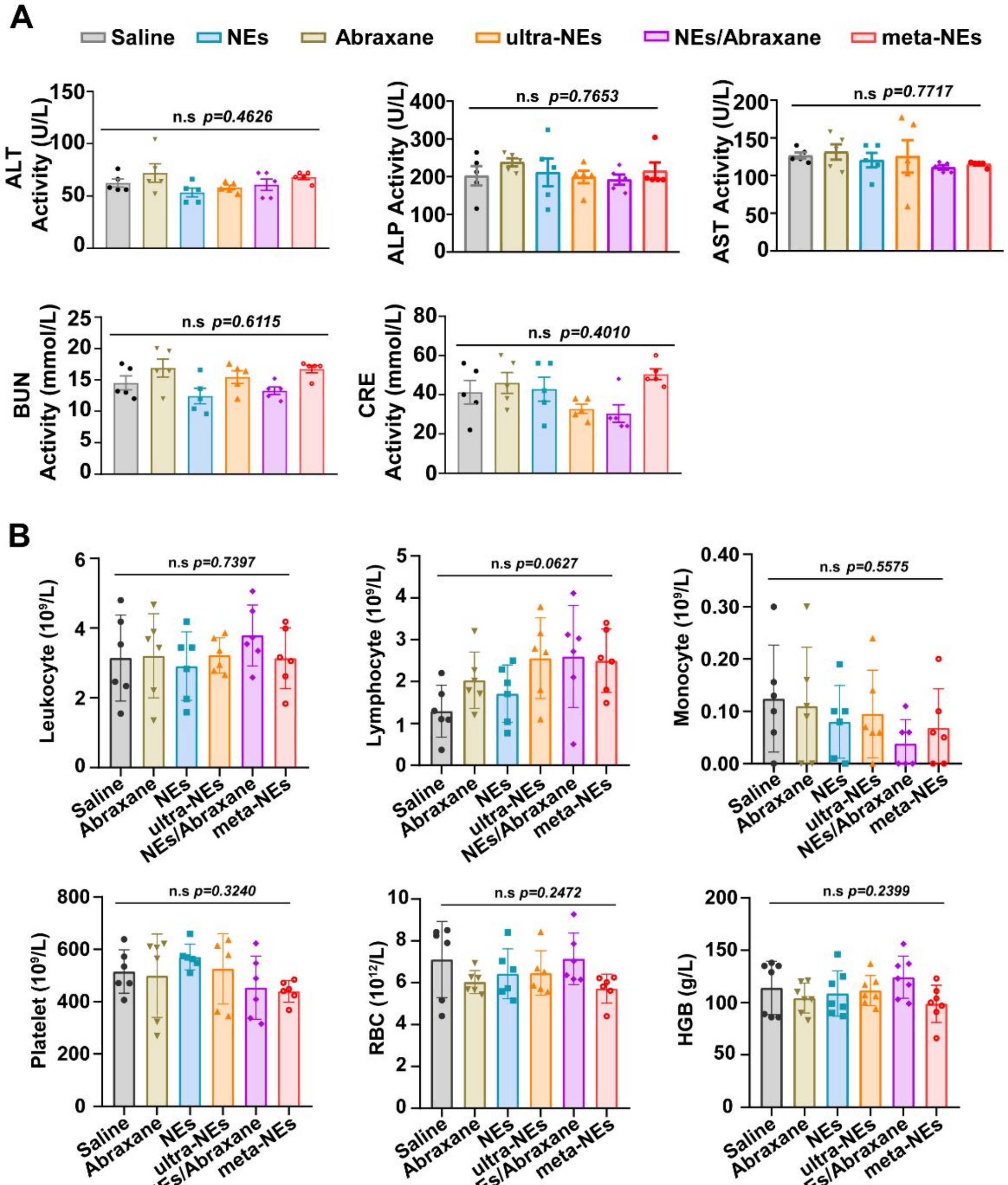

Figure S28. Blood biochemical and hematological analyses in established lung metastasis model after administration of meta-NEs. (A) Expressions of markers of liver function ALT, ALP and AST, as well as kidney function BUN and CRE (n = 5 mice per group). (B) Determination of the numbers of leukocytes, lymphocytes, monocytes, platelets and RBC, as well as the amount of HGB in blood (n = 6 mice per group). Related to Figure 7. Data were analyzed by one-way ANOVA test with Tukey's correction. Error bars denote SEM. ****$P < 0.0001$, n.s denotes no significant difference.

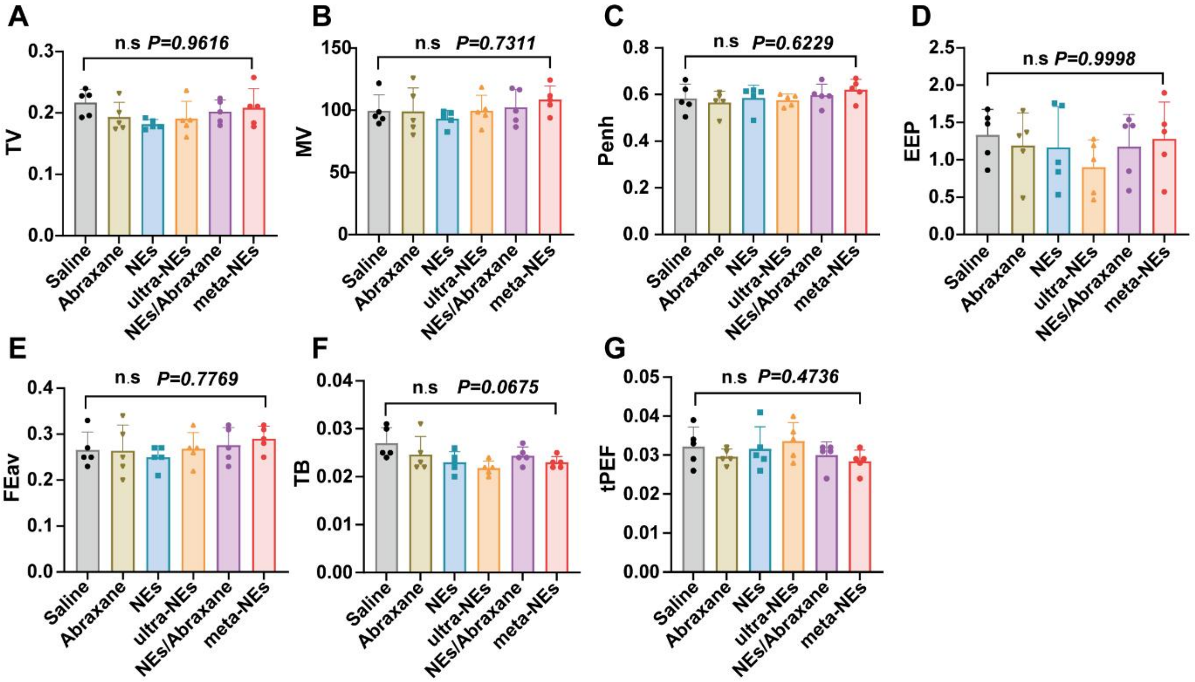

Figure S29. Pulmonary respiratory function assay via whole-body plethysmography. (A) MV-Minute Ventilation. (B) TV-Tidal Volume. (C) Penh-Airway Constriction Index. (D) EEP-End-Expiratory Pause. (E) FEav-Average Expiratory Flow. (F) TB-Airway Inflammation Index. (G) tPEF-Time to Peak Expiratory Flow. n = 5 mice per group. Related to Figure 7. Data were analyzed by one-way ANOVA test with Tukey's correction. Error bars denote SEM.*****P* < 0.0001, n.s denotes no significant difference.

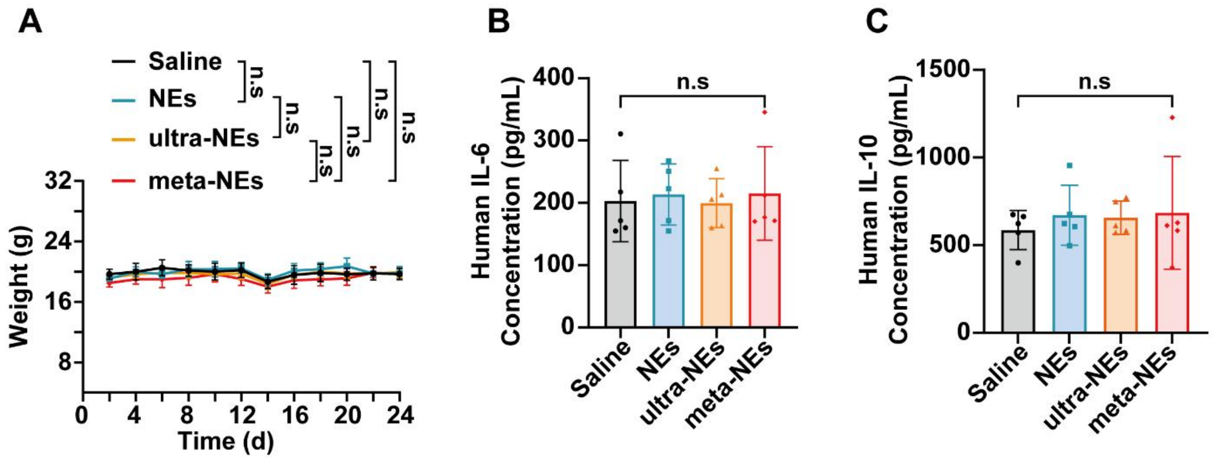

Figure S30. Evaluation of body weight and the risk of a cytokine storm in humanized TNBC mouse model after administration of meta-NEs. (A) Statistics of body weight changes in mice (n = 6 mice per group). (B-C) Levels of human IL-6 and IL-10 in humanized mouse blood after treatment (n = 5 mice per group). Related to Figure 7. Data were analyzed by one-way ANOVA test with Tukey's correction. Error bars denote SEM. n.s denotes no significant difference.

Table S1. Characteristics of the FITC-labeled Abraxane (FITC-Abraxane). PDI, polydispersity index. Related to Figure 3. Data were shown as mean ± SD (n = 3 independent experiments).

	Particle Size (nm)	PDI	Zeta potential (mV)
**Abraxane**	**148.2±1.5**	**0.13±0.07**	**-25.77±0.74**
**FITC-Abraxane**	**160.8±1.0**	**0.13±0.05**	**-21.81±1.71**

Table S2. Primer sequences for real-time PCR. Related to STAR METHODS.

Primers	Forward (5' to 3')	Reverse (5' to 3')
Actb	TGTCCACCTTCCAGCAGATGT	AGCTCAGTAACAGTCCGCCTAGA
Mmp9	TGCCCAGCGACCACAACTC	CGGACCCGAAGCGGACATT
Arg1	GACCACGGGGACCTGGCCTT	ACTGCCAGACTGTGGTCTCCACC
Prok2	TGCTACTTCTGCTGCTACC	CCGCACTGAGAGTCTTGTC
Ccl2	CGGCTGGAGCATCCACGTGTT	TAGCAGCAGGTGAGTGGGGC
Icam1	ACCCACCCCGCAGGTCCAAT	CAGCCGAGGACCATACAGCACG
Tnf	TCGGGGTGATCGGTCCCCAA	GGTGGTTTGCTACGACGTGGG
Trail	GGAAGACCTCAGAAAGTGGCAG	TTTCCGAGAGGACTCCCAGGAT
Ifna	GGATGTGACCTTCCTCAGACTC	ACCTTCTCCTGCGGGAATCCAA
Ifnb1	AAGAGTTACACTGCCTTTGCCATC	CACTGTCTGCTGGTGGAGTTCATC
Cxcl9	CCTAGTGATAAGGAATGCACGATG	CTAGGCAGGTTTGATCTCCGTTC
Cxcl10	AGTGCTGCCGTCATTTTCTG	ATTCTCACTGGCCCGTCAT
